# Gastric vagal afferent signaling to the basolateral amygdala mediates anxiety-like behaviors in experimental colitis mice

**DOI:** 10.1172/jci.insight.161874

**Published:** 2023-06-22

**Authors:** Chin-Hao Chen, Tsung-Chih Tsai, Yi-Jen Wu, Kuei-Sen Hsu

**Affiliations:** 1 Institute of Basic Medical Sciences,; 2 Institute of Clinical Medicine,; 3 Department of Neurology, and; 4 Department of Pharmacology, College of Medicine, National Cheng Kung University, Tainan, Taiwan.

**Keywords:** Neuroscience, Inflammatory bowel disease, Mouse models, Neurological disorders

## Abstract

Inflammatory bowel disease (IBD) is a relapsing-remitting disorder characterized by chronic inflammation of the gastrointestinal (GI) tract. Anxiety symptoms are commonly observed in patients with IBD, but the mechanistic link between IBD and anxiety remains elusive. Here, we sought to characterize gut-to-brain signaling and brain circuitry responsible for the pathological expression of anxiety-like behaviors in male dextran sulfate sodium–induced (DSS-induced) experimental colitis mice. We found that DSS-treated mice displayed increased anxiety-like behaviors, which were prevented by bilateral GI vagal afferent ablation. The locus coeruleus (LC) is a relay center connecting the nucleus tractus solitarius to the basolateral amygdala (BLA) in controlling anxiety-like behaviors. Chemogenetic silencing of noradrenergic LC projections to the BLA reduced anxiety-like behaviors in DSS-treated mice. This work expands our understanding of the neural mechanisms by which IBD leads to comorbid anxiety and emphasizes a critical role of gastric vagal afferent signaling in gut-to-brain regulation of emotional states.

## Graphical abstract

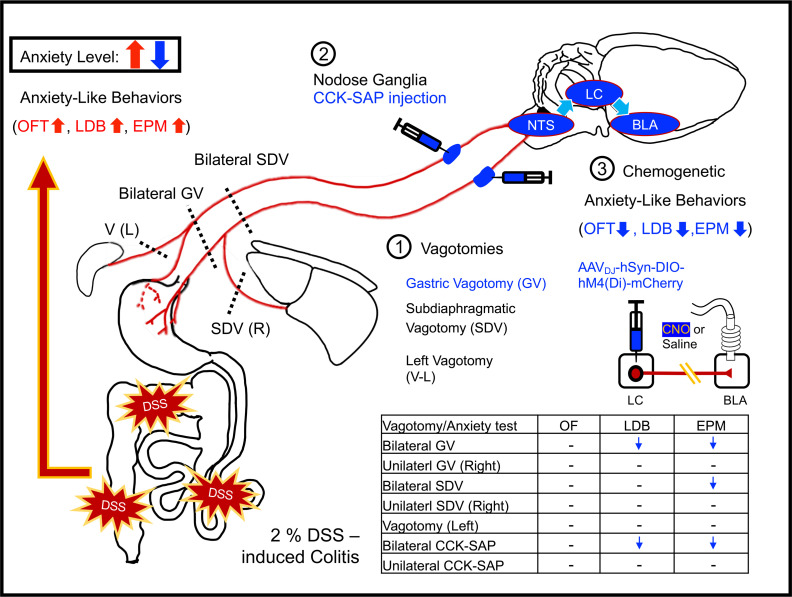

## Introduction

Inflammatory bowel disease (IBD), mainly comprising ulcerative colitis (UC) and Crohn’s disease (CD), is a chronic, relapsing-remitting and progressive inflammatory disorder of the gastrointestinal (GI) tract. Common symptoms of IBD include diarrhea, rectal bleeding, abdominal pain, fatigue, and weight loss (1). The exact etiology and pathogenesis of IBD remain unclear but involve a complicated interaction between the genetic, environmental or microbial factors, and immunological abnormalities (2). In addition to cardinal symptoms, anxiety is increasingly recognized as an important comorbidity in patients with IBD. It is estimated in cohorts that patients with IBD have a 3- to 6-fold increased risk of developing anxiety disorders compared with the general population (3, 4). Anxiety is estimated to have a 29%–35% prevalence in people affected by IBD during periods of remission and a prevalence as high as 80% during relapses (5, 6). The comorbid anxiety can substantially reduce patients’ quality of life and contribute significantly to the cost of IBD (7, 8). Despite their high prevalence and cooccurrence, effective treatment is still limited, and a better understanding of their pathophysiological convergence for uncovering new therapeutic strategies is required.

Numerous chemically induced colitis animal models exist for studying human IBD mechanistically (9). The dextran sulfate sodium–induced (DSS-induced) colitis is the most widely used IBD model because of its similarities to human diseases in terms of clinical and histopathological characteristics (10, 11). DSS treatment disrupts the intestinal epithelium and facilitates the invasion of bacteria into the mucosa, resulting in stimulating immune cells and secretion of proinflammatory cytokines and chemokines (12). Consistent with clinical features of patients with IBD, rodents treated with DSS showed increased anxiety-like behaviors (13–16). Despite the existing evidence implicating persistent central inflammation and altered brain excitability to the emergence of anxiety symptoms (15, 16), little is known about what gut-to-brain signaling, brain regions and neural circuits mediate the pathological expression of anxiety-like behaviors in a DSS-induced colitis model. Bridging this gap will edify strategies for treatment of comorbid anxiety in patients with IBD.

The gut and brain form the gut-brain axis through bidirectional neural, hormonal, and immunological communications. The vagus nerve is the major neuronal component of the gut-brain axis for conveying visceral information to the brain. Vagal afferent neurons synapse bilaterally on neurons within ventromedial areas of the nucleus tractus solitarius (NTS), where visceral signals are transmitted to several brainstem nuclei and forebrain structures (17–19). In the brain, the basolateral amygdala (BLA) is a key neuroanatomical structure implicated in the modulation of anxiety (20, 21). Optogenetic activation or inhibition of glutamatergic projection neurons in the BLA can elicit bidirectional control of anxiety-like behaviors (22). Importantly, recent functional magnetic resonance imaging studies revealed aberrant amygdala activity in patients with IBD (23), suggesting that amygdala may represent a plausible neural substrate of comorbid anxiety in IBD. While the NTS is functionally connected to the BLA (24), it remains unclear whether NTS neurons synaptically communicate to the BLA directly or via a polysynaptic pathway involving the noradrenergic projections of the locus coeruleus (LC) (25). Indeed, previous work has demonstrated that NTS neurons project directly to the LC (26), the LC-norepinephrine (LC-NE) system heavily innervates the BLA, and overactivation of the LC-BLA pathway increases anxiety-like behaviors (27, 28).

This study sought to use the DSS-treated mouse model to trace gut-to-brain signaling, brain regions, and neural circuits recruited for the emergence of anxiety-like behaviors on the acute phase of experimental colitis. We hypothesized that GI-derived vagal sensory signaling contributes to heightened anxiety-like behaviors in DSS-induced colitis mice and that the LC acts as a relay connecting the NTS to the BLA in mediating anxiety-like behaviors. Observations show that DSS-induced colitis resulted in increased anxiety-like behaviors, as measured by the open field (OF), light/dark box (LDB), elevated plus maze (EPM), and novelty suppressed feeding (NSF) tests, which were prevented by bilateral GI vagal afferent ablation. We demonstrate that the NTS-LC-BLA pathway contributes to mediate anxiety-like behaviors and that chemogenetic silencing of noradrenergic LC projections to the BLA ameliorates anxiety-like behaviors in DSS-treated mice. We report a previously undefined gut-to-brain neural circuit underlying comorbid anxiety associated with experimental colitis.

## Results

### DSS administration in mice results in colitis.

**Figure 1:**
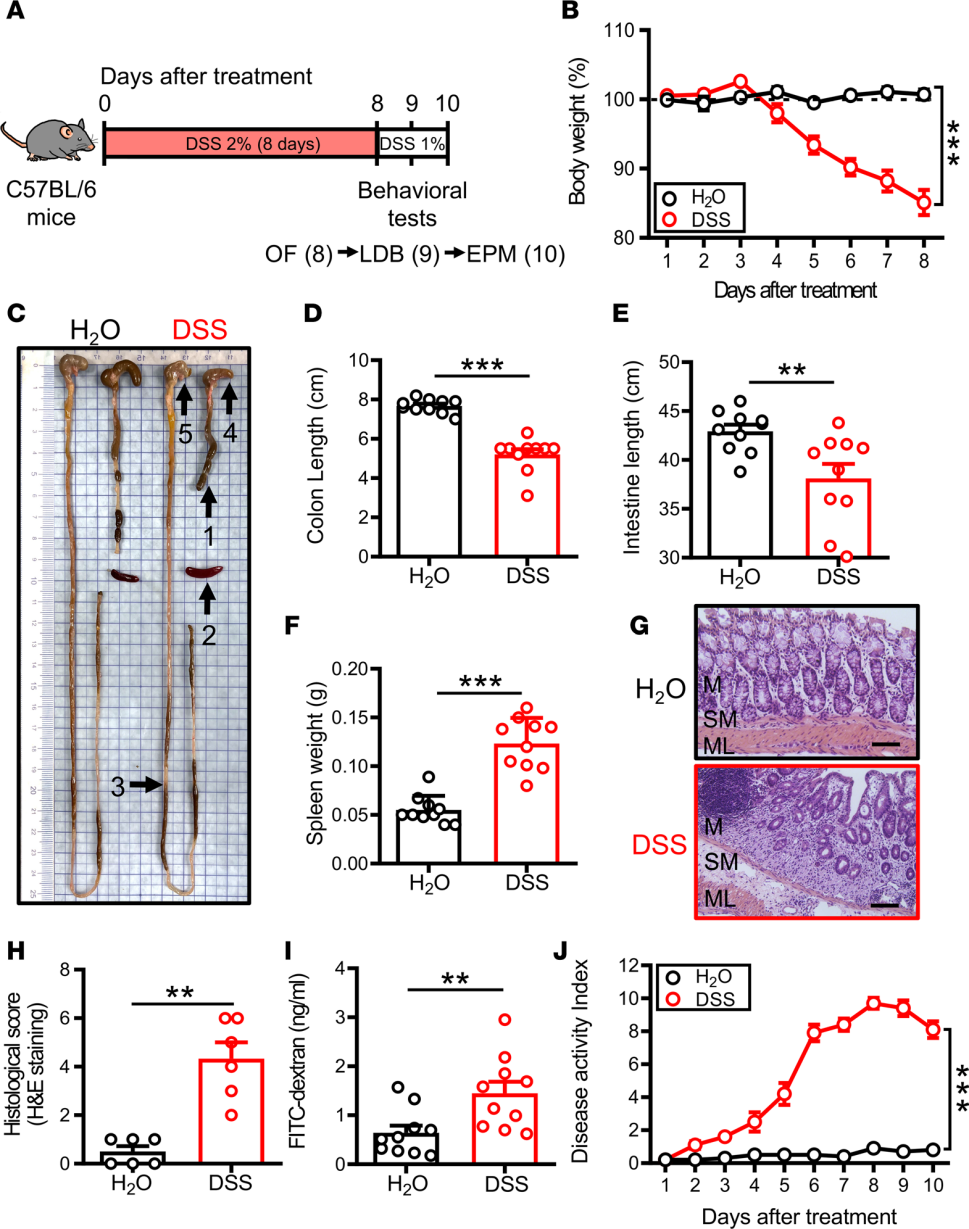
DSS induces acute colitis in mice. (**A**) Schematic of the experimental timeline. Mice received 2% DSS in drinking water for 8 days, followed by a maintenance dose of 1% for 2 days. All groups were subjected to behavioral tests on day D8, D9 ,and D10 before sacrifice. (**B**) Body weights recorded during the experimental period (H_2_O: *n* = 10; DSS: *n* = 10). (**C**) Gross morphology image of H_2_O- or DSS-treated mice on D11 after administration. Arrows indicate: (arrow 1) shortened colon; (arrow 2) enlarged spleen; (arrow 3) shortened small intestine; (arrow 4) shrink cecum; and (arrow 5) stomach. (**D**–**F**) Quantification of colon length, small intestine length, and spleen weight on D11 in mice receiving H_2_O or DSS treatment (H_2_O: *n* = 10; DSS: *n* = 10). (**G** and **H**) H&E staining and histological score of colonic sections were assessed on D11 in mice receiving H_2_O or DSS treatment (H_2_O: *n* = 6; DSS: *n* = 6). M, mucosa layer; SM, submucosa layer; and ML, muscular layer. Scale bar: 200 μm. (**I**) Quantification of FITC-dextran permeability on D11 receiving H_2_O or DSS treatment (H_2_O: *n* = 10; DSS: *n* = 10). (**J**) DAI score was applied to evaluate the severity of DSS-induced colitis recorded from D1 to D10 (H_2_O: *n* = 10; DSS: *n* = 10). DAI score was calculated by total score (body weight + stool consistency + rectal bleeding). Data represent the mean ± SEM. **P < 0.01 and *** *P* < 0.001 by 2-way, repeated measures (RM) ANOVA (**B** and **J**), Mann-Whitney *U* test (**D** and **H**), and 2-tailed unpaired Student’s *t* test (**E**, **F**, and **I**).

To explore the mechanistic link between IBD and anxiety, we utilized an established mouse model consisting of administration of DSS (2%) via the drinking water for 8 consecutive days, followed by a maintenance dose of 1% throughout the study (Figure 1A). The lower maintenance dose was used to maintain the extent and severity of colitis symptoms without weakening mice during behavioral tests. As expected, administration of DSS resulted in significant body weight loss in all mice, starting from day 5 (D5) and reaching a maximum level by D8 (Figure 1B). On D11, mice were sacrificed for further investigation. Correspondingly, the lengths of colon and the small intestine were significantly shorter in the DSS group compared with the H_2_O group (Figure 1, C–E). In addition, spleen was significantly enlarged in the DSS group compared with the H_2_O group (Figure 1, C and F). Histological analysis of colons from the DSS group showed extensive epithelial destruction, mucosal ulceration, and inflammatory cell infiltration to the mucosa layer, which were absent in the H_2_O group (Figure 1G). The histological damage scored by H&E staining was significantly increased in the DSS group compared with the H_2_O group (Figure 1H). DSS administration resulted in damage to the intestine. We assessed the intestinal barrier integrity by orally administering fluorescein isothiocyanate–dextran (FITC-dextran) to mice and measuring its serum levels. Results show that serum levels of FITC-dextran in DSS-treated mice were significantly higher than in the H_2_O group (Figure 1I). The colitis severity was also quantified using the disease activity index (DAI) scoring body weight loss, stool consistency, and rectal bleeding (29). The DSS group showed significantly higher DAI values compared with the H_2_O group (Figure 1J).

### DSS-induced colitis results in increased anxiety-like behaviors.

**Figure 2:**
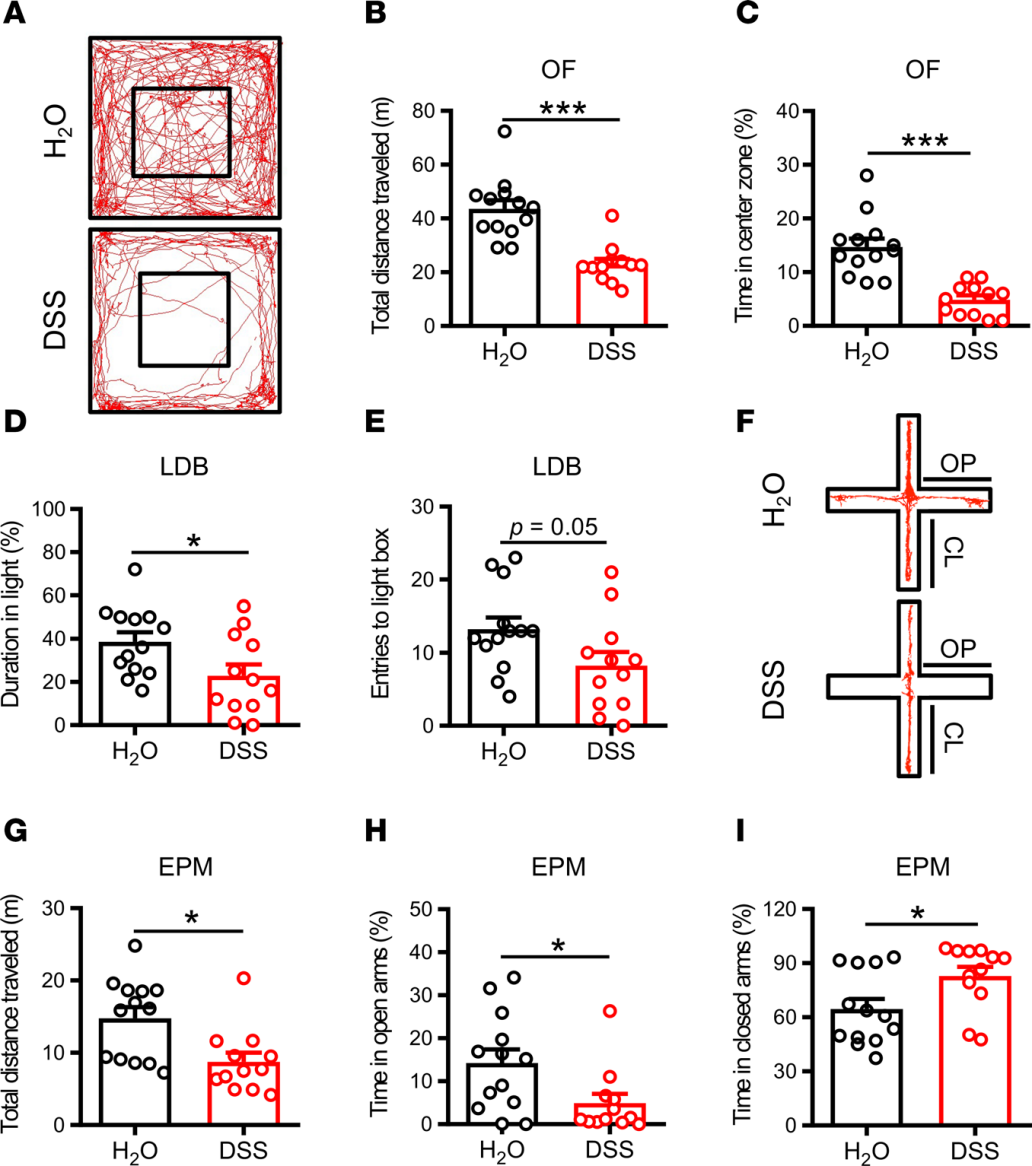
DSS-induced colitis causes anxiety-like behaviors. (**A**) Representative movement traces of H_2_O- and DSS-treated mice in the OF test. (**B** and **C**) Bar graphs comparing the effects of H_2_O and DSS treatment on the total distance traveled and the percentage of time spent in central zone (H_2_O: *n* = 13; DSS: *n* = 12) in the OF test. (**D** and **E**) Bar graphs comparing the effects of H_2_O and DSS treatment on the duration in the light box and the number of entries into the light box (H_2_O: *n* = 13; DSS: *n* = 12) in the LDB test. (**F**) Representative movement traces of H_2_O- and DSS-treated mice in the EPM test. (**G**–**I**) Bar graphs comparing the effects of H_2_O and DSS treatment on the total distance traveled, the percentage of time spent in the open arms, and the percentage of time spent in the closed arms (H_2_O: *n* = 13; DSS: *n* = 12) in the EPM test. Data represent the mean ± SEM. *P < 0.05 and *** *P* < 0.001 by Mann-Whitney *U* test (**B**, **G**, and **H**) and 2-tailed unpaired Student’s *t* test (**C**, **D**, **E**, and **I**).

Four widely used behavioral tests (OF, LDB, EPM, and NSF) were used to assess anxiety-like behaviors. In the OF test, the DSS group showed a significant decrease in total distance traveled (Figure 2, A and B) and spent less time in the central zone of the field (Figure 2C) compared with the H_2_O group. In the LDB test, the DSS group spent less time in the light box (Figure 2D) and showed a decrease in the number of entries into the light box (Figure 2E) compared with the H_2_O group. In the EPM test, the DSS group showed a significant decrease in total distance traveled (Figure 2, F and G) and spent less time in the open arms (Figure 2H) and more time in the closed arms (Figure 2I) compared with the H_2_O group. Since decreased locomotor activity was observed in the DSS group, such effects might interfere with measures of anxiety-like behaviors that require locomotion. To exclude this possibility, we further analyzed anxiety-related risk assessment behaviors in the OF, EPM, and LDB tests. In comparison with the control H_2_O group, the DSS group showed decreased number of rearing during the period of the OF and EPM tests and a decreased number of stretch attend postures during the period toward the open arms in the EPM, but no difference was observed in the number of nose pokes in the LDB test (Supplemental Figure 1, B–E; supplemental material available online with this article; https://doi.org/10.1172/jci.insight.161874DS1). Furthermore, we also used the NSF test, a model of anxiety that does not heavily rely on locomotor activity (30). Consistent with our other anxiety-like behavioral tests, the DSS group showed a sign of anxiety as demonstrated by increased in the latency to eat compared with the H_2_O group (Supplemental Figure 2, A and B). In addition, we confirmed no significant difference between groups in total distance traveled in their home cage activity (HCA) (Supplemental Figure 2C). These findings indicate that DSS-induced colitis increases anxiety-like behaviors.

### Bilateral gastric vagotomy reduces anxiety-like behaviors in DSS-induced colitis mice.

**Figure 3:**
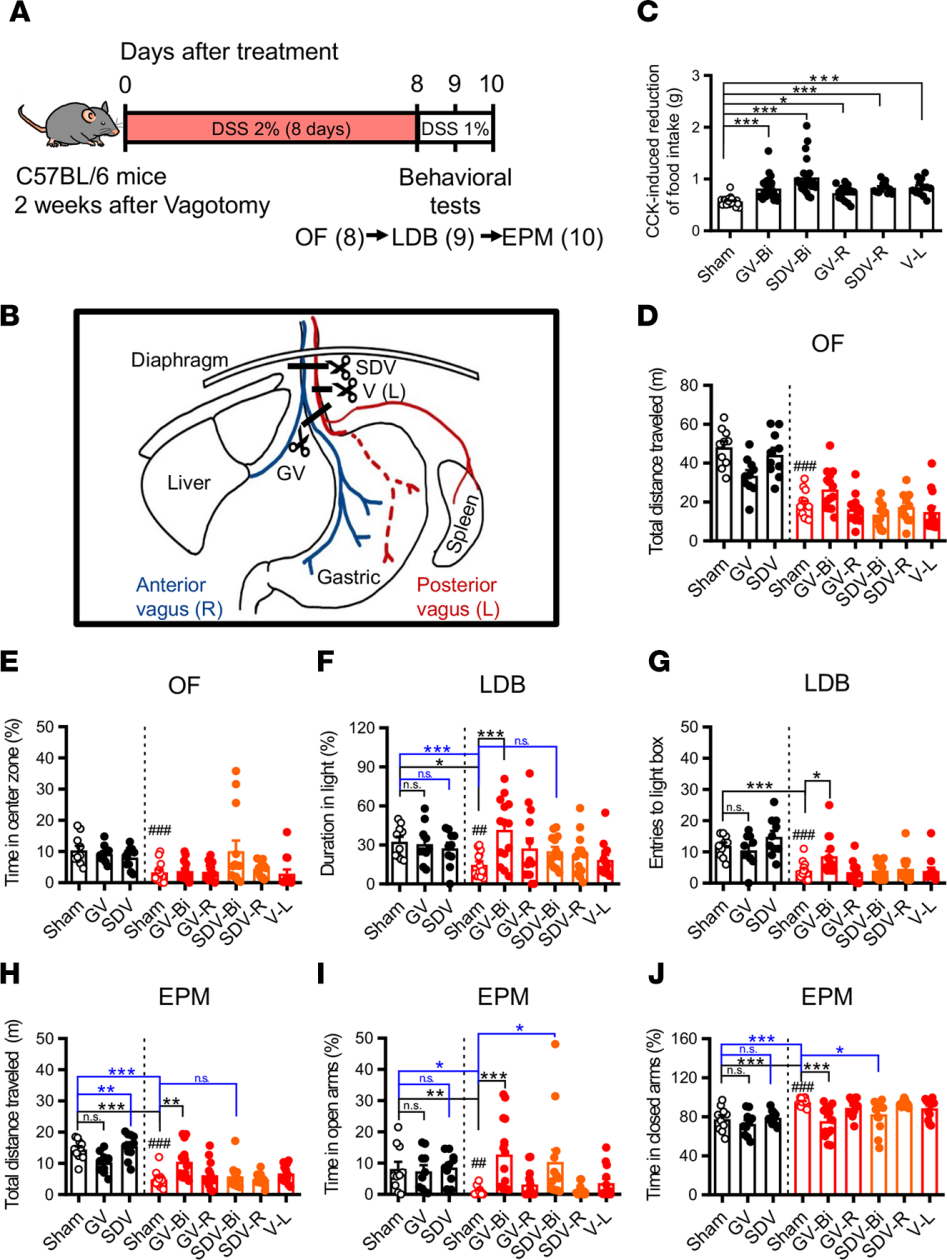
Gastric vagotomy reduces anxiety-like behaviors in DSS-induced colitis mice. (**A**) Schematic of the experimental timeline. (**B**) Schematic illustration of vagotomy methods. Bilateral gastric vagotomy (GV-Bi), bilateral subdiaphragmatic vagotomy (SDV-Bi), and vagotomy of left vagus (V-L). Blue represents anterior (right side) and red represents posterior (left side) of vagus. (**C**) Bar graph comparing the CCK-induced reduction of food intake (sham: *n* = 23; GV-Bi: *n* = 24; SDV-Bi: *n* = 23; GV-R: *n* = 13; SDV-R: *n* = 12; V-L: *n* = 13). (**D**–**J**) Black bars represent H_2_O groups, and red and orange bars represent DSS-treated groups (sham: *n* = 10; GV-Bi: *n* = 10; SDV-Bi: *n* = 10; sham-DSS: *n* = 13; GV-Bi-DSS: *n* = 14; GV-R-DSS: *n* = 13; SDV-Bi-DSS: *n* = 13; SDV-R-DSS: *n* = 12; V-L-DSS: *n* = 13). (**D** and **E**) Bar graphs comparing the effects of sham and vagotomy operations on the total distance traveled and the percentage of time spent in central zone in the OF test. (**F** and **G**) Bar graphs comparing the effects of sham and vagotomy operations on the duration in the light box and the number of entries into the light box in the LDB test. (**H**–**J**) Bar graphs comparing the effects of sham and vagotomy operations on the total distance traveled, the percentage of time spent in the open arms, and the percentage of time spent in the closed arms in the EPM test. Data represent the mean ± SEM. **^##^***P* < 0.01 and **^###^***P* < 0.001 by 2-tailed unpaired Student’s *t* test (**D**, **E**, **F**, and **J**) and Mann-Whitney *U* test (**G**–**I**); *P < 0.05, **P < 0.01, and ***P < 0.001 by Kruskal-wallis test with Dunn’s test (**C**) and 2-way ANOVA with Tukey’s multiple-comparison test (**F**–**J**).

Although it is assumed that vagal afferents are critical for mediating gut-brain communication to modulate emotional behaviors (31, 32), evidence linking abnormal vagal signaling with the emergence of comorbid anxiety in colitis remains limited (33, 34). To distinctly examine whether signaling from different branches of the vagus nerve contributes to anxiety-like behaviors in colitis, we compared unilateral or bilateral gastric vagotomy (GV) and subdiaphragmatic vagotomy (SDV) with sham-operated colitis mice in the OF, LDB, and EPM tests. The experimental procedure is depicted in Figure 3A. Two weeks after vagotomy, mice were subjected to the protocol of DSS-induced colitis. The schematic shows lesioning of the vagal branches targeted by different types of vagotomy procedures (Figure 3B). Completeness of vagotomy was functionally verified using a CCK-induced satiety test at 10 days after surgery. As expected (35, 36), significant reductions in CCK-induced reduction of food intake were observed in mice receiving different types of vagotomy procedures compared with sham-operated mice (Figure 3C). The effect of vagotomy on DSS-induced colitic characteristics was also assessed. We found that body weight loss, shorter small intestine length, enlarged spleen, colon damage, increased intestinal permeability, and disease activity were unaltered by bilateral GV. However, bilateral GV-DSS group showed shorter colon length compared with sham-DSS group of colitis mice. Moreover, in the bilateral SDV group, severity of colitis was partially increased after DSS administration compared with GV group observed by shorter colon and small intestine length and higher intestinal permeability without significantly affecting colon damage, spleen weight, or disease activity progression (Supplemental Figure 3, A–I). In the OF, LDB, and EPM tests, the sham-DSS group showed increased anxiety-like behaviors accompanied by decreased total distance traveled in the OF and EPM tests (Figure 3, D–J). However, there were no differences between vagotomy-DSS groups and the sham-DSS group observed in the OF test (Figure 3, D and E). In the LDB test, only bilateral GV significantly increased the duration in the light box and the entries into the light box of DSS-induced colitis mice (Figure 3, F and G). In the EPM test, both bilateral GV and SDV increased the time spent in open arms and decreased the time spent in closed arms of DSS-induced colitis mice (Figure 3, I and J). However, only bilateral GV but not SDV increased total distance traveled in DSS-induced colitis mice compared with the sham-DSS group (Figure 3H). To exclude the interference of increased total distance traveled on interpretation of anxiolytic effect of GV and risk assessment behaviors in the OF, EPM, and LDB, the NSF and HCA tests of bilateral GV were assessed in DSS-induced colitis mice. In comparison with sham-DSS group, the GV-DSS group showed increased risk-assessment behaviors in the OF and the EPM tests, but no difference was observed in the number of nose pokes in the LDB test (Supplemental Figure 1, F–I). In the NSF test, the GV-DSS group showed a significant decrease in latency to eat compared with the sham-DSS group (Supplemental Figure 2E), and no significant difference in total distance traveled was observed in the HCA test (Supplemental Figure 2F). Because DSS-induced colitis also results in visceral pain (37, 38) and because chronic pain can provoke anxiety-like behaviors (39, 40), we next examined the effect of GV on mechanical pain sensitivity in the hind paw of DSS-induced colitis mice. As expected, administration of DSS resulted in a significant decrease in withdrawal threshold measured using the von Frey filament test in mice, starting from D3 and then remaining constant over time (Supplemental Figure 2D). However, there was no difference between sham-DSS and GV-DSS mice in their withdrawal threshold to mechanical stimulation (Supplemental Figure 2G). These findings suggest that bilateral but not unilateral GI vagal ablation is sufficient to reduce anxiety-like behaviors in DSS-induced colitis mice.

### Vagal deafferentation by cholecystokinin-saporin (CCK-SAP) reduces anxiety-like behaviors in DSS-induced colitis mice.

**Figure 4:**
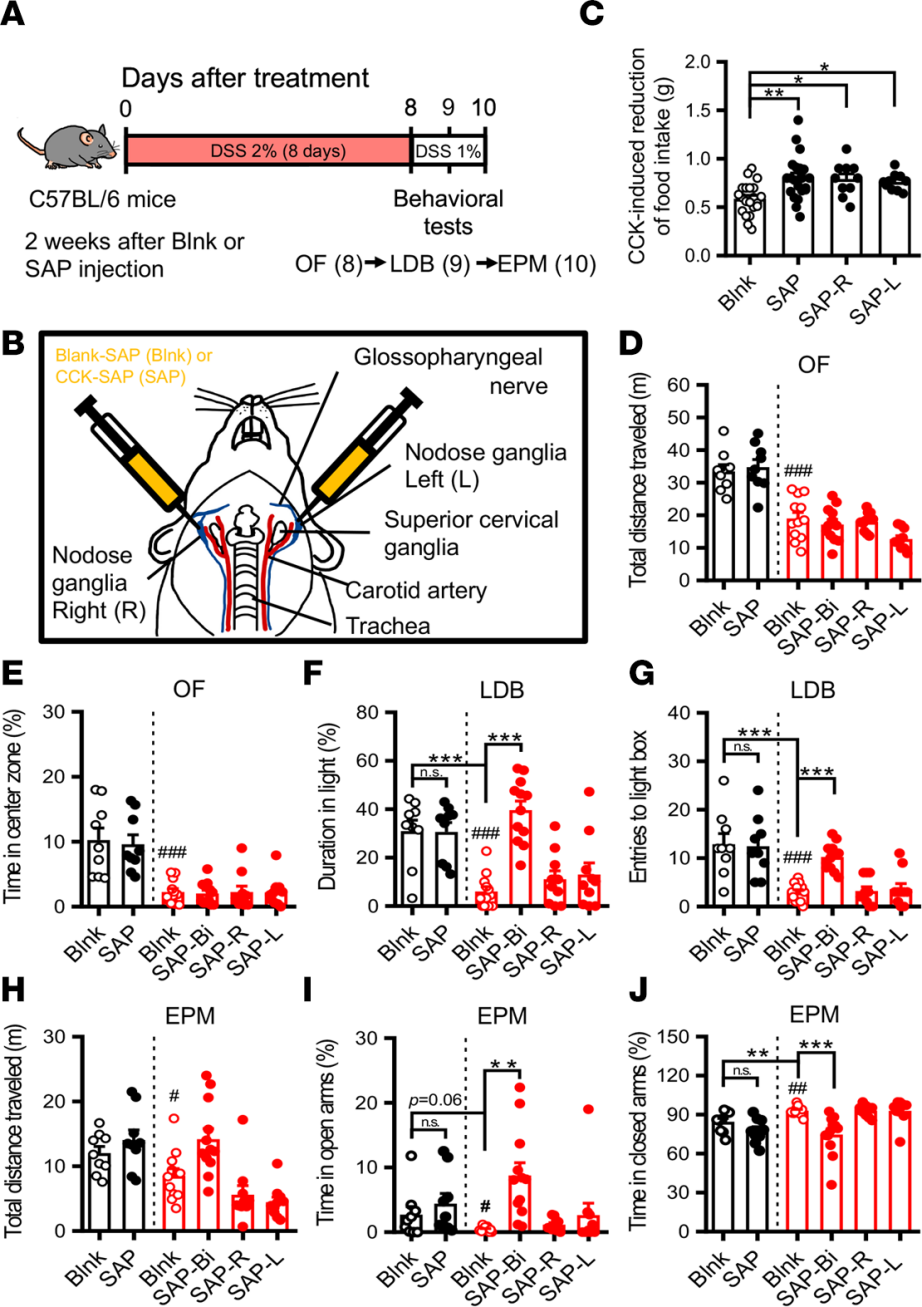
Targeted ablation of vagal afferent neurons by CCK-SAP injection reduces anxiety-like behaviors in DSS-induced colitis mice. (**A**) Schematic of the experimental timeline. (**B**) Schematic illustration of CCK-SAP nodose ganglia injections to specifically ablate GI vagal afferent signaling. (**C**) Bar graph comparing CCK-induced reduction of food intake in Blank-SAP–treated (Blnk) and CCK-SAP–treated (SAP) mice (Blank: *n* = 21; SAP: *n* = 21; SAP-Right: *n* = 10; SAP-Left: *n* = 10). (**D**–**J**) Black bars represent H_2_O groups, and red bars represent DSS-treated groups (Blnk: *n* = 9; SAP: *n* = 9; Blnk-DSS: *n* = 12; SAP-DSS: *n* = 12; SAP-R-DSS: *n* = 10; SAP-L-DSS: *n* = 10). (**D** and **E**) Bar graphs comparing the effects of SAP-Blank and CCK-SAP injections on the total distance traveled and the percentage of time spent in central zone in the OF test. (**F** and **G**) Bar graphs comparing the effects of SAP-Blank and CCK-SAP injections on the duration in the light box and the number of entries into the light box in the LDB test. (**H**–**J**) Bar graphs comparing the effects of SAP-Blank and CCK-SAP injections on the total distance traveled, the percentage of time spent in the open arms, and the percentage of time spent in the closed arms in the EPM test. Data represent the mean ± SEM. **^#^***P* < 0.05, **^##^***P* < 0.01, and **^###^***P* < 0.001 by 2-tailed unpaired Student’s *t* test (**D**, **E**, and **G**–**J**) and Mann-Whitney *U* test (**F**); *P < 0.05, **P < 0.01, and ***P < 0.001 1-way ANOVA with Dunn’s test (**C**) and 2-way ANOVA with Tukey’s multiple-comparison test (**F**, **G**, **I**, and **J**).

Surgical vagotomy eliminates bidirectional vagal signaling between the gut and the brain, since both sensory afferent and motor efferent fibers are excised. The CCK-SAP (SAP) approach has been shown to effectively eliminate ~80% of gut-innervating sensory vagal afferents to the brain while leaving intact brain-to-gut vagal motor signaling (41). To further validate the aforementioned findings, we injected SAP into unilateral or bilateral nodose ganglia (NDG) to selectively eliminate GI-derived vagal sensory afferent signaling, followed by DSS-induced colitis induction protocol (Figure 4A). The schematic shows the NG targeted by control Blank-SAP (Blnk) or SAP injection (Figure 4B). The effectiveness of SAP was verified by the blunted effect of CCK-induced food intake reduction (Figure 4C). We also took advantage of food intake–induced neuronal activity (c-Fos^+^) in the NTS and neuronal tracing signals in the NDG with i.p. injection of retrograde tracer Fluorogold (FG, 10 mg/kg) to confirm target efficiency and specificity of SAP injection to ablate GI vagal afferents as described previously (41). We found that the SAP group showed a significant reduction in numbers of c-Fos protein immunoreactive cells (Supplemental Figure 3, A–D) expressed in the NTS and Fluorogold immunoreactive cells expressed in the NDG (Supplemental Figure 4, E–G) 90 minutes after food intake compared with the Blnk group. In contrast, there was no significant difference between Blnk and SAP groups in the number of Fluorogold immunoreactive cells in the dorsal motor nucleus of the vagus (DMV) of the brainstem, the major source of vagal parasympathetic motor output neurons (Supplemental Figure 4, H–J). The effect of SAP injection on DSS-induced colitic characteristics was assessed. Compared with the Blnk-DSS group, severity of DSS-induced colitis was unaltered in the SAP-DSS group (Supplemental Figure 5). Following DSS administration, the Blnk-DSS group showed increased anxiety-like behaviors assessed by OF, LDB, and EPM tests (Figure 4, D–J). In the OF test, there were no differences between the Blnk-DSS group and SAP-DSS groups (bilateral and unilateral) in total distance traveled (Figure 4D) and time spent in the central zone of the field (Figure 4E). In the LDB test, only the bilateral SAP-DSS group spent more time in the light box (Figure 4F) and showed an increase in the number of entries into the light box (Figure 4G) compared with the Blnk-DSS group. In the EPM test, there were no differences between the Blnk-DSS group and bilateral SAP-DSS group in total distance traveled (Figure 4H), whereas the bilateral SAP-DSS group spent more time in the open arms (Figure 4I) and less time in the closed arms (Figure 4J) compared with the Blnk-DSS group. Moreover, in comparison with the Blnk-DSS group, SAP-DSS mice also showed increased risk assessment behaviors in the OF and EPM tests, but no difference was observed in the number of nose pokes in the LDB test (Supplemental Figure 1, J–M). In the NSF test, SAP-DSS mice showed reduced latency to eat compared with Blnk-DSS mice (Supplemental Figure 2H). No difference between Blnk-DSS and SAP-DSS mice in their total distance traveled in HCA test (Supplemental Figure 2I) and withdrawal threshold to mechanical stimulation were observed (Supplemental Figure 2J). These findings suggest that bilateral gut vagal sensory signaling contributes to increased anxiety-like behaviors in DSS-induced colitis mice.

### Gut-to-brain neural circuit for anxiety-like behaviors in DSS-induced colitis mice.

**Figure 5:**
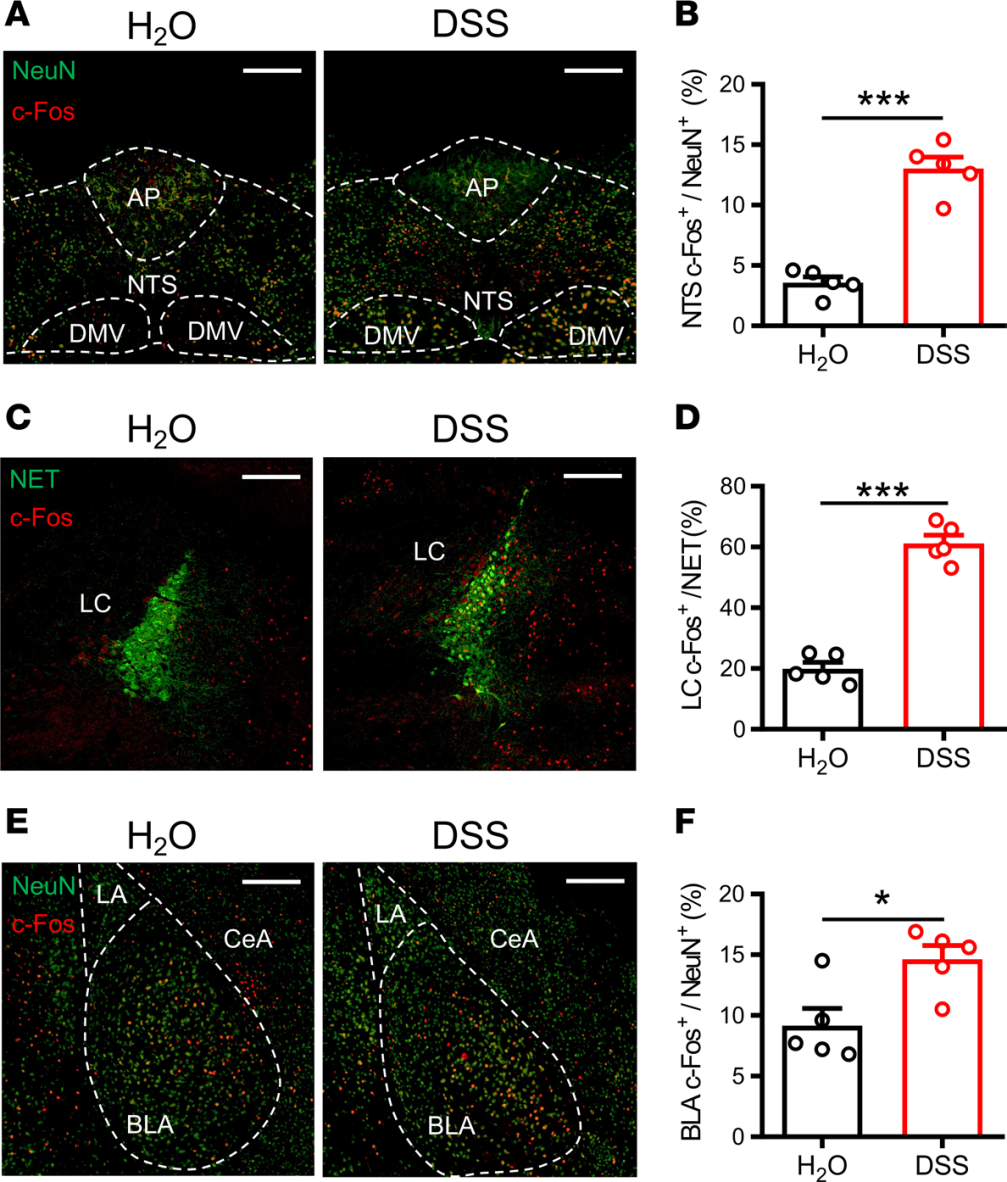
Profiling of c-Fos expression with multiple brain regions after the EPM test in DSS-induced colitis mice. Representative images and quantitative analysis of c-Fos–labeled cells in H_2_O- and DSS-treated mice 90 minutes after the EPM test within the nucleus tractus solitarius (NTS, *n* = 5 in each group) (**A** and **B**), locus coeruleus (LC, *n* = 5 in each group) (**C** and **D**), and basolateral amygdala (BLA, *n* = 5 in each group) (**E** and **F**). Scale bar: 200 μm. AP, area postrema; DMV, dorsal motor nucleus of the vagus; LA, lateral amygdala; CeA, central nucleus of the amygdala. Data represent the mean ± SEM. *P < 0.05 and ***P < 0.001 by 2-tailed unpaired Student’s *t* test.

We next sought to determine brain regions and neural circuit mechanisms underlying anxiety-like behaviors in colitis mice. Because the NTS is the first CNS site that receives GI-derived vagal sensory afferent inputs (17–19) and LC-BLA projections have been implicated in mediating anxiety-like behaviors (27, 28), we chose the NTS, LC, and BLA for neuronal activation analysis 90 minutes after the end of EPM test. The c-Fos was analyzed to map neuronal activity. In comparison with the H_2_O group, the DSS group showed elevated numbers of c-Fos protein immunoreactive cells expressed in the NTS (Figure 5, A and B), LC (Figure 5, C and D), and BLA (Figure 5, E and F). In addition, we found that both GV-DSS and SAP-DSS groups showed a significant reduction in numbers of c-Fos protein immunoreactive cells expressed in the NTS (Supplemental Figure 6, A and B, and Supplemental Figure 7, A and B), LC (Supplemental Figure 6, C and D, and Supplemental Figure 7, C and D), and BLA (Supplemental Figure 6, E and F, and Supplemental Figure 7, E and F) 90 minutes after the end of EPM test compared with sham-DSS and Blnk-DSS groups.

**Figure 6:**
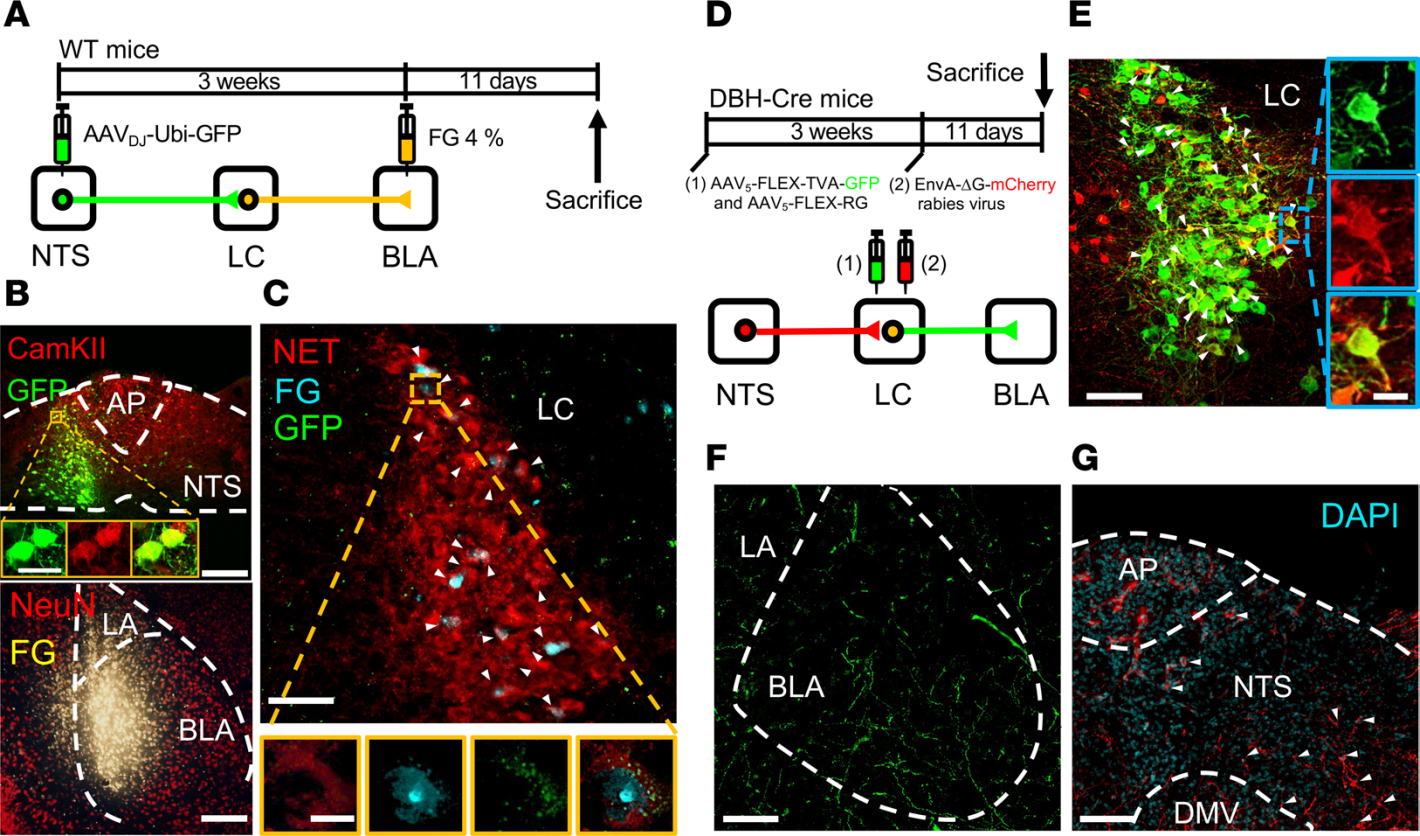
The LC acts as a relay region connecting the nucleus of the NTS to the BLA. (**A**) Schematic of the experimental design. Fluorogold (FG, 4%) was injected into the BLA, and AAV_DJ_-Ubi-GFP was injected into the NTS. (**B**) Top: Representative image showing GFP signals (green) and CaMKIIa (red) colocalized in NTS neurons. Bottom: representative image showing FG signals in the BLA. Scale bar: 200 μm. (**C**) Representative image showing FG-labeled LC neurons were immunopositive for norepinephrine transporter (NET). Scale bar: 100 μm. Augmented figures (bottom) showing FG-labeled neurons in rectangle area. Scale bar: 10 μm. (**D**) Schematic of viral injections. AAV_5_-FLEX-TVA-GFP, AAV_5_-FLEX-RG, and EnvA-ΔG-mCherry were unilaterally injected into the LC. Three weeks after stereotaxic injection of AAV_5_-FLEX-TVA-GFP and AAV_5_-FLEX-RG, EnvA-ΔG-mCherry was injected into the LC of DBH-Cre mice. Eleven days later, mice were sacrificed and perfused, and slices were prepared for fluorescence microscopy. (**E**) Representative image showing GFP expression in the LC. White arrowheads indicate cell doubled for GFP (green) and mCherry (red). Scale bar: 100 μm. (**F**) GFP signals of axonal projections were observed in the BLA. Scale bar: 100 μm. (**G**) Representative image of mCherry^+^ presynaptic cells in the NTS in DBH-Cre mice. White arrowheads indicate cell doubled for DAPI (blue) and mCherry (red). Scale bar: 100 μm. Data was replicated in 4 mice.

To assess the synaptic connection between NTS and BLA, we employed anterograde and retrograde tracing methods to identify whether the NTS communicates directly with the BLA or indirectly via the LC. To label NTS-LC projections, we first injected an anterograde tracer AAV_DJ_-Ubi-EGFP unilaterally into the NTS. Three weeks after injection, a retrograde tracer FG (4 %) was injected into the BLA in the same cohort of mice (Figure 6A). Eleven days later, confocal imaging showed that retrogradely labeled neurons were abundant in the posterior portion of the LC and were immunopositive for NE transporter (NET). Green fluorophore anterogradely labeled axons originating from the NTS was found in apposition to FG-labeled cells in the LC that were retrogradely labeled from the BLA (Figure 6, B and C). To further confirm that NTS projection neurons form direct synaptic connectivity with LC NE neurons, we performed monosynaptic input tracing using a monosynaptic rabies virus–tracing approach. We first injected a viral cocktail (AAV_5_-FLEX-TVA-GFP and AAV_5_-FLEX-RG) unilaterally into the LC of DBH-Cre mice, followed 3 weeks later by injection of G-deleted pseudotyped rabies virus expressing mCherry (EnvA-ΔG-mCherry) to infect Cre-expressing neurons (Figure 6D). As expected, we identified starter cells (GFP^+^/mCherry^+^) in the LC, mCherry^+^ monosynaptic input neurons (mCherry^+^) in the NTS, and GFP^+^ axon fibers in the BLA (Figure 6, E–G). To further characterize whether DSS administration affects circuit connectivity of the NTS-LC-BLA pathway, we injected anterograde tracer AAV_DJ_-CaMKIIα-mCherry unilaterally into the right or left NTS. Three weeks after injection, retrograde tracer FG (4 %) was injected into the ipsilateral BLA followed by DSS administration for 8 days (Supplemental Figure 8A). Confocal imaging showed no difference in the number of NET neurons in the left or right LC immunopositive for retrogradely labeled FG signals and anterogradely labeled red fluorophore for axons from the NTS (Supplemental Figure 8, B–F). In addition, the NTS only detected a scarce signal of retrogradely labeled FG from the BLA (Supplemental Figure 8C). These results suggest that NTS neurons connect to BLA majorly indirect through LC NE neurons and that DSS administration doesn’t affect the circuit connectivity of NTS-LC-BLA of both hemispheres.

**Figure 7:**
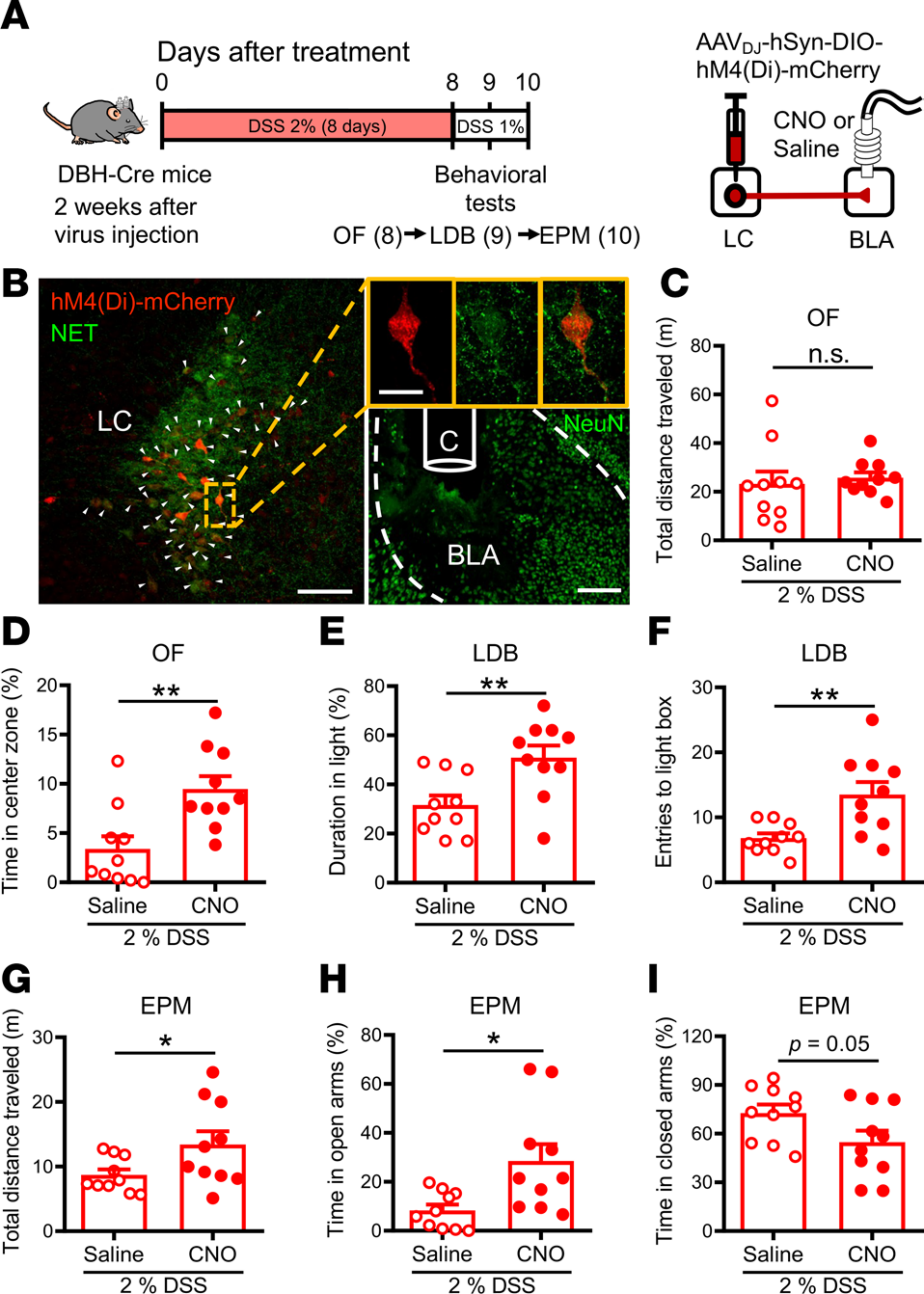
Chemogenetic inhibition of LC-NE projections to the BLA reduces anxiety-like behaviors in DSS-induced colitis mice. (**A**) Left: schematic of the experimental timeline. Right: AAV_DJ_-hSyn-DIO-hM4Di-mCherry was injected into the LC of DSS-treated DBH-Cre mice, and CNO was microinfused into the BLA. (**B**) Representative images showing mCherry expression in the LC (left, red) and cannula implantation site in the BLA (right; bottom). Scale bar: 100 μm. The mCherry-labeled LC neurons were immunopositive for NET (green) indicated by arrowheads. Augmented figures (right) show mCherry-labeled neurons in rectangle area. Scale bar: 20 μm. (**C** and **D**) Bar graphs comparing the effects of saline and CNO injection on the total distance traveled (*n* = 10 in each group) (**C**) and the percentage of time spent in central zone (*n* = 10 in each group) (**D**) in the OF test. (**E** and **F**) Bar graphs comparing the effects of saline and CNO injection on the duration in the light box (*n* = 10 in each group) (**E**) and the number of entries into the light box (*n* = 10 in each group) (**F**) in the LDB test. (**G**–**I**) Bar graphs comparing the effects of saline and CNO injection on the total distance traveled (*n* = 10 in each group) (**G**), the percentage of time spent in the open arms (*n* = 10 in each group) (**H**), and the percentage of time spent in the closed arms (*n* = 10 in each group) (**I**) in the EPM test. Data represent the mean ± SEM. *P < 0.05 and **P < 0.01 by 2-tailed unpaired Student’s *t* test.

We then determined the role of the LC-BLA pathway in anxiety-like behaviors in colitis mice by using a chemogenetic approach. We bilaterally injected the AAV_DJ_-hSyn-DIO-hM4Di-mCherry into the LC of DSS-treated DBH-Cre mice and locally infused clozapine N-oxide (CNO) into the BLA to selectively target BLA-projecting LC-NE^+^ neurons (Figure 7A). Two weeks after AAV injection followed by receiving DSS treatment, DSS-treated DBH-Cre mice were subjected to anxiety-like behavior tests after local injection of saline or CNO into the BLA. Post hoc histological examination of brain sections revealed robust and bilateral coexpression of hM4Di with the NE neuronal maker NET in the LC (Figure 7B). The severity of DSS-induced colitis in DBH-Cre mice was assessed, and no differences were observed between the CNO-DSS group and saline-DSS group (Supplemental Figure 9). In the OF test, DSS mice that received a CNO injection showed a significant increase in time spent in the central zone of the field compared with saline injection (Figure 7D), but there was no difference between saline-DSS and CNO-DSS groups in total distance traveled (Figure 7C). In the LDB test, the CNO-DSS group spent more time in the light box (Figure 7E) and showed an increased number of entries into the light box (Figure 7F) compared with the saline-DSS group. In the EPM test, the CNO-DSS group showed a significant increase in total distance traveled (Figure 7G) and spent more time in the open arms (Figure 7H) compared with the saline-DSS group. Although there was a trend toward less time spent in the closed arms in CNO-DSS group compared with saline-DSS group, the difference did not reach statistical significance (Figure 7I). Post hoc histological examination of brain sections 90 minutes after EPM test showed reduced numbers of c-Fos protein immunoreactive cells in the LC after CNO infusion (Supplemental Figure 10, A and B), whereas no significant difference was observed between saline-DSS and CNO-DSS groups in the number of c-Fos protein immunoreactive cells in the NTS (Supplemental Figure 10, C and D).

## Discussion

Anxiety-related symptoms occur frequently in patients with IBD. Despite clinical evidence, investigations of mechanistic links between IBD and comorbid anxiety remain limited. In this study, using a combination of neuronal labeling and pathway tracing, chemogenetic approach and behavioral assays, we provide evidence that acute DSS-induced colitis leads to activation of bilateral gastric vagal afferents to transmit peripheral signals to hyperexcite the central NTS-LC-BLA pathway, thus promoting anxiety-like behaviors. We also demonstrate the importance of LC-NE projections to the BLA in mediating anxiety-like behaviors. These findings identify a previously unrecognized brain pathway of vagal origin underpinning the emergence of anxiety symptoms in experimental colitis during acute phase.

The gut-brain axis encompasses bidirectional communication between the gut and the brain via neural, hormonal, and immune signaling mechanisms. Previous work in identifying mechanisms underlying comorbid anxiety in the DSS-induced colitis model has mainly focused on central inflammation caused by local inflammatory responses in the gut. It has been shown that DSS-induced colitis increases cortical proinflammatory cytokines and microglial immunoreactivity accompanied by anxiety-like behavior (16), but the causal link between them is still unclear. Very few studies to date have systematically assessed the role of vagal afferent signaling in colitis-induced comorbid anxiety (33, 34), and there is no existing study, as far as we know, that has been conducted to identify distinct vagal neural branches underpinning comorbid anxiety in the DSS colitis model. In agreement with previous studies (13–16), we found that DSS administration resulted in acute colitis accompanied by increased anxiety-like behaviors, as indicated by a decrease in the time spent in the central zone of the OF, the light box of the LDB and the open arms of the EPM. Similar to our findings, DSS administration has previously been shown to result in reduced locomotor activity as measured by distance traveled in the OF and EPM (15, 16). While it is possible that decreased locomotor activity might interfere with measures of anxiety-like behaviors in the OF and EPM, our results confirm previous findings that DSS-treated mice display anxiety-like behavior in the NSF test (42), which does not heavily rely on locomotor activity (30). Moreover, anxiety-related risk assessment behaviors in the OF and EPM were decreased in DSS-induced colitis mice, which were rescued by bilateral GV or vagal deafferentation by SAP. Therefore, the DSS-induced colitis mouse model is suited to identify mechanism by which intestinal inflammation modulates anxiety-like behaviors. More importantly, we provide causal evidence that GI-derived vagal sensory signaling plays an important role in developing anxiety-like behaviors in colitis. Indeed, we show that bilateral GI vagal ablation alleviates anxiety-like behaviors in DSS-induced colitis mice, as evidenced by measuring performance in the EPM and the LDB tests. These findings are consistent with a previous study by Bercik et al. (34), which reported that vagotomy can completely reverse the anxious phenotype in DSS-treated mice in the step-down test. Consequently, the vagus nerve may serve as an important conduit in the gut-brain axis responsible for transducing signals from the inflamed gut to bring about changes in the brain that lead to colitis-induced comorbid anxiety.

It has been reported previously that chronic colitis–induced visceral pain is often accompanied by increased anxiety-like behaviors (39, 40). In accordance with previous findings (37, 38), our data also indicate that administration of DSS resulted in the development of mechanical hypersensitivity in the hind paw of mice. However, GV and SAP had no effect on the development of mechanical hypersensitivity in DSS-induced colitis mice, suggesting that the increased anxiety-like behaviors observed in our acute colitis model are not causally associated with visceral pain. Indeed, our results match the findings of the occurrence of pain-induced anxiety-like behaviors following long-term (5–6 weeks) but not short-term (2–3 weeks) neuropathic pain (43).

An unexpected finding of this study was that, although GV and SAP reduced anxious phenotypes as revealed using the EPM, LDB and NSF tests, the effect of vagal deafferentation on the OF test failed to reach statistical significance. This could be related to the fact that although these 3 behavioral tests are commonly applied to assess anxiety-like behaviors in rodents, the differences in anxiogenic properties of the examination apparatus may contribute to varying levels of behavioral measures of anxiety (44). Considering these findings, we cannot exclude the possibility that other vagal-independent mechanisms may also contribute to anxiety-like behaviors in DSS-induced colitis mice. Although we highlight the importance of the neural mechanisms, it is also likely that hormonal or inflammatory mediators themselves may also be associated with an elevated anxiety state in DSS-induced colitis mice. Furthermore, it is noticeable that the effects of SAP–mediated vagal afferent ablation appear to be milder than those produced by surgical vagotomy. Given that surgical vagotomy eliminates all vagal sensory afferents and motor efferents and SAP specifically ablate ~80% of GI-derived vagal sensory afferent signaling (41), one possibility is that the complete and selective vagal deafferentation may cause different strength of action. Another possibility is that the vagal parasympathetic motor efferents may also play a role in DSS-induced anxiety-like behaviors. Interestingly, stimulation of parasympathetic vagal motor efferents has previously been demonstrated to elicit antiinflammatory responses in the gut via the release of acetylcholine to interact with α7 subunit of nicotinic receptor on macrophages (45), and impaired parasympathetic function has been shown to increase susceptibility to DSS-induced colitis (46, 47). Further investigation is needed to reveal the role of vagal motor efferents and sacral nerve in mediating top-down control of DSS-induced anxiety-like behaviors. Notably, in our study, while bilateral SDV also alleviates DSS-induced anxiety-like behaviors in the EPM test, it aggravated the severity of numerous DSS-induced colitic characteristics (Supplemental Figure 3) compared with sham- and GV-treated groups. A possible explanation may be that SDV eliminates both gastric and hepatic branches of the vagus nerve, and this elimination results in decreased antiinflammatory properties in the gut. Indeed, a recent study has emphasized the importance of the hepatic branch of the vagus nerve for sensing gut microenvironment to regulate the antiinflammatory vagal efferent pathway in DSS model (48). To our surprise, we found no significant effect of SDV on DSS-induced anxiety-like behaviors in the LDB test (Figure 3, F and G). The cause of these inconsistencies between the effect of SDV and GV on DSS-induced anxiety-like behaviors in the LDB test is unclear, but it may be related to the elimination of different branches of the vagus nerve, resulting in the activation of different physiological processes that may vary in their mode of action. Additional studies are needed to clarify this possibility.

Analog to vagal nerve stimulation, a novel method of sacral nerve stimulation, has been reported to reduce colonic inflammation in 2,4,6-trinitrobenzenesulfonic acid-induced rat colitis model by enhancing vagal activity mediated via the spinal afferents and vagal efferents (49, 50). Further investigation is needed to reveal the role of vagal motor efferents and sacral nerve in mediating top-down control of DSS-induced anxiety-like behaviors.

Our initial motivation to focus on the gastric vagal afferent signaling was based on previous study, which emphasized an important role of vagal afferents in the regulation of innate anxiety and learned fear (32). However, while vagal afferents convey sensory signals from the GI tract to the brain, spinal sensory afferents also innervate the colon and rectum (51). Thus, spinal afferents can also relay sensory information from the gut to the brain via the spinothalamic, spinoreticular, and spinomesencephalic tracts. It will be of interest for future studies to determine the role of spinal sensory afferents in the development of anxiety-like behaviors in DSS-induced colitis mice.

In line with the notion that the BLA is a crucial component of the neural circuit in orchestrating anxiety-like behaviors (20, 21), our findings revealed increased activity in the BLA in the EPM test in DSS-induced colitis mice. Several studies have already reported that the LC-NE system utilizes the BLA output to promote anxiety-like behaviors. For example, optogenetic activation of LC-BLA terminals evokes NE release, alters BLA neuronal activity, and increases anxiety-like behaviors (27). Conversely, chemogenetic silencing of the LC-BLA pathway relieves pain-induced anxiety (43). Accordingly, we found that chemogenetic inhibition of LC-NE projections to the BLA alleviates anxiety-like behaviors in DSS-induced colitis mice. This suggests that colitis-induced dysregulation of LC-NE inputs into the BLA may mediate the elevated anxiety state observed in DSS-treated mice. While the NTS is the first CNS site to receive GI-derived vagal sensory afferent inputs (17–19) and a recent study has described a direct projection from the NTS to the BLA (52), our retrograde tracing results revealed that the direct projection from the NTS to the BLA is relatively scarce. Our data confirm that NE neurons in the LC receive dense axonal inputs from the NTS (53, 54), which, in turn, send axonal projections to the BLA. Therefore, the LC may serve as a possible relay region connecting the NTS to the BLA. However, we could not exclude the possibility that, in addition to the LC, other brain regions may also relay signals from the NTS to the BLA to mediate anxiety-like behaviors in DSS-induced colitis mice. Interestingly, a previous study reported the presence of synaptic connections to the LC from the nucleus paragigantocellularis (PGi) neurons that receive direct inputs from the NTS (55). Further work could investigate whether the NTS-PGi-LC pathway also orchestrates anxiety-like behaviors.

Previous work has shown that subdiaphragmatic vagal deafferentation can reduce anxiety-like behaviors but increase auditory-cued fear conditioning responses in rats (31). Although our data show that GV and SAP consistently reduced anxiety-like behaviors in DSS-induced colitis mice, we found no evidence of their anxiolytic effects in mice before DSS administration. This suggests that the observed reduction of DSS colitis–induced anxiety-like behaviors in GV and SAP mice cannot be attributed to different anxious states in mice subjected to vagal deafferentation compared with sham-operated controls.

Some limitations of this study are worth noting. First, we need to use other behavioral assays independent of locomotor activity to assess anxiety state of DSS-induced colitis mice. Second, this study only used male mice for test subjects. Hence, we do not know at this stage whether gastric vagal afferent signaling similarly underlies the pathogenesis of anxiety-like behaviors in male and female mice with experimental colitis. Although a recent clinical study reported that patients with active IBD had higher degrees of illness perception and psychological comorbidities in females than in males (56), not all studies found significant differences between biological sexes in comorbid anxiety prevalence in patients with IBD (57–59). Nonetheless, a recent study has reported that both DSS-treated male and female mice showed increased anxiety-like behaviors as revealed using the OF and EPM tests (15). Third, it is not yet clear how local infection in the gut activates vagal afferents. Fourth, although clinical and histopathological features induced by DSS recapitulate those observed in human IBD, individual animal models cannot fully reflect the true nature of human diseases. We did not examine if the same neural mechanisms underlie comorbid anxiety-like behaviors in experimental CD models of IBD. Further research using existing models and/or the development of a novel model will contribute to our understanding of the mechanistic link between IBD and comorbid anxiety.

In summary, our results demonstrate that acute colitis leads to activation of vagal afferent signaling from the GI tract to regulate the LC-NE system in the BLA via the NTS, and this process then promotes the development of anxiety. We further identify the LC NE neurons as potential relay connecting the NTS to the BLA. Results from this study uncover an unidentified and important neural circuit mechanism underlying the comorbid anxiety during acute DSS-induced colitis and reveal that targeting LC-NE projections to the BLA may represent a therapeutic avenue for treating comorbid anxiety associated with IBD.

## Methods

### Animals.

Unless otherwise stated, adult C57BL/6 male mice (originally obtained from Charles River Laboratories and bred within the Laboratory Animal Center at National Cheng Kung University; aged 10–12 weeks at the start of experiments) were used. For targeted LC noradrenergic neurons experiments, we used male transgenic mice bearing a Cre recombinase expressed under the control of the dopamine-β-hydroxylase promoter (DBH-Cre mice; MMRRC strain name STOCK Tg[Dbh-cre]KH212Gsat/Mmcd, which were produced by Nathaniel Heintz [The Rockefeller University, New York, USA.] and Charles Gerfen [IH] laboratories using the BAC-transgenic strategy), aged 10–12 weeks at the start of experiments. Mice were socially housed in groups of 5 in humidity- and temperature-controlled (25°C ± 1°C) rooms on a consistent 12 -hour/12-hour light/dark cycle (lights on at 07:00 hours) with food and water provided ad libitum. All behavioral procedures were conducted during the light phase between 10:00 hours and 15:00 hours. Mice were acclimatized to the testing room for at least 1 hour before testing. Mice of same litter were randomly assigned after weaning, and all experiments were conducted with subjects of more than one litter. All efforts were made to minimize animal suffering and the number of animals used. The experimenters were blind to the treatment.

### Recombinant AAV vector production.

DNA plasmids encoding pAAV-human synapsin-1 (hSyn)-hM4D(Gi)-mCherry (Addgene, plasmid 50475), pAAV-Ubi-GFP (Addgene, plasmid 11155), and pAAV-Calcium-CaMKIIα-mCherry (Addgene, plasmid 114469) were obtained from Addgene. Plasmid DNA was amplified purified and collected using a standard plasmid maxiprep kit (Qiagen). The purified plasmids were mixed into CaCl_2_ solution with the DNA plasmid coding AAV-DJ and cotransfected into HEK293T cells using the calcium phosphate precipitation method. Transfected cells were harvested 72 hours after transfection, and the virus was purified using the AAV Purification Mega Kit (Cell Biolabs). Viral titers were 5 ***×*** 10^12^ particles/mL and stored in aliquots at –80°C until use.

### Stereotactic surgery and drug infusion.

Stereotactic surgery was carried out as described previously (60). Mice were anesthetized with a mixture of Zoletil (Zolazepam and tiletamine, 50 mg/kg) and Rompun (Xylazine, 5.8 mg/kg) in the stereotaxic frame (David Kopf Instruments) for the entire surgery, and body temperature was maintained with the heating pad. For virus or FG (4%) injection, a beveled injection pipette was inserted at the desired coordinate, and the preparation was slowly injected using a microprocessor-controlled injector over a period of 15 minutes. The pipette was left in place for an additional 5 minutes to allow for diffusion of the virus solution and then withdrawn. The locations of injection sites were based on the mouse brain atlas (60): nucleus of solitary tract (NTS; anteroposterior [AP], –7.4 mm; mediolateral [ML], ±0.3 mm; and dorsoventral [DV], –5.0 mm], LC (AP, –5.5 mm; ML, ±1.3 mm; and DV, –3.8 mm), and BLA (AP, –0.8 mm; ML, ±3.4 mm; and DV, –5.2 mm). Surgical wounds were closed with monofilament nylon suture and covered with a 2% xylocaine jelly.

### Colitis induction and characterization.

Colitis was induced by providing ad libitum DSS (36–50 kDa; MP Biomedicals) to mice in drinking water (2% wt/vol) for 8 days (D1–D8), followed by 1% DSS administration for 2 days (until D10). Control mice consumed tap water. Body weight and DAI were monitored daily after DSS administration. The DAI was determined daily by totaling the scores for weight loss (0: none, 1: 1%–5%, 2: 6%–10%, 3: 11%–18%, 4: >18%), stool consistency (0: normal, 1: soft but formed, 2: soft, 3: soft and wet, 4: diarrhea), and rectal bleeding (0: none, 2: bleeding, 4: gross bleeding), as described previously (29). Mice were euthanized for sample collection and histologic examination after completion of behavioral testing under isoflurane anesthesia. Samples of colon, small intestine, stomach, and spleen were collected, and their length and weight were measured. The histological severity of colitis was scored by H&E staining of colonic tissues, according to the scoring system described by Wirtz et al. (61). Histological damage was scored as follows: D: 0, none; 1, isolated focal epithelial damage; 2, erosions and ulcerations in mucosa layer; 3, extensive damage into the surface mucosal epithelium; and I: 0, infrequent; 1, increased, some neutrophils; 2, presence of inflammatory cell clusters in submucosal layer; 3, transmural infiltration of cells. The histological score (HS) is equal to the product of D and I (HS = D + I).

### Intestinal permeability analysis.

Intestinal permeability was assessed using the FITC-dextran (4 kDa; MilliporeSigma, FD4) permeability assay as described previously (62). Mice were food deprived for 4 hours, and FITC-dextran (60 mg/100 g body weight) was gavaged with soft plastic oral gavage needles. After 3 hours, blood was collected and centrifuged (2,000*g* for 15 minutes at 4°C) to separate the serum from the RBCs. Serum was diluted with an equal volume of PBS and tested in duplicate. The concentration of FITC-dextran was measured by a spectrophotometer with an excitation wavelength of 488 nm and emission wavelength of 520 nm.

### Vagotomy.

Surgery was conducted under sterile conditions and appropriate anesthesia. Mice were anesthetized with a mixture of Zoletil (Zolazepam and tiletamine, 50 mg/kg) and Rompun (Xylazine, 5.8 mg/kg). After sanitizing and removing fur of surgical area, a 2–3 cm laparotomy was made on the left side of abdomen, and then liver was retracted cranially to expose the esophagus and stomach. The anterior (right side) and posterior (left side; including the celiac branch) roots of vagal nerves were exposed along the esophagus from subdiaphragmatic region to gastric. After detaching the connective tissues around the lower esophageal sphincter, vagal nerves were transected with forceps gently, without damaging other organs. Bilateral GV was conducted by transecting both anterior and posterior branches of gastric vagal nerves without damaging the right gastric artery and the hepatic branch of anterior vagus. Right-side GV was performed by transecting the anterior branch of the gastric vagal nerve without damaging the hepatic branch and the posterior vagal branch. Bilateral SDV was conducted by transecting both anterior and posterior vagal branches, including the hepatic branch of the anterior vagus. Right-side SDV was conducted by transecting the anterior branch of vagus, including the hepatic branch, without lesioning of the posterior branch of vagus. Left-side vagotomy transected only the posterior branch of the vagus nerve without damaging the anterior branch of vagus. In sham surgeries, anterior and posterior branches of vagal nerves were exposed but were not transected. Following surgery, the incision was closed using monofilament nylon sutures and covered with a 2% xylocaine jelly. Advil (Ibuprofen, 0.8 mg/mL) was given in drinking water for 4 days after the surgery. The body weight of each mouse was monitored before surgery and for a period of 2 weeks after surgery.

### SAP NDG injection.

SAP NDG injection was carried out as described previously (19). Mice were anesthetized with i.p. injection of Zoletil (Zolazepam and tiletamine, 50 mg/kg) and Rompun (Xylazine, 5.8 mg/kg). The cervical ventral area was sanitized, and a midline incision was made along the neck. Submandibular glands and sternocleidomastoid muscle were retracted laterally, followed by retracting the omohyoid muscle to adequately expose the carotid sheath. The carotid sheath was incised and the vagus nerve can be identified running laterally to the common carotid artery. Tracing along the cervical vagus nerve to the bifurcation of common carotid artery, the connective tissues around the vagus nerves ganglia were carefully removed until the nodose ganglion was visible and accessible. SAP (250 ng/μL; Advanced Targeting Systems, IT-21) or Blnk (250 ng/μL; Advanced Targeting Systems, IT-31) were bilaterally or unilaterally injected to rostral (0.5 μL) and caudal (0.5 μL) parts of the nodose ganglion using a beveled injection pipette controlled by a microprocessor-controlled injector at the speed of 50 nL/sec. Following surgery, the incision was closed with monofilament nylon suture and covered with a 2% xylocaine jelly. Advil (Ibuprofen, 0.8 mg/mL) was given in drinking water for 4 days after the surgery.

### Chemogenetic manipulations.

For silencing of LC-NE projections to the BLA, AAV_DJ_-hSyn-hM4D(Gi)-mCherry was bilaterally injected into the LC (AP, –5.5 mm; ML, ±1.3 mm; DV, –3.7 to –3.8 mm) of DBH-Cre mice, which were bilaterally implanted with 27 gauge cannula guides aimed at the BLA (AP, –0.8 mm; ML, ±3.4 mm; and DV, –5.2 mm) to deliver CNO. Dummy cannulas were inserted into guide cannulas and secured to the skull with dental cement. Mice had a 3-week viral incubation period before starting the behavioral testing. Behavioral tests were performed 2 weeks after recovery from cannula implantation surgery. Mice were treated with DSS during the recovery period. CNO (1 mM in saline, 0.3 μL) was delivered locally through cannulas implanted bilaterally into the BLA 15 minutes before the behavioral test. Dose of CNO was selected on the basis of published studies (63). After behavioral testing, brains were dissected, and serial slices were imaged to verify correct viral expression.

### Anterograde and retrograde tracing.

To characterize the connection between the NTS, LC, and BLA, we used a combination of anterograde and retrograde tracing techniques in the same mouse. For anterograde tracing, AAV_DJ_-Ubi-GFP (0.2 μL) or AAV_DJ_-CaMKIIα-mCherry (0.2 μL) was injected into the NTS (AP, –7.4 mm; ML, 0.3 mm; and DV, –5.0 mm) of a single hemisphere of mice. After allowing 3 weeks for viral expression, a retrograde tracer FG (4%, 35 nL, Santa Cruz Biotechnology Inc., SC-358883) was injected into the BLA (AP, –0.8 mm; ML, 3.4 mm; DV, –5.2 mm) by using 1 μL Hamilton syringe. The syringe was slowly retracted after additional 5 min solution diffusion. The mice were perfused for tissue processing 11 days after FG injection.

### Rabies virus tracing.

Rabies virus-based monosynaptic retrograde tracing was carried out as previously described (64). A viral cocktail of AAV_5_-FLEX-TVA-GFP and AAV_5_-FLEX-RG (1:1 mixed, 0.5 μL) was injected into a single hemisphere of the LC (AP, –5.5 mm; ML, 1.3 mm; and DV, –3.7 to –3.8 mm) of DBH-Cre mice. After 3 weeks of incubation allowing the expression of helper viruses, EnvA-ΔG-mCherry (0.5 μL) rabies virus was injected at the same anatomic coordinate. After another 11 days to allow for monosynaptic retrograde labeling, mice were sacrificed and perfused, and slices were prepared for fluorescence microscopy. To preserve the location of surface membrane TVA receptor, we captured GFP signal with an antibody against GFP (1:2,000; abcam ab13970) in the BLA and amplified with a goat anti-Chicken secondary Alexa Fluor 488 antibody (1:2000; abcam ab150169). To preserve the mCherry signal, we used a chicken polyclonal antibody against mCherry (1:2000; abcam ab167453) and amplified with a goat anti–chicken secondary Alexa Fluor 594 antibody (1:2000; abcam ab150176).

### IHC.

Mice were transcardially perfused with 4 % PFA prepared in 0.1M PBS, PH 7.4. Brains were removed right after decapitation and fixed in 4 % PFA overnight at 4°C followed by at least 72 hours of incubation in 30 % sucrose before cryomicrotomy. Coronal slices (containing NTS, LC, and BLA) were sectioned into 40 μm and washed with PBS containing 0.4 % Triton X-100 (PBST); they were then incubated for 1 hour in solution containing 3% bovine serum albumin (BSA) in PBS for blocking. Slices were incubated in primary antibodies: anti–neuronal nuclei (NeuN; 1:2,000; MilliporeSigma, MAB377), anti–c-Fos (1:500; Cell Signaling Technology, 2250), anti-NET (1:2,000; Mab Technology, NET05-2), anti-GFP (1:2,000; abcam, ab13970), and anti-mCherry (1:2,000; abcam, ab167453) overnight at 4°C. After being washed with PBST, slices were incubated in secondary antibodies, Alexa Fluor 568 (Invitrogen, A11036), Alexa Fluor 488 (Invitrogen, A11001), goat anti–chicken Alexa Fluor 488 (1:2,000; abcam, ab150169), or goat anti–chicken Alexa Fluor 594 (1:2,000; abcam, ab150176) for 1 hour at room temperature (90 minutes for c-Fos staining). The sections were collected on separate gelatin-subbed glass slides, rinsed extensively in PBS, and mounted with ProLong Gold Antifade Reagent (Invitrogen). Nuclei were counterstained with DAPI (1:2,0000; Sigma-Aldrich, D9542). The slides were coverslipped and allowed to dry overnight. Fluorescence microscopic images of neurons were obtained using an Olympus FluoView FV3000 confocal laser scanning microscope (Olympus). For quantification of c-Fos immunopositivity, c-Fos^+^ neurons were determined only when cells were colocalized with NeuN staining and used visual-based semiquantitative estimation every sixth coronal section containing the NTS, LC, and BLA. All images were imported into NIH ImageJ software for analysis, and all the parameters used were kept consistent during capturing. The fluorescence intensity of c-Fos^+^ cells was at least 10-fold above the background.

### CCK-8 induced food intake suppression test.

To assess the completeness of total gastric vagotomy and SAP vagal deafferentation, a CCK-8–induced food intake suppression test was performed as previously described (65). Mice were deprived of food for 20 hours from 17:00 to 13:00 hours the next day. During the testing, CCK-8 (8 μg/kg; MilliporeSigma) was injected i.p. 15 minutes before the food-intake test. For the food-intake test, mice were allowed ad libitum feeding for 2 hours, and weight of food pellets was recorded before and after consumption. Food intake percentage was normalized to body weight.

### OF test.

The OF test was performed as previously described (66, 67). Each individual test mouse was placed in the center of the test apparatus and allowed to freely explore for 10 minutes under the dimmed illumination (10 lux). The OF apparatus consisted of a square ground area (42 cm ***×*** 42 cm) surrounded by a 42 cm–high wall set on a nonreflective white plastic base. The behavior of the animals was videotaped, tracked, and analyzed with EthoVision XT (Noldus) video tracking systems. Behavioral measurements included time spent in the center zone, total distance traveled, and number of rearing in the OF (68). The apparatus was cleaned with 70% ethanol between each trial. The center was defined as a square composed of 25% (21 cm ***×*** 21 cm) of the arena of the apparatus.

### LDB test.

The LDB test was performed as previously described (69). The apparatus consisted of a square (30 cm × 30 cm) divided into a small dark safe box (one-third, ~5 lux) and a large illuminated aversive box (two-thirds, ~250 lux) by a partition with door (3 cm × 3 cm). Each individual test mouse was placed in the dark box, and the time spent in the light box, the number of entries to the light box, and nose pokes within a 10-minute test session were videotaped and analyzed using EthoVision XT (Noldus) video tracking systems (70). The apparatus was cleaned with 70% ethanol between each trial.

### EPM test.

The EPM test was performed as previously described (67, 71). The apparatus was made from black Plexiglas with a light gray floor and consisted of 2 open (25 cm × 5 cm × 0.5 cm) and 2 closed arms (25 cm × 5 cm × 16 cm), which extended from a central platform (5 × 5 cm) at 50 cm from the floor. Each individual mouse was placed in the center area facing an open arm and allowed to freely explore the maze for 5 minutes. The apparatus was illuminated under dim light (10 lux). The behavior of the animals was videotaped, tracked, and analyzed with EthoVision XT (Noldus) video tracking systems. Behavioral measurements included time spent in the open and closed arms, number of rearing, and stretches toward the open arms (68). The apparatus was cleaned with 70% ethanol between each trial.

### von Frey test.

Hind paw withdrawal by the von Frey filament test was assessed by electronic von Frey hairs (part no. 2390; IITC Instruments). Mice were habituated to the testing room undisturbed for 30 minutes prior to the test. Eight trials were conducted for each side at an interval of 5 minutes with 0.8 mm rigid tip (SuperTips). Hind paw withdrawal was calculated by averaging 5 trials of tests after removing 3 tests that deviated the most from the medium, as previously described (72).

### Home cage activity (HCA).

HCA was determined as previously described (64). Each mouse was allowed to freely explore their home cage (accommodated for at least 3 days) for 10 minutes after 1 hour of resting. Total distance traveled was videotaped and analyzed using EthoVision XT (Noldus) video tracking systems.

### NSF test.

The NSF test was performed as described previously with minor modification (73). Mice were food deprived for 20 hours prior to the test. On the testing day, 3 familiar food pellets were placed on a plastic dish located in the middle of the novel context (42 cm ***×*** 42 cm ***×*** 42 cm). Each individual test mouse was placed in 1 corner of the context and was allowed to freely explore for 10 minutes. The latency of mouse to approach and eat the familiar food pellet was measured. The apparatus was cleaned with 70% ethanol between each trial.

### Statistics.

Sample sizes were based on previous work of a similar nature by our laboratory (47, 56) and determined with power analysis (a 2-tailed analysis with a significance set at α = 0.05 and power ≥ 80%) (G*Power software). No specific randomization method was used. Animals were randomly allocated into different experimental groups. All results are presented as mean ± SEM and analyzed by the GraphPad Prism 6 software. Normality of data distribution was verified using the D’Agostino & Pearson normality test, and Shapiro-Wilk test for data from Figure 5 as well as Supplemental Figures 3–5 and 8. For Gaussian distribution, 2-tailed unpaired Student’s *t* test was used to compare differences between 2 independent groups. For non-Gaussian distribution, the Mann-Whitney *U* test was used to compare differences between 2 independent groups. The difference between multiple groups was calculated by 1-way ANOVA (for Guassian-distributed data) or Kruskal-Wallis test (for non-Guassian–distributed data) followed by Dunn’s test or Tukey’s multiple-comparison post hoc analyses. The difference between multiple groups with 2 factors was calculated by 2-way repeated measures ANOVA or 2-way ANOVA followed by Dunn’s test or Tukey’s multiple-comparison post hoc analyses. For Figure 3, Sham-H_2_O, GV-Bi-H_2_O, and Sham DSS were calculated with GV-Bi-DSS, GV-R-DSS, and V-L-DSS, respectively, separately for 2-way ANOVA; Sham-H_2_O, SDV-Bi-H_2_O, and Sham-DSS were calculated with SDV-Bi-DSS, SDV-R-DSS ,and V-L-DSS, respectively, separately for 2-way ANOVA. For Figure 4, SAP-Blnk-H_2_O, CCK-SAP-H_2_O, and SAP-Blnk-DSS were calculated with CCK-SAP-DSS, CCK-SAP-R-DSS, and CCK-SAP-L-DSS, respectively, separately for 2-way ANOVA. Differences were considered significant at *P* < 0.05. The sample sizes and statistical test utilized for each figure are reported in the figure legends. The statistical results are described in Supplemental Table 1.

### Study approval.

All experiments complied with the *Guide for the Care and Use of Laboratory Animals* (National Academies Press, 2011), under protocols approved by the IACUC at National Cheng Kung University (IACUC approval no. 110188).

### Data and materials availability.

All data are available in the main text or the supplementary materials.

## Author contributions

CHC, TCT, YJW, and KSH designed research. CHC and TCT performed research and analyzed data. CHC and KSH wrote the paper.

## Supplementary Material

Supplemental data

## Version 1 05/18/2023

In-Press Preview

## Version 2 06/22/2023

Electronic publication
