# Supplementary material for: Gastric vagal afferent signaling to the basolateral amygdala mediates anxiety-like behaviors in experimental colitis mice
Source: JCI Insight. 2023 Jun 22;8(12):e161874. doi: 10.1172/jci.insight.161874 (PMC10371239; doi:10.1172/jci.insight.161874)
Supplement: Supplemental data [file jciinsight-8-161874-s093.pdf]

SUPPLEMENTARY MATERIALS

Gastric vagal afferent signaling to the basolateral amygdala mediates anxiety-like behaviors in experimental colitis mice

Chin-Hao Chen,[1] Tsung-Chih Tsai,[1] Yi-Jen Wu,[2,3] and Kuei-Sen Hsu[1,4,*]

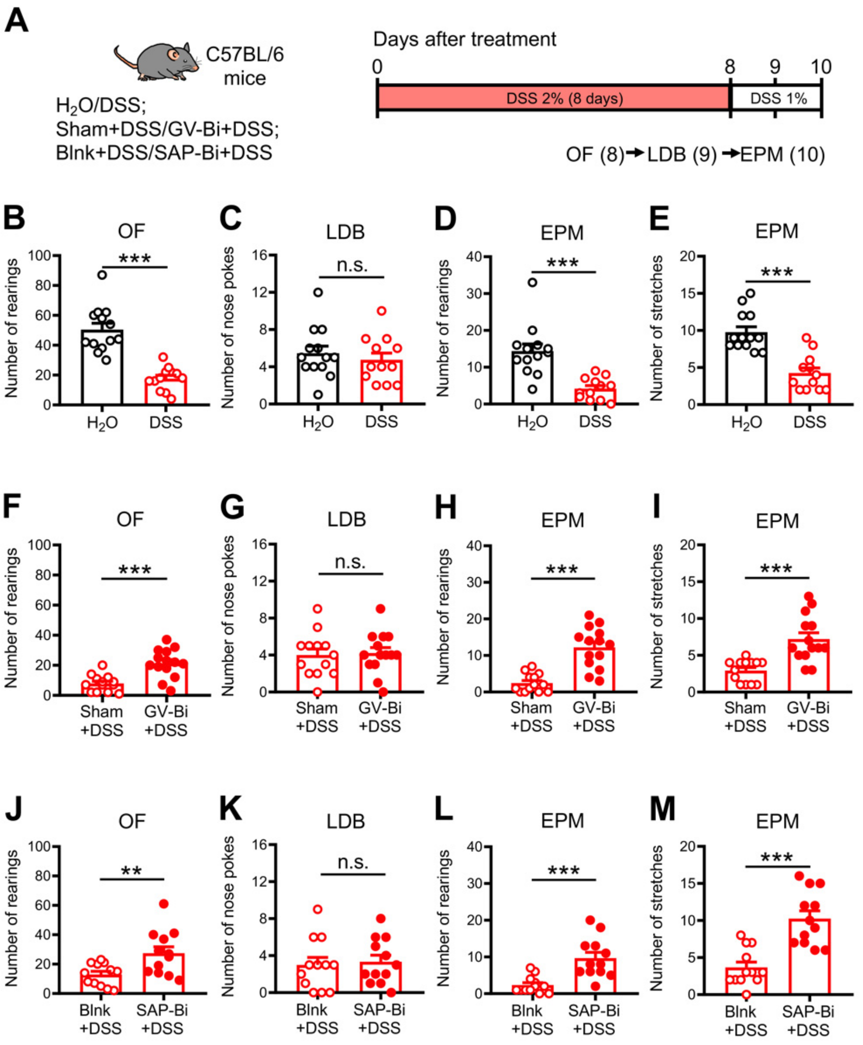

Supplemental Figure 1. Gastric vagotomy and targeted ablation of vagal afferent neurons by CCK-SAP injections rescue anxiety-related risk assessment behavioral deficits in DSS-induced colitis mice. (A) Schematic illustration of the experimental timeline. Wild type, Sham, Bilateral gastric vagotomy (GV-Bi), SAP-Blank (Blnk) and CCK-SAP (SAP-Bi) mice received 2% DSS in drinking water for 8 days followed by a maintenance dose of 1% for two days. All groups were subjected to behavioral tests on D9 and D10. **(B-E)** Comparing the effect of H_2O and DSS treatment on the number of rearing in the open field (OF) test **(B)**, the number of nose pokes in the light dark box (LDB) test **(C)** and the number of rearing and number of stretches in the elevated plus maze (EPM) test **(D and E)** (H_2O: n = 13, DSS: n = 12). **(F-I)** Comparing the effect of sham + DSS and GV-Bi + DSS on the number of rearing in the OF test **(F)**, the number of nose pokes in LDB test **(G)** and the number of rearing and number of stretches in the EPM test **(H and I)** (sham-DSS: n = 13, GV-Bi-DSS: n = 14). **(J-M)** Comparing the effect of Blnk + DSS and SAP-Bi + DSS on the number of rearing in the OF test **(J)**, the number of nose pokes in the LDB test **(K)** and the number of rearing and number of stretches in the EPM test **(L and M)** (n = 12 in each group). Data represent the mean ± SEM. ** $P < 0.01$, *** $P < 0.001$and n.s., not significant by two-tailed Student's *t*-test and Mann-Whitney *U* test **(D)**.

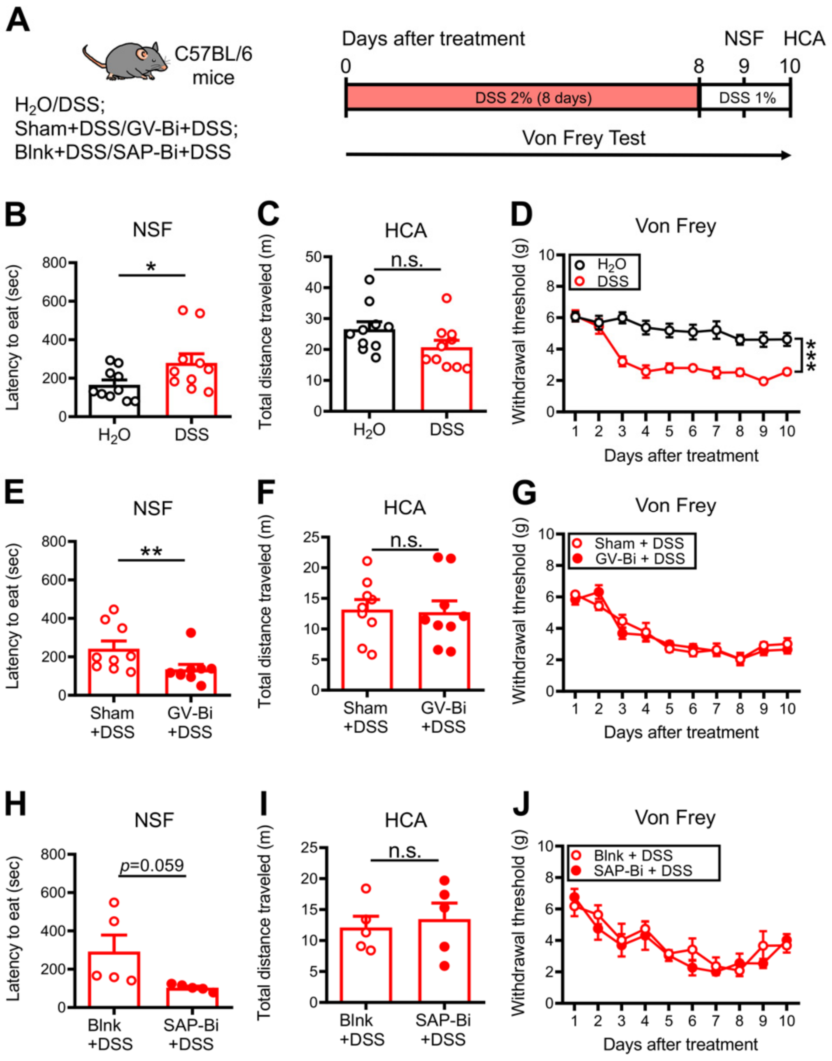

Supplemental Figure 2. Gastric vagotomy and targeted ablation of vagal afferent neurons by CCK-SAP injection reduce anxiety level of DSS-induced colitis mice without affecting pain sensitivity and locomotor activity. (A) Schematic illustration of the experimental timeline. Wild type, Sham, Bilateral gastric vagotomy (GV-Bi), SAP-Blank (Blnk) and CCK-SAP (SAP-Bi) mice received 2% DSS in drinking water for 8 days followed by a maintenance dose of 1% for two days. All groups were subjected to behavioral tests on D9 and D10. **(B-D)** Comparing the effect of H_2O and DSS treatment on **(B)** novelty suppressed food intake (NSF) test, **(C)** home cage activity test (HCA) and **(D)** von Frey test (n = 10 in each group). **(E-G)** Comparing the effect of sham + DSS and GV-Bi + DSS on **(E)** novelty suppressed food intake (NSF) test, **(F)** home cage activity test (HCA) and **(G)** von Frey test (n = 9 in each group). **(H-J)** Comparing the effect of Blnk + DSS and SAP-Bi + DSS on **(H)** novelty suppressed food intake (NSF) test, **(I)** home cage activity test (HCA) and **(J)** von Frey test (n = 5 in each group). Data represent the mean ± SEM. * $P < 0.05$, ** $P < 0.01$, *** $P < 0.001$and n.s., not significant by two-way RM ANOVA **(D, G, J)**, two-tailed Student's *t*-test **(B, C, E, H, I)** and Mann-Whitney *U* test **(F)**.

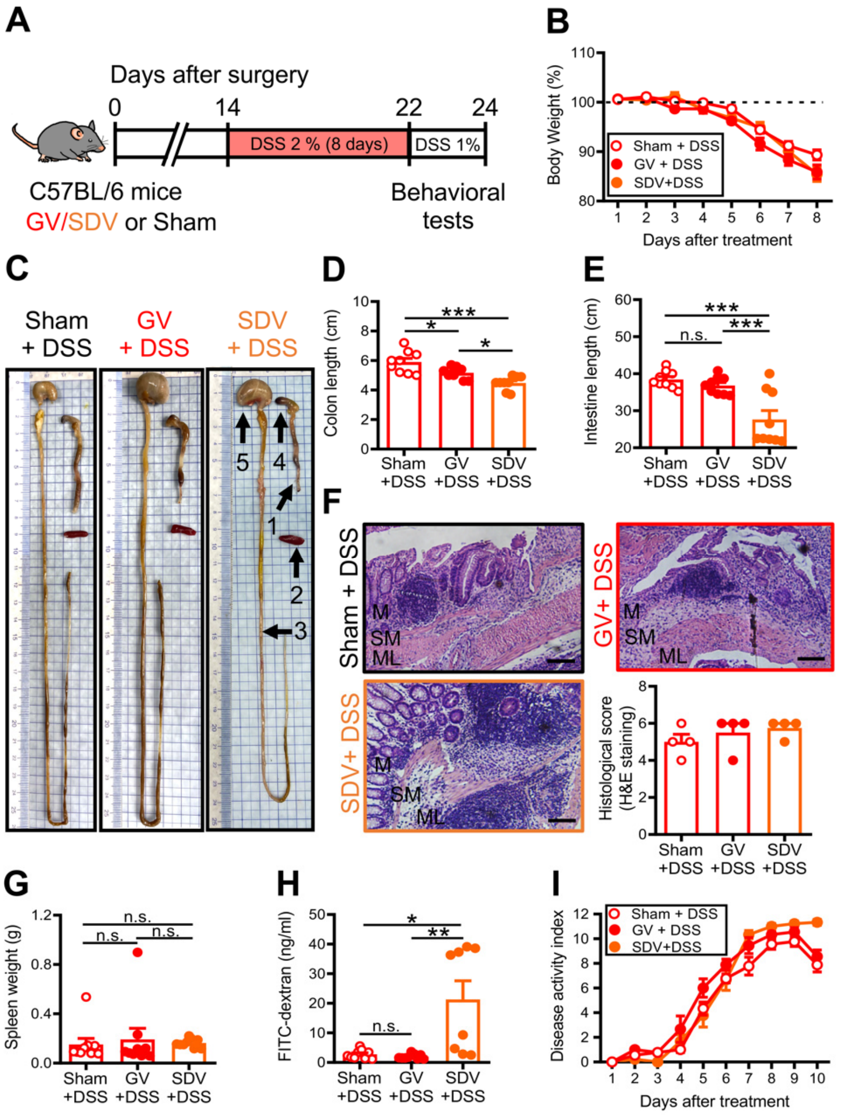

Supplemental Figure 3. The effect of gastric and subdiaphragmatic vagotomy on DSS-induced colitis phenotypes. **(A)** Schematic of the experimental timeline. Two weeks after surgery, mice received 2% DSS in drinking water for 8 days followed by 1% for two days. All groups were subjected to behavioral tests on D22, D23 and D24 before sacrifice. **(B)** Body weights recorded during the experimental period (sham-DSS: n = 9, GV-DSS: n = 9, SDV-DSS: n=9). **(C)** Gross morphology images of sham-DSS, GV-DSS and SDV-DSS group. Arrows indicate: (1) colon; (2) spleen; (3) small intestine; (4) cecum; and (5) stomach. **(D, E, G)** Quantification of **(D)** colon length; **(E)** small intestine length and **(G)** spleen weight on D25 in sham-DSS, GV-DSS and SDV-DSS groups (n = 9 in each group). **(F)** H&E staining and histological score of colonic sections were assessed on D25 in sham-DSS, GV-DSS and SDV-DSS groups (n = 4 in each group). M: mucosa layer, SM: submucosa layer, and ML: muscular layer. Scale bar, 200 µm. **(H)** Quantification of FITC-dextran permeability on D25 in sham-DSS, GV-DSS and SDV-DSS groups (n = 9 in each group). **(J)** DAI score was used to evaluate the severity of DSS-induced colitis recorded from D14 to D24 (n = 9 in each group). DAI score was calculated total score (body weight + stool consistency + rectal bleeding). Data represent the mean ± SEM. * $P < 0.05$, ** $P < 0.01$, *** $P < 0.001$and n.s., not significant by one-way ANOVA with Tukey's multiple comparison test **(D, E)** and Kruskal-Wallis test with Tukey's multiple comparison test **(H)**.

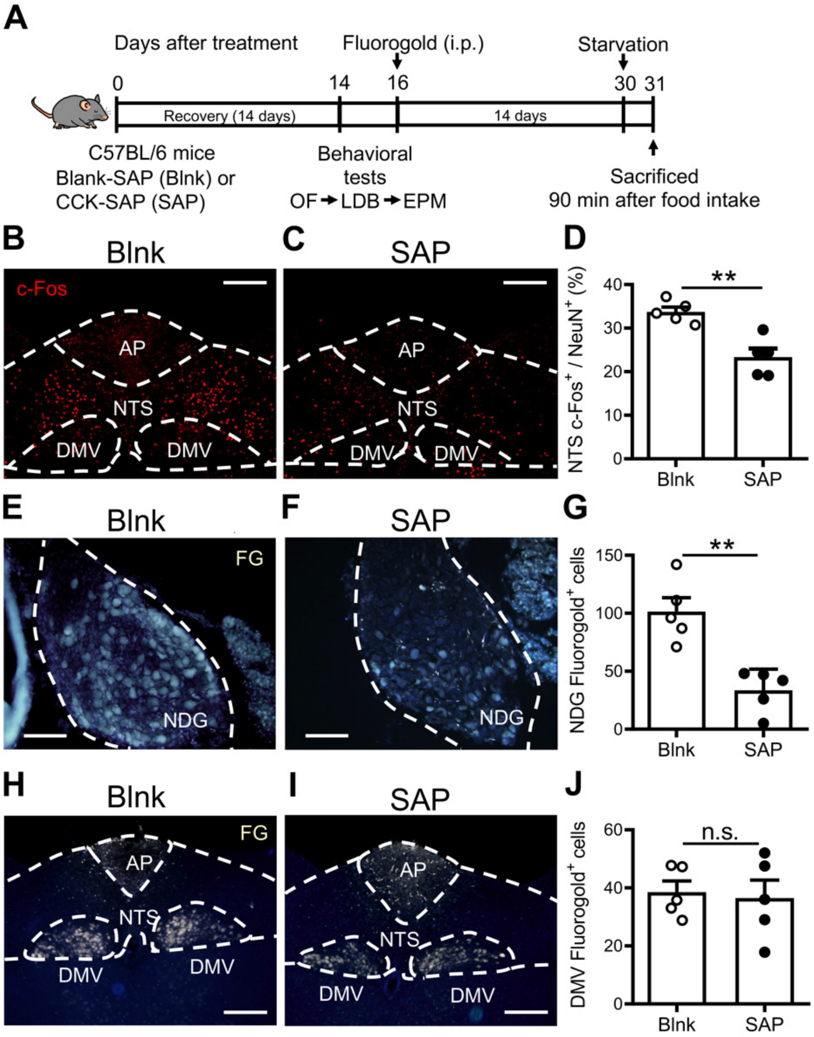

Supplemental Figure 4. Targeted ablation of vagal afferent neurons by CCK-SAP injection. (A) Schematic illustration of the experimental timeline. Sixteen days after mice received nodal injection of Blank-SAP (Blnk) or CCK-SAP (SAP), Fluorogold (FG, 10 mg/kg) was injected intraperitoneally (i.p.) after behavioral tests. Two weeks after FG injection, mice were sacrificed 90 min after food intake. Mice were food deprived for 20 h prior to food intake. **(B)** and **(C)** Representative images and **(D)** quantitative analysis of c-Fos-labeled cells in Blnk and SAP mice 90 min after food intake within the nucleus tractus solitarius (NTS, n = 5 in each group). Scale bar, 200 μm. **(E)** and **(F)** Representative images and **(G)** quantitative analysis of FG-labeled cells in nodose ganglia (NDG) 15 days after intraperitoneal injection of FG (n = 5 in each group). Scale bar, 100 μm. **(H)** and **(I)** Representative images and **(J)** quantitative analysis of FG-labeled cells in dorsal motor nucleus of the vagus (DMV) 15 days after intraperitoneal injection of FG (DMV, n = 5 in each group). Scale bar, 200 μm. Data represent the mean ± SEM. $^{**}P < 0.01$ and n.s., not significant by two-tailed Student's *t*-test.

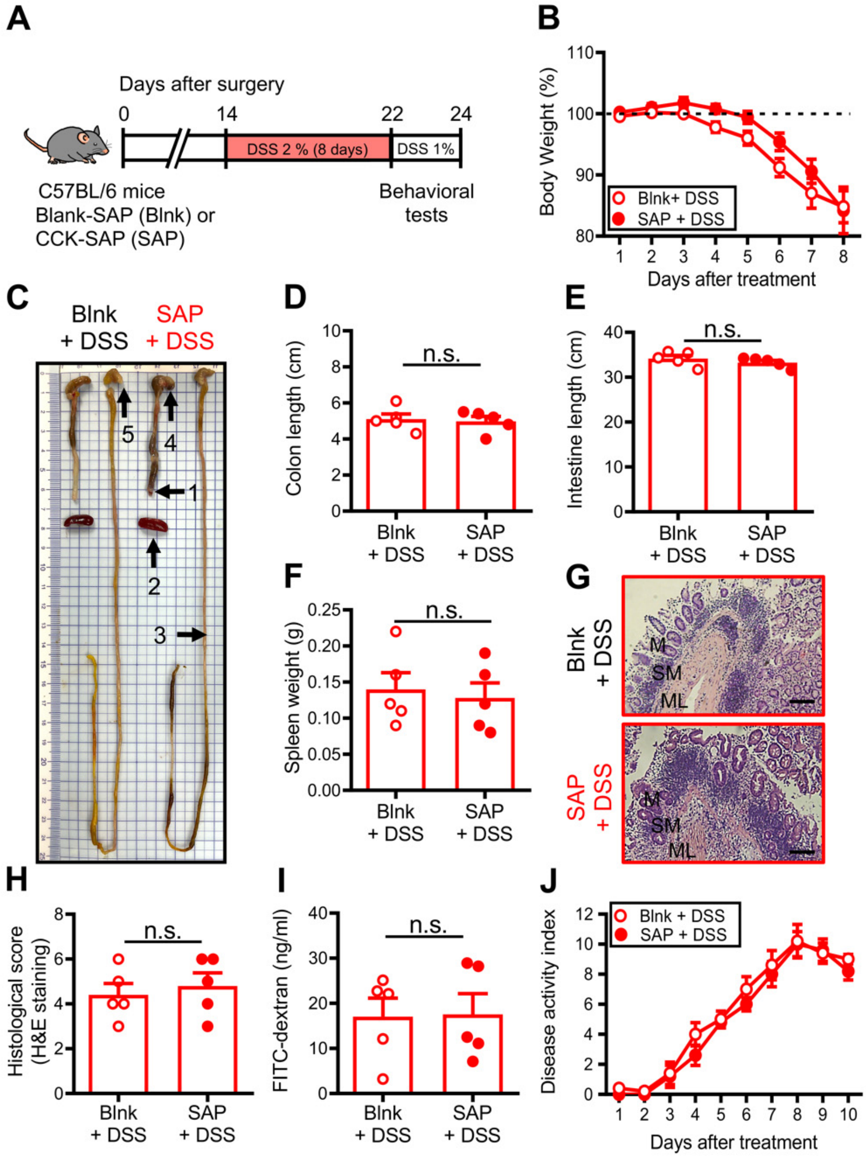

Supplemental Figure 5. The effect of CCK-SAP nodose injection on DSS-induced colitis phenotypes. (A) Schematic of the experimental timeline. Two weeks after surgery, mice received 2% DSS in drinking water for 8 days followed by a maintenance dose of 1% for two days. All groups were subjected to behavioral tests on D22, D23 and D24 before sacrifice. **(B)** Body weights recorded during the experimental period of Blank-SAP + DSS (Blnk + DSS) and CCK-SAP + DSS mice (SAP + DSS) (Blnk + DSS: n = 5, SAP + DSS: n = 5). **(C)** Gross morphology images of Blnk + DSS and SAP + DSS groups. Arrows indicate: (1) colon; (2) spleen; (3) small intestine; (4) cecum; and (5) stomach. **(D-F)** Quantification of **(D)** colon length; **(E)** small intestine length and **(F)** spleen weight on D25 in Blnk + DSS and SAP + DSS groups (n = 5 in each group). **(G)** H&E staining and **(H)** histological score of colonic sections were assessed on D25 in Blnk + DSS and SAP + DSS groups (n = 5 in each group). M: mucosa layer, SM: submucosa layer, and ML: muscular layer. Scale bar, 200 μm. **(I)** Quantification of FITC-dextran permeability on D25 in Blnk + DSS and SAP + DSS groups (n = 5 in each group). **(J)** DAI score was used to evaluate the severity of DSS-induced colitis recorded from D14 to D24 (n = 5 in each group). DAI score was calculated total score (body weight + stool consistency + rectal bleeding). Data represent the mean ± SEM.n.s., not significant by two-tailed unpaired Student's *t*-test **(D, E, F, H, I)**.

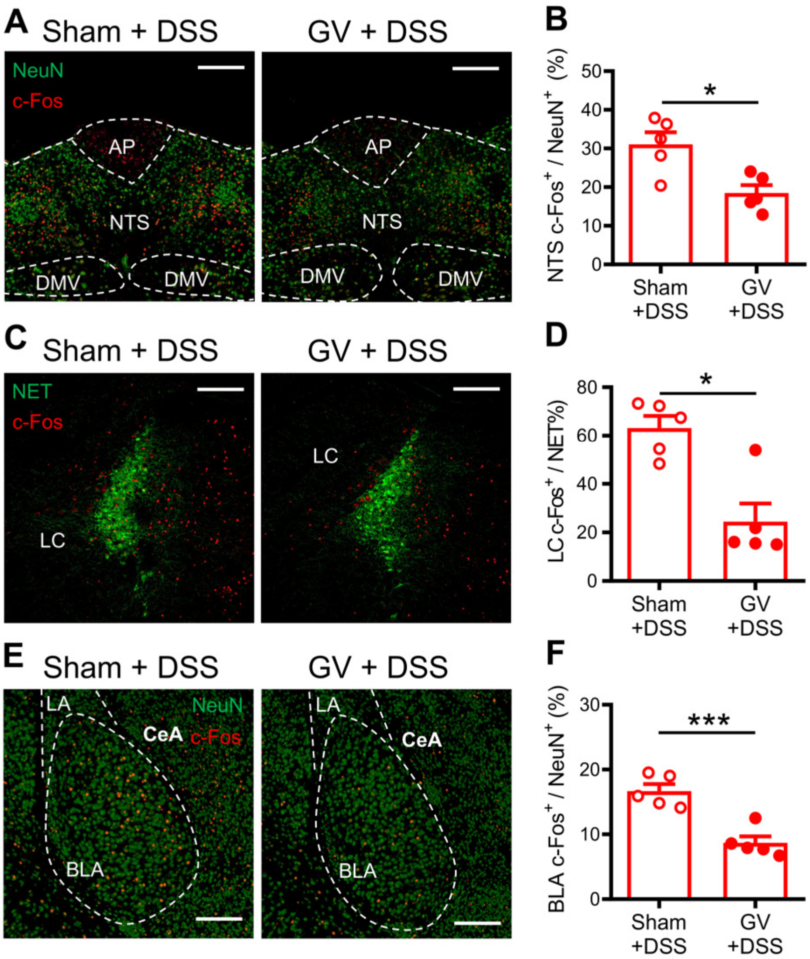

Supplemental Figure 6. Gastric vagotomy reduces c-Fos expression in multiple brain regions after the elevated plus maze test in DSS-induced colitis mice. Representative images and quantitative analysis of c-Fos-labeled cells in Sham + DSS and GV + DSS mice 90 min after the EPM test within the nucleus tractus solitarius (NTS, n = 5 in each group) **(A and B)**, locus coeruleus (LC, n = 5 in each group) **(C and D)** and basolateral amygdala (BLA, n = 5 in each group) **(E and F)**. Scale bar, 200 µm. AP, area postrema; DMV, dorsal motor nucleus of the vagus; LA, lateral amygdala; CeA, central nucleus of the amygdala. Data represent the mean ± SEM. *$P < 0.05$ and *** $P < 0.001$ by two-tailed unpaired Student's *t*-test **(B and F)**, Mann-Whitney *U* test **(D)**.

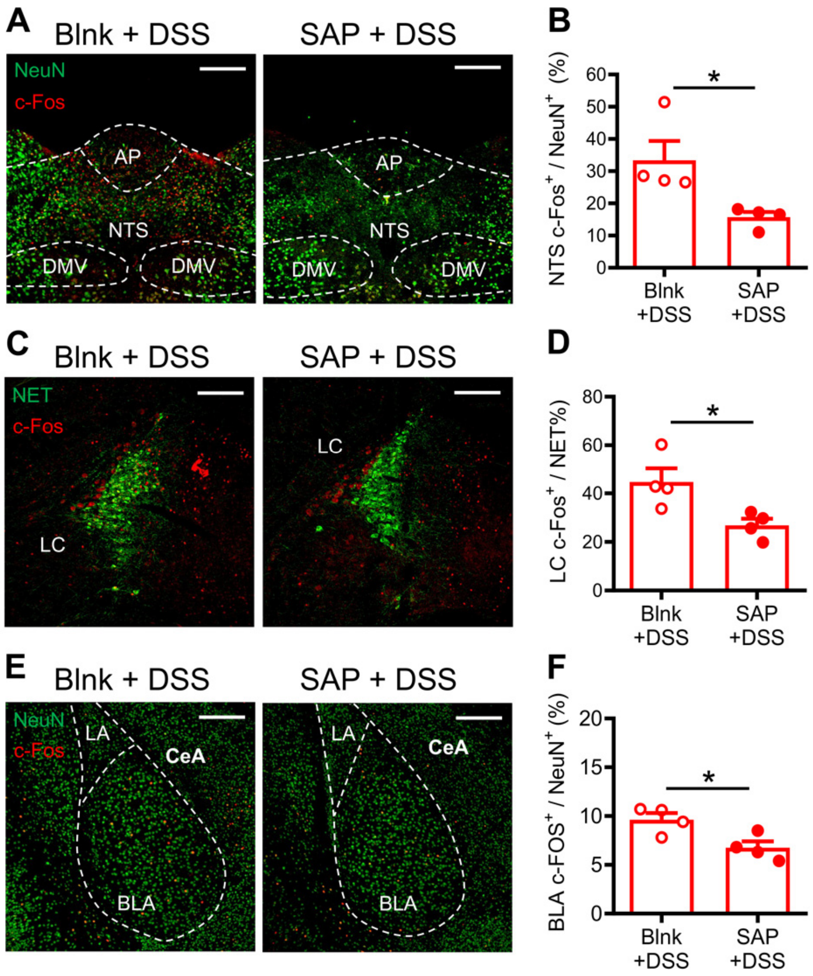

Supplemental Figure 7. Targeted ablation of vagal afferent neurons by CCK-SAP injection reduces c-Fos expression in multiple brain regions after the elevated plus maze test in DSS-induced colitis mice. Representative images and quantitative analysis of c-Fos-labeled cells in Blank-SAP-DSS (Blnk + DSS) and CCK-SAP + DSS (SAP + DSS) mice 90 min after the EPM test within the nucleus tractus solitarius (NTS, n = 4 in each group) **(A and B)**, locus coeruleus (LC, n = 4 in each group) **(C and D)** and basolateral amygdala (BLA, n = 4 in each group) **(E and F)**. Scale bar, 200 μm. AP, area postrema; DMV, dorsal motor nucleus of the vagus; LA, lateral amygdala; CeA, central nucleus of the amygdala. Data represent the mean ± SEM. *$P < 0.05$ by Mann-Whitney *U* test **(B)**, two-tailed unpaired Student's *t*-test **(D and F)**.

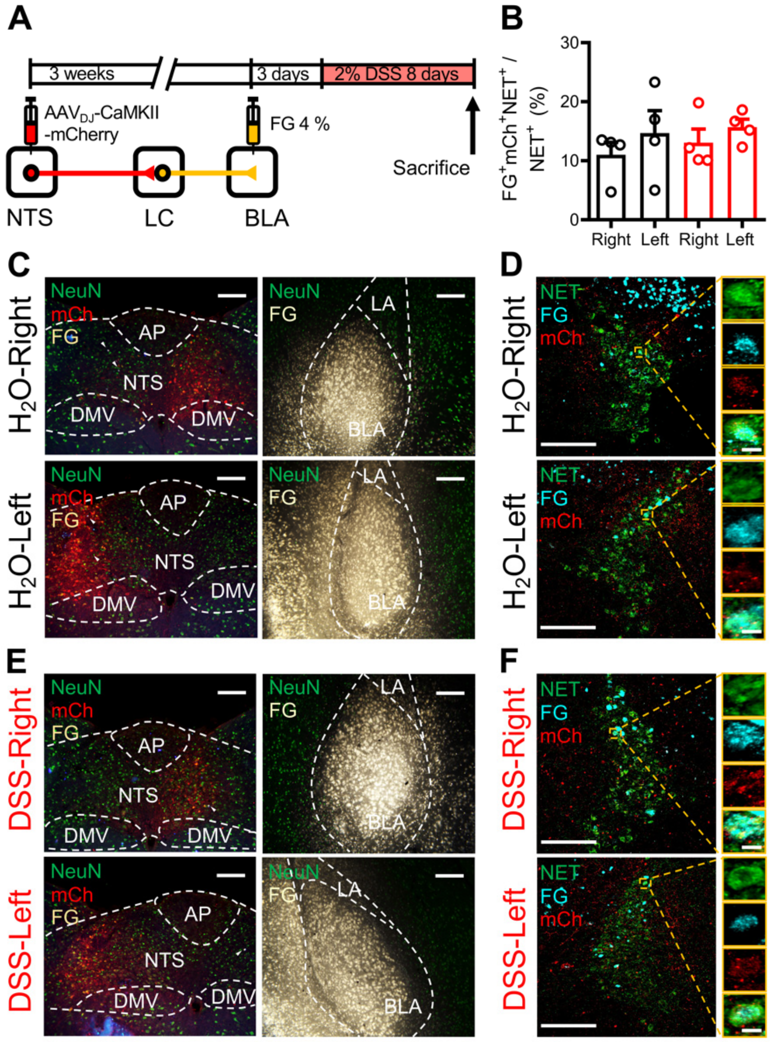

Supplemental Figure 8. The effect of DSS treatment on unilateral NTS innervated LC-NET neuronal projection to the BLA. (A) Schematic of the experimental timeline. Mice received AAV_{DJ}-CaMKIIa-mCherry injection into right (or left) NTS and incubated for three weeks followed by 4 % FluoroGold (FG) injection into right (or left) BLA. Three days after FG injection, mice received 2 % DSS in drinking water for 8 days and sacrificed on the last day of DSS treatment. **(B)** Bar graph comparing the amount of LC neurons (NET^{+}) as the relay connecting the NTS to the BLA in both sides of H_2O- and DSS-treated groups (n = 4 in each group). **(C and E)** Representative image showing tracer injection sites of H_2O- and DSS-treated groups. CaMKIIa (red, mCh) signals in the NTS and FG signals in the BLA. Arrow heads represent FG signals in NTS from BLA. Scale bar, 200 µm. **(D and F)** Representative image showing FG-labeled LC neurons were immunopositive for NET and CaMKII signals in H_2O- and DSS-treated groups. Scale bar, 200 µm. Augmented figures (Right side) showing NET neurons in rectangle area. Scale bar, 10 µm. AP, area postrema; DMV, dorsal motor nucleus of the vagus; LA, lateral amygdala. Data represent the mean ± SEM. For statistical analysis, two-way ANOVA for **(B)**.

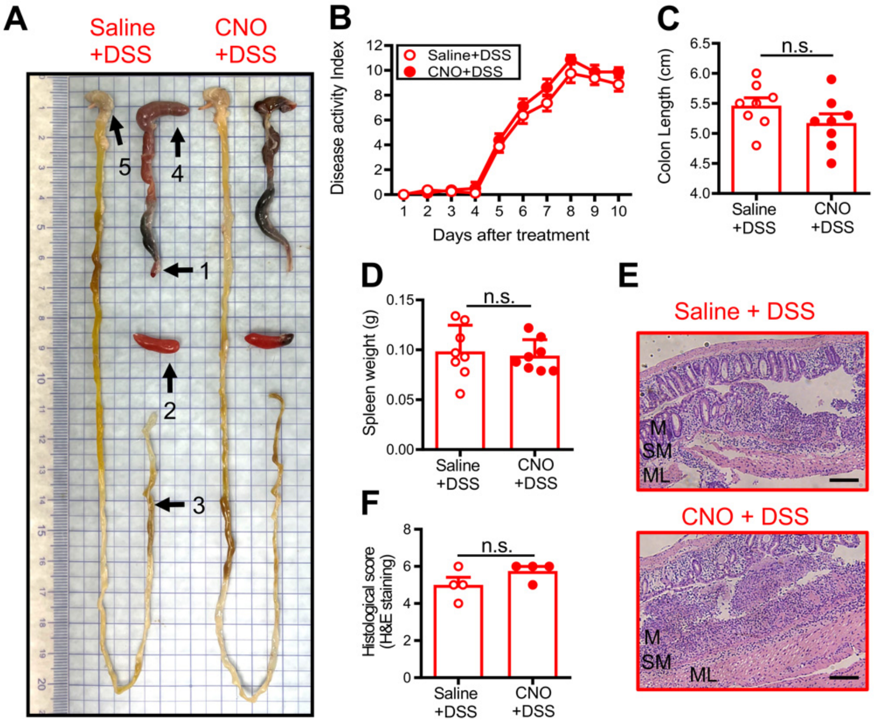

Supplemental Figure 9. The effect of CNO and saline on DSS-induced colitis phenotypes. (A) Gross morphology images of Saline + DSS and CNO + DSS groups. Arrows indicate: (1) colon; (2) spleen; (3) small intestine; (4) cecum; and (5) stomach. **(B)** DAI score was used to evaluate the severity of DSS-induced colitis recorded from D1 to D10 (n = 8 in each group). DAI score was calculated total score (body weight + stool consistency + rectal bleeding). **(C)** Quantification of colon length in Saline + DSS and CNO + DSS groups (n = 8 in each group). **(D)** Quantification of spleen weight in Saline + DSS and CNO + DSS groups (n = 8 in each group). **(E)** H&E staining and **(F)** histological score of colonic sections were assessed in Saline + DSS and CNO + DSS groups (n = 4 in each group). M: mucosa layer, SM: submucosa layer, and ML: muscular layer. Scale bar, 200 μm. Data represent the mean ± SEM. n.s., not significant by two-tailed unpaired Student's *t*-test **(C and D)**, Mann-Whitney *U* test **(F)**.

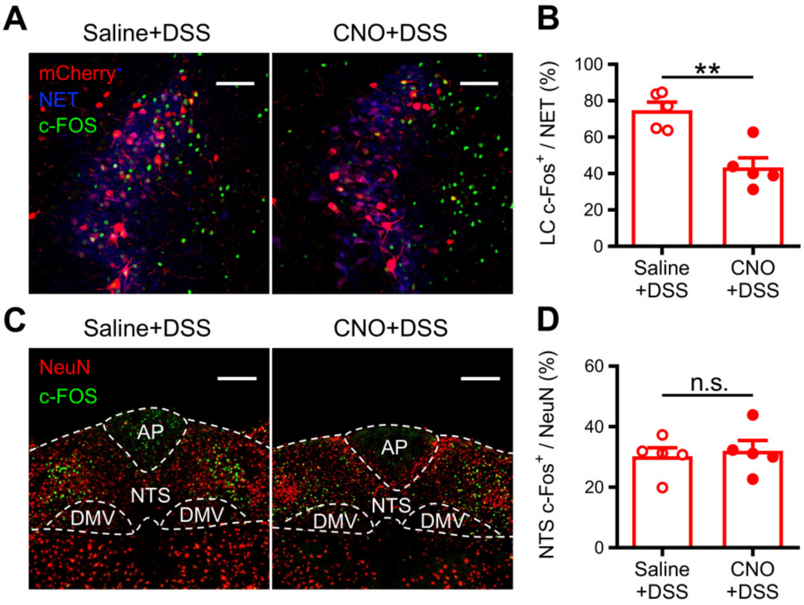

Supplemental Figure 10. Chemogenetic manipulation reduces c-Fos expression in locus coeruleus but not nucleus tractus solitarius after the elevated plus maze test in DSS-induced colitis mice. Representative images and quantitative analysis of c-Fos-labeled cells in Saline + DSS and CNO + DSS mice 90 min after the EPM test within the locus coeruleus (LC, n = 4 in each group) **(A and B)** and nucleus tractus solitarius (NTS, n = 4 in each group) **(C and D)**. Scale bar, 200 μm. AP, area postrema; DMV, dorsal motor nucleus of the vagus. Data represent the mean ± SEM. ** $P < 0.01$ and ns., not significant by two-tailed unpaired Student's *t*-test **(B and D)**.

Table S1. Details of statistical data

Figure No.	Parameter	Test specifications	Genotype	Treatment	Sex	Number of individuals	Mean ± SEM	Statistical test	Fixed effects F(DFn,DFd)	*p* = value
Fig.1B	Body weight	H2O	WT	-	Male	10	-	Two-way RM ANOVA	Time x DSS $F_{(7,126)}$ = 42.39	*p* < 0.001
		DSS	WT	-	Male	10				
**Figure**	**Parameter**	**Test specifications**	**Genotype**	**Treatment**	**Sex**	**Number of individuals**	**Mean ± SEM**	**Statistical test**	**t-distribution t, df**	***p* = value**
Fig.1D	Colon length (cm)	H2O	WT	-	Male	10	7.67 ± 0.11	Mann-Whitney U test	-	*p* < 0.001
		DSS	WT	-	Male	10	5.20 ± 0.27			
Fig.1E	Intestine length (cm)	H2O	WT	-	Male	10	42.92 ± 0.69	Two-tailed unpaired Student's t-test	$t_{(18)}$ = 2.96	*p* = 0.009
		DSS	WT	-	Male	10	38.11 ± 1.48			
Fig.1F	Spleen weight (g)	H2O	WT	-	Male	10	0.06 ± 0.01	Two-tailed unpaired Student's t-test	$t_{(18)}$ = 7.19	*p* < 0.001
		DSS	WT	-	Male	10	0.12 ± 0.01			
Fig.1H	Histological score	H2O	WT	-	Male	6	0.50 ± 0.22	Mann-Whitney U test	-	*p* = 0.002
		DSS	WT	-	Male	6	4.33 ± 0.67			
Fig.1I	FITC-Dextran (ng/ml)	H2O	WT	-	Male	10	0.64 ± 0.15	Two-tailed unpaired Student's t-test	$t_{(18)}$ = 2.89	*p* = 0.001
		DSS	WT	-	Male	10	1.45 ± 0.24			
**Figure**	**Parameter**	**Test specifications**	**Genotype**	**Treatment**	**Sex**	**Number of individuals**	**Mean ± SEM**	**Statistical test**	**Fixed effects F(DFn,DFd)**	***p* = value**
Fig.1J	Disease activity index	H2O	WT	-	Male	10	-	Two-way RM ANOVA	Time x DSS $F_{(9,162)}$ = 71.55	*p* < 0.001
		DSS	WT	-	Male	10				

Figure	Parameter		Test specifications	Genotype	Treatment	Sex	Number of individuals	Median (Q1,Q3)	Statistical test	t-distribution t, df	*p* = value
Fig.2B	OFT	total distance (m)	H2O	WT	-	Male	13	44.10 (36.08, 48.96)	Mann-Whitney U test	-	*p* < 0.001
			DSS	WT	-	Male	12	22.35 (18.71, 24.38)			
Fig.2C		time in center (sec)	H2O	WT	-	Male	13	14.00 (10.50, 16.50)	Two-tailed unpaired Student's t-test	$t_{(23)}$ = 5.45	*p* < 0.001
			DSS	WT	-	Male	12	5.50 (2.00, 7.00)			
Fig.2D	LDB	duration (sec)	H2O	WT	-	Male	13	36.00 (25.00, 50.00)	Two-tailed unpaired Student's t-test	$t_{(23)}$ = 2.30	*p* = 0.031
			DSS	WT	-	Male	12	18.50 (9.00, 40.75)			
Fig.2E		entries	H2O	WT	-	Male	13	13.00 (9.50, 17.50)	Two-tailed unpaired Student's t-test	$t_{(23)}$ = 2.03	*p* = 0.054
			DSS	WT	-	Male	12	8.00 (3.00, 11.50)			
Fig.2G	EPM	total distance (m)	H2O	WT	-	Male	13	16.12 (8.84, 18.64)	Mann-Whitney U test	-	*p* = 0.016
			DSS	WT	-	Male	12	7.59 (5.28, 11.11)			
Fig.2H		open arms (sec)	H2O	WT	-	Male	13	15.20 (4.40, 22.80)	Mann-Whitney U test	-	*p* = 0.035
			DSS	WT	-	Male	12	1.35 (0.53, 6.40)			
Fig.2I		closed arms (sec)	H2O	WT	-	Male	13	60.80 (47.95, 90.35)	Two-tailed unpaired Student's t-test	$t_{(23)}$ = 2.40	*p* = 0.025
			DSS	WT	-	Male	12	89.60 (74.78, 96.73)			

Figure	Parameter	Test specifications	Genotype	Treatment	Sex	Number of individuals	Mean ± SEM	Statistical test	Dunn's test	*p* = value
Fig.3C	Food intake (g)	Sham	WT	-	Male	23	0.57 ± 0.02	Kruskal-Wallis test (*p* < 0.001)	-	-
		GV-Bi	WT	-	Male	24	0.82 ± 0.04		Sham vs. GV	*p* < 0.001
		SDV	WT	-	Male	23	1.03 ± 0.07		Sham vs. SDV	*p* < 0.001
		GV-R	WT	-	Male	13	0.73 ± 0.04		Sham vs. GV-R	*p* = 0.033
		SDV-R	WT	-	Male	12	0.83 ± 0.03		Sham vs. SDV-R	*p* < 0.001
		V-L	WT	-	Male	13	0.84 ± 0.04		Sham vs. V-L	*p* < 0.001

Figure	Parameter		Test specifications	Genotype	Treatment	Sex	Number of individuals	Median (Q1,Q3)	Statistical test	t-distribution t, df	*p* = value
Fig.3D	OFT	total distance (m)	Sham	WT	H_2O	Male	10	50.35 (39.53, 55.98)	Two-tailed unpaired Student's t-test	$t_{(21)}$ = 8.61	*p* < 0.001
			Sham	WT	DSS	Male	13	17.59 (12.91, 23.51)			

Figure	Parameter		Test specifications	Genotype	Treatment	Sex	Number of individuals	Median (Q1,Q3)	Statistical test	Fixed effects F(DFn,DFd)	*p* = value
Fig.3D	OFT	total distance (m)	Sham	WT	H_2O	Male	10	50.35 (39.53, 55.98)	Two-way ANOVA	DSS x Vagotomy $F_{(1,43)}$ = 1.91	*p* = 0.175
			GV-Bi	WT	H_2O	Male	10	33.50 (28.03, 39.70)			
			Sham	WT	DSS	Male	13	17.59 (12.91, 23.51)			
			**GV-Bi**	**WT**	**DSS**	**Male**	**14**	**25.38 (16.88, 34.07)**			
			Sham	WT	H_2O	Male	10	50.35 (39.53, 55.98)	Two-way ANOVA	DSS x Vagotomy $F_{(1,42)}$ = 0.16	*p* = 0.688
			GV-Bi	WT	H_2O	Male	10	33.50 (28.03, 39.70)			
			Sham	WT	DSS	Male	13	17.59 (12.91, 23.51)			
			**GV-R**	**WT**	**DSS**	**Male**	**13**	**16.20 (11.15, 19.25)**			
			Sham	WT	H_2O	Male	10	50.35 (39.53, 55.98)	Two-way ANOVA	DSS x Vagotomy $F_{(1,42)}$ = 0.38	*p* = 0.542
			GV-Bi	WT	H_2O	Male	10	33.50 (28.03, 39.70)			
			Sham	WT	DSS	Male	13	17.59 (12.91, 23.51)			
			**V-L**	**WT**	**DSS**	**Male**	**13**	**9.90 (7.75, 21.15)**			
Fig.3D	OFT	total distance (m)	Sham	WT	H_2O	Male	10	50.35 (39.53, 55.98)	Two-way ANOVA	DSS x Vagotomy $F_{(1,43)}$ = 0.67	*p* = 0.417
			SDV-Bi	WT	H_2O	Male	10	42.80 (35.95, 55.75)			
			Sham	WT	DSS	Male	13	17.59 (12.91, 23.51)			
			**SDV-Bi**	**WT**	**DSS**	**Male**	**13**	**14.60 (8.95, 17.10)**			
			Sham	WT	H_2O	Male	10	50.35 (39.53, 55.98)	Two-way ANOVA	DSS x Vagotomy $F_{(1,41)}$ = 2.42	*p* = 0.127
			SDV-Bi	WT	H_2O	Male	10	42.80 (35.95, 55.75)			
			Sham	WT	DSS	Male	13	17.59 (12.91, 23.51)			
			**SDV-R**	**WT**	**DSS**	**Male**	**12**	**17.30 (11.18, 23.18)**			
			Sham	WT	H_2O	Male	10	50.35 (39.53, 55.98)	Two-way ANOVA	DSS x Vagotomy $F_{(1,42)}$ = 0.92	*p* = 0.342
			SDV-Bi	WT	H_2O	Male	10	42.80 (35.95, 55.75)			
			Sham	WT	DSS	Male	13	17.59 (12.91, 23.51)			
			**V-L**	**WT**	**DSS**	**Male**	**13**	**9.90 (7.75, 21.15)**			

Figure	Parameter		Test specifications	Genotype	Treatment	Sex	Number of individuals	Median (Q1,Q3)	Statistical test	t-distribution t, df	*p* = value
Fig.3E	OFT	time in center (sec)	Sham	WT	H_2O	Male	10	8.45 (7.25, 14.45)	Two-tailed unpaired Student's t-test	$t_{(21)}$ = 4.27	*p* < 0.001
			Sham	WT	DSS	Male	13	2.00 (1.00, 5.00)			

Figure	Parameter		Test specifications	Genotype	Treatment	Sex	Number of individuals	Median (Q1,Q3)	Statistical test	Fixed effects F(DFn,DFd)	*p* = value
Fig.3E	OFT	time in center (sec)	Sham	WT	H_2O	Male	10	8.45 (7.25, 14.45)	Two-way ANOVA	DSS x Vagotomy $F_{(1,43)}$ = 1.84	*p* = 0.182
			GV-Bi	WT	H_2O	Male	10	8.90 (7.45, 10.78)			
			Sham	WT	DSS	Male	13	2.00 (1.00, 5.00)			
			**GV-Bi**	**WT**	**DSS**	**Male**	**14**	**2.50 (1.70, 6.25)**			
			Sham	WT	H_2O	Male	10	8.45 (7.25, 14.45)	Two-way ANOVA	DSS x Vagotomy $F_{(1,42)}$ = 1.57	*p* = 0.218
			GV-Bi	WT	H_2O	Male	10	8.90 (7.45, 10.78)			
			Sham	WT	DSS	Male	13	2.00 (1.00, 5.00)			
			**GV-R**	**WT**	**DSS**	**Male**	**13**	**2.80 (1.05, 6.10)**			
			Sham	WT	H_2O	Male	10	8.45 (7.25, 14.45)	Two-way ANOVA	DSS x Vagotomy $F_{(1,42)}$ = 0.73	*p* = 0.398
			GV-Bi	WT	H_2O	Male	10	8.90 (7.45, 10.78)			
			Sham	WT	DSS	Male	13	2.00 (1.00, 5.00)			
			**V-L**	**WT**	**DSS**	**Male**	**13**	**0.80 (0.20, 4.65)**			
Fig.3E	OFT	time in center (sec)	Sham	WT	H_2O	Male	10	8.45 (7.25, 14.45)	Two-way ANOVA	DSS x Vagotomy $F_{(1,43)}$ = 3.86	*p* = 0.056
			SDV-Bi	WT	H_2O	Male	10	9.05 (5.28, 10.80)			
			Sham	WT	DSS	Male	13	2.00 (1.00, 5.00)			
			**SDV-Bi**	**WT**	**DSS**	**Male**	**13**	**5.40 (1.95, 17.40)**			
			Sham	WT	H_2O	Male	10	8.45 (7.25, 14.45)	Two-way ANOVA	DSS x Vagotomy $F_{(1,41)}$ = 4.26	*p* = 0.045
			SDV-Bi	WT	H_2O	Male	10	9.05 (5.28, 10.80)			
			Sham	WT	DSS	Male	13	2.00 (1.00, 5.00)			
			**SDV-R**	**WT**	**DSS**	**Male**	**12**	**4.70 (1.75, 6.90)**			

Figure	Parameter		Test specifications	Genotype	Treatment	Sex	Number of individuals	Median (Q1,Q3)	Statistical test	Fixed effects F(DFn,DFd)	*p* = value
Fig.3E	OFT	time in center (sec)	Sham	WT	H_2O	Male	10	8.45 (7.25, 14.45)	Two-way ANOVA	DSS x Vagotomy $F_{(1,42)} = 1.41$	$p = 0.241$
			SDV-Bi	WT	H_2O	Male	10	9.05 (5.28, 10.80)			
			Sham	WT	DSS	Male	13	2.00 (1.00, 5.00)			
			**V-L**	**WT**	**DSS**	**Male**	**13**	**0.80 (0.20, 4.65)**			

Figure	Parameter		Test specifications	Genotype	Treatment	Sex	Number of individuals	Median (Q1,Q3)	Statistical test	Compared Groups	*p* = value
Fig.3E	OFT	time in center (sec)							Two-way ANOVA Tukey's Multiple Comparisons test	H_2O Sham vs. H_2O SDV-Bi	$p = 0.160$
			Sham	WT	H_2O	Male	10	8.45 (7.25, 14.45)		H_2O Sham vs. DSS Sham	$p < 0.001$
			SDV-Bi	WT	H_2O	Male	10	9.05 (5.28, 10.80)		H_2O Sham vs. DSS SDV-R	$p < 0.001$
			Sham	WT	DSS	Male	13	2.00 (1.00, 5.00)		H_2O SDV-Bi vs. DSS Sham	$p = 0.003$
			**SDV-R**	**WT**	**DSS**	**Male**	**12**	**4.70 (1.75, 6.90)**		H_2O SDV-Bi vs. DSS SDV-R	$p = 0.022$
										DSS Sham vs. DSS SDV-R	$p = 0.891$

Figure	Parameter		Test specifications	Genotype	Treatment	Sex	Number of individuals	Median (Q1,Q3)	Statistical test	t-distribution t, df	*p* = value
Fig.3F	LDB	duration (sec)	Sham	WT	H_2O	Male	10	32.50 (20.50, 43.25)	Two-tailed unpaired Student's t-test	$t_{(21)} = 3.89$	$p < 0.001$
			Sham	WT	DSS	Male	13	10.00 (6.00, 24.00)			

Figure	Parameter		Test specifications	Genotype	Treatment	Sex	Number of individuals	Median (Q1,Q3)	Statistical test	Fixed effects F(DFn,DFd)	*p* = value
Fig.3F	LDB	duration (sec)	Sham	WT	H_2O	Male	10	32.50 (20.50, 43.25)	Two-way ANOVA	DSS x Vagotomy $F_{(1,43)} = 9.78$	$p = 0.003$
			GV-Bi	WT	H_2O	Male	10	26.00 (21.25, 41.00)			
			Sham	WT	DSS	Male	13	10.00 (6.00, 24.00)			
			**GV-Bi**	**WT**	**DSS**	**Male**	**14**	**46.85 (20.58, 62.10)**			
			Sham	WT	H_2O	Male	10	32.50 (20.50, 43.25)	Two-way ANOVA	DSS x Vagotomy $F_{(1,42)} = 2.22$	$p = 0.144$
			GV-Bi	WT	H_2O	Male	10	26.00 (21.25, 41.00)			
			Sham	WT	DSS	Male	13	10.00 (6.00, 24.00)			
			**GV-R**	**WT**	**DSS**	**Male**	**13**	**15.80 (7.10, 52.25)**			
			Sham	WT	H_2O	Male	10	32.50 (20.50, 43.25)	Two-way ANOVA	DSS x Vagotomy $F_{(1,42)} = 0.87$	$p = 0.356$
			GV-Bi	WT	H_2O	Male	10	26.00 (21.25, 41.00)			
			Sham	WT	DSS	Male	13	10.00 (6.00, 24.00)			
			**V-L**	**WT**	**DSS**	**Male**	**13**	**14.30 (9.10, 25.10)**			
Fig.3F	LDB	duration (sec)	Sham	WT	H_2O	Male	10	32.50 (20.50, 43.25)	Two-way ANOVA	DSS x Vagotomy $F_{(1,43)} = 6.07$	$p = 0.018$
			SDV-Bi	WT	H_2O	Male	10	29.50 (19.00, 38.25)			
			Sham	WT	DSS	Male	13	10.00 (6.00, 24.00)			
			**SDV-Bi**	**WT**	**DSS**	**Male**	**13**	**21.20 (14.25, 35.85)**			
			Sham	WT	H_2O	Male	10	32.50 (20.50, 43.25)	Two-way ANOVA	DSS x Vagotomy $F_{(1,41)} = 3.32$	$p = 0.076$
			SDV-Bi	WT	H_2O	Male	10	29.50 (19.00, 38.25)			
			Sham	WT	DSS	Male	13	10.00 (6.00, 24.00)			
			**SDV-R**	**WT**	**DSS**	**Male**	**12**	**21.75 (8.43, 31.95)**			
			Sham	WT	H_2O	Male	10	32.50 (20.50, 43.25)	Two-way ANOVA	DSS x Vagotomy $F_{(1,42)} = 2.08$	$p = 0.157$
			SDV-Bi	WT	H_2O	Male	10	29.50 (19.00, 38.25)			
			Sham	WT	DSS	Male	13	10.00 (6.00, 24.00)			
			**V-L**	**WT**	**DSS**	**Male**	**13**	**14.30 (9.10, 25.10)**			

Figure	Parameter		Test specifications	Genotype	Treatment	Sex	Number of individuals	Median (Q1,Q3)	Statistical test	Compared Groups	*p* = value
Fig.3F	LDB	duration (sec)							Two-way ANOVA Tukey's Multiple Comparisons test	H_2O Sham vs. H_2O GV-Bi	$p = 0.984$
			Sham	WT	H_2O	Male	10	32.50 (20.50, 43.25)		H_2O Sham vs. DSS Sham	$p = 0.022$
			GV-Bi	WT	H_2O	Male	10	26.00 (21.25, 41.00)		H_2O Sham vs. DSS GV-Bi	$p = 0.737$
			Sham	WT	DSS	Male	13	10.00 (6.00, 24.00)		H_2O GV-Bi vs. DSS Sham	$p = 0.056$
			**GV-Bi**	**WT**	**DSS**	**Male**	**14**	**46.85 (20.58, 62.10)**		H_2O GV-Bi vs. DSS GV-Bi	$p = 0.502$
										DSS Sham vs. DSS GV-Bi	$p < 0.001$

Figure	Parameter		Test specifications	Genotype	Treatment	Sex	Number of individuals	Median (Q1,Q3)	Statistical test	Compared Groups	*p* = value
Fig.3F	LDB	duration (sec)							Two-way ANOVA Tukey's Multiple Comparisons test	H_2O Sham vs. H_2O SDV-Bi	*p* = 0.592
			Sham	WT	H_2O	Male	10	32.50 (20.50, 43.25)		H_2O Sham vs. DSS Sham	*p* < 0.001
			SDV-Bi	WT	H_2O	Male	10	29.50 (19.00, 38.25)		H_2O Sham vs. DSS SDV-Bi	*p* = 0.146
			Sham	WT	DSS	Male	13	10.00 (6.00, 24.00)		H_2O SDV-Bi vs. DSS Sham	*p* = 0.028
			**SDV-Bi**	**WT**	**DSS**	**Male**	**13**	**21.20 (14.25, 35.85)**		H_2O SDV-Bi vs. DSS SDV-Bi	*p* = 0.847
										DSS Sham vs. DSS SDV-Bi	*p* = 0.112

Figure	Parameter		Test specifications	Genotype	Treatment	Sex	Number of individuals	Median (Q1,Q3)	Statistical test	t-distribution t, df	*p* = value
Fig.3G	LDB	entries	Sham	WT	H_2O	Male	10	12.50 (8.75, 15.25)	Mann-Whitney U test	-	*p* < 0.001
			Sham	WT	DSS	Male	13	3.00 (2.00, 5.50)			

Figure	Parameter		Test specifications	Genotype	Treatment	Sex	Number of individuals	Median (Q1,Q3)	Statistical test	Fixed effects F(DFn,DFd)	*p* = value
Fig.3G	LDB	entries	Sham	WT	H_2O	Male	10	12.50 (8.75, 15.25)	Two-way ANOVA	DSS x Vagotomy $F_{(1,43)} = 4.80$	*p* = 0.034
			GV-Bi	WT	H_2O	Male	10	10.50 (7.75, 15.00)			
			Sham	WT	DSS	Male	13	3.00 (2.00, 5.50)			
			**GV-Bi**	**WT**	**DSS**	**Male**	**14**	**6.00 (4.75, 12.00)**			
			Sham	WT	H_2O	Male	10	12.50 (8.75, 15.25)	Two-way ANOVA	DSS x Vagotomy $F_{(1,42)} = 0.07$	*p* = 0.790
			GV-Bi	WT	H_2O	Male	10	10.50 (7.75, 15.00)			
			Sham	WT	DSS	Male	13	3.00 (2.00, 5.50)			
			**GV-R**	**WT**	**DSS**	**Male**	**13**	**3.00 (1.00, 5.00)**			
			Sham	WT	H_2O	Male	10	12.50 (8.75, 15.25)	Two-way ANOVA	DSS x Vagotomy $F_{(1,42)} = 0.19$	*p* = 0.664
			GV-Bi	WT	H_2O	Male	10	10.50 (7.75, 15.00)			
			Sham	WT	DSS	Male	13	3.00 (2.00, 5.50)			
			**V-L**	**WT**	**DSS**	**Male**	**13**	**3.00 (2.00, 3.50)**			
Fig.3G	LDB	entries	Sham	WT	H_2O	Male	10	12.50 (8.75, 15.25)	Two-way ANOVA	DSS x Vagotomy $F_{(1,43)} = 0.05$	*p* = 0.817
			SDV-Bi	WT	H_2O	Male	10	13.50 (10.50, 20.00)			
			Sham	WT	DSS	Male	13	3.00 (2.00, 5.50)			
			**SDV-Bi**	**WT**	**DSS**	**Male**	**13**	**21.20 (14.25, 35.85)**			
			Sham	WT	H_2O	Male	10	12.50 (8.75, 15.25)	Two-way ANOVA	DSS x Vagotomy $F_{(1,41)} = 0.18$	*p* = 0.675
			SDV-Bi	WT	H_2O	Male	10	13.50 (10.50, 20.00)			
			Sham	WT	DSS	Male	13	3.00 (2.00, 5.50)			
			**SDV-R**	**WT**	**DSS**	**Male**	**12**	**3.00 (2.25, 6.75)**			
			Sham	WT	H_2O	Male	10	12.50 (8.75, 15.25)	Two-way ANOVA	DSS x Vagotomy $F_{(1,42)} = 0.02$	*p* = 0.888
			SDV-Bi	WT	H_2O	Male	10	13.50 (10.50, 20.00)			
			Sham	WT	DSS	Male	13	3.00 (2.00, 5.50)			
			**V-L**	**WT**	**DSS**	**Male**	**13**	**3.00 (2.00, 3.50)**			

Figure	Parameter		Test specifications	Genotype	Treatment	Sex	Number of individuals	Median (Q1,Q3)	Statistical test	Compared Groups	*p* = value
Fig.3G	LDB	entries							Two-way ANOVA Tukey's Multiple Comparisons test	H_2O Sham vs. H_2O GV-Bi	*p* = 0.956
			Sham	WT	H_2O	Male	10	12.50 (8.75, 15.25)		H_2O Sham vs. DSS Sham	*p* < 0.001
			GV-Bi	WT	H_2O	Male	10	10.50 (7.75, 15.00)		H_2O Sham vs. DSS GV-Bi	*p* = 0.216
			Sham	WT	DSS	Male	13	3.00 (2.00, 5.50)		H_2O GV-Bi vs. DSS Sham	*p* = 0.002
			**GV-Bi**	**WT**	**DSS**	**Male**	**14**	**6.00 (4.75, 12.00)**		H_2O GV-Bi vs. DSS GV-Bi	*p* = 0.497
										DSS Sham vs. DSS GV-Bi	*p* = 0.040

Figure	Parameter		Test specifications	Genotype	Treatment	Sex	Number of individuals	Median (Q1,Q3)	Statistical test	t-distribution t, df	*p* = value
Fig.3H	EPM	total distance (m)	Sham	WT	H_2O	Male	10	14.75 (12.28, 17.15)	Mann-Whitney U test	-	*p* < 0.001
			Sham	WT	DSS	Male	13	4.55 (3.50, 5.48)			

Figure	Parameter		Test specifications	Genotype	Treatment	Sex	Number of individuals	Median (Q1,Q3)	Statistical test	Fixed effects F(DFn,DFd)	*p* = value
Fig.3H	EPM	total distance (m)	Sham	WT	H_2O	Male	10	14.75 (12.28, 17.15)	Two-way ANOVA	DSS x Vagotomy $F_{(1,43)} = 4.81$	$p = 0.034$
			GV-Bi	WT	H_2O	Male	10	11.00 (7.75, 12.43)			
			Sham	WT	DSS	Male	13	4.55 (3.50, 5.48)			
			**GV-Bi**	**WT**	**DSS**	**Male**	**14**	**10.08 (5.55, 14.20)**			
			Sham	WT	H_2O	Male	10	14.75 (12.28, 17.15)	Two-way ANOVA	DSS x Vagotomy $F_{(1,42)} = 0.07$	$p = 0.797$
			GV-Bi	WT	H_2O	Male	10	11.00 (7.75, 12.43)			
			Sham	WT	DSS	Male	13	4.55 (3.50, 5.48)			
			**GV-R**	**WT**	**DSS**	**Male**	**13**	**4.60 (3.55, 8.90)**			
			Sham	WT	H_2O	Male	10	14.75 (12.28, 17.15)	Two-way ANOVA	DSS x Vagotomy $F_{(1,42)} = 0.44$	$p = 0.512$
			GV-Bi	WT	H_2O	Male	10	11.00 (7.75, 12.43)			
			Sham	WT	DSS	Male	13	4.55 (3.50, 5.48)			
			**V-L**	**WT**	**DSS**	**Male**	**13**	**6.40 (4.40, 9.85)**			
Fig.3H	EPM	total distance (m)	Sham	WT	H_2O	Male	10	14.75 (12.28, 17.15)	Two-way ANOVA	DSS x Vagotomy $F_{(1,43)} = 10.46$	$p = 0.002$
			SDV-Bi	WT	H_2O	Male	10	16.00 (12.78, 19.15)			
			Sham	WT	DSS	Male	13	4.55 (3.50, 5.48)			
			**SDV-Bi**	**WT**	**DSS**	**Male**	**13**	**4.90 (3.35, 7.10)**			
			Sham	WT	H_2O	Male	10	14.75 (12.28, 17.15)	Two-way ANOVA	DSS x Vagotomy $F_{(1,41)} = 9.72$	$p = 0.003$
			SDV-Bi	WT	H_2O	Male	10	16.00 (12.78, 19.15)			
			Sham	WT	DSS	Male	13	4.55 (3.50, 5.48)			
			**SDV-R**	**WT**	**DSS**	**Male**	**12**	**4.55 (3.93, 6.15)**			
			Sham	WT	H_2O	Male	10	14.75 (12.28, 17.15)	Two-way ANOVA	DSS x Vagotomy $F_{(1,42)} = 16.21$	$p < 0.001$
			SDV-Bi	WT	H_2O	Male	10	16.00 (12.78, 19.15)			
			Sham	WT	DSS	Male	13	4.55 (3.50, 5.48)			
			**V-L**	**WT**	**DSS**	**Male**	**13**	**6.40 (4.40, 9.85)**			

Figure	Parameter		Test specifications	Genotype	Treatment	Sex	Number of individuals	Median (Q1,Q3)	Statistical test	Compared Groups	*p* = value
Fig.3H	EPM	total distance (m)							Two-way ANOVA Tukey's Multiple Comparisons test	H_2O Sham vs. H_2O GV-Bi	$p = 0.964$
			Sham	WT	H_2O	Male	10	12.50 (8.75, 15.25)		H_2O Sham vs. DSS Sham	$p < 0.001$
			GV-Bi	WT	H_2O	Male	10	10.50 (7.75, 15.00)		H_2O Sham vs. DSS GV-Bi	$p = 0.028$
			Sham	WT	DSS	Male	13	3.00 (2.00, 5.50)		H_2O GV-Bi vs. DSS Sham	$p < 0.001$
			**GV-Bi**	**WT**	**DSS**	**Male**	**14**	**6.00 (4.75, 12.00)**		H_2O GV-Bi vs. DSS GV-Bi	$p = 0.007$
										DSS Sham vs. DSS GV-Bi	$p = 0.002$
Fig.3H	EPM	total distance (m)							Two-way ANOVA Tukey's Multiple Comparisons test	H_2O Sham vs. H_2O SDV-Bi	$p = 0.006$
			Sham	WT	H_2O	Male	10	14.75 (12.28, 17.15)		H_2O Sham vs. DSS Sham	$p < 0.001$
			SDV-Bi	WT	H_2O	Male	10	16.00 (12.78, 19.15)		H_2O Sham vs. DSS SDV-Bi	$p < 0.001$
			Sham	WT	DSS	Male	13	4.55 (3.50, 5.48)		H_2O SDV-Bi vs. DSS Sham	$p < 0.001$
			**SDV-Bi**	**WT**	**DSS**	**Male**	**13**	**4.90 (3.35, 7.10)**		H_2O SDV-Bi vs. DSS SDV-Bi	$p = 0.007$
										DSS Sham vs. DSS SDV-Bi	$p = 0.800$
Fig.3H	EPM	total distance (m)							Two-way ANOVA Tukey's Multiple Comparisons test	H_2O Sham vs. H_2O SDV-Bi	$p < 0.001$
			Sham	WT	H_2O	Male	10	14.75 (12.28, 17.15)		H_2O Sham vs. DSS Sham	$p < 0.001$
			SDV-Bi	WT	H_2O	Male	10	16.00 (12.78, 19.15)		H_2O Sham vs. DSS SDV-R	$p < 0.001$
			Sham	WT	DSS	Male	13	4.55 (3.50, 5.48)		H_2O SDV-Bi vs. DSS Sham	$p < 0.001$
			**SDV-R**	**WT**	**DSS**	**Male**	**12**	**4.55 (3.93, 6.15)**		H_2O SDV-Bi vs. DSS SDV-R	$p < 0.001$
										DSS Sham vs. DSS SDV-R	$p > 0.999$
Fig.3H	EPM	total distance (m)							Two-way ANOVA Tukey's Multiple Comparisons test	H_2O Sham vs. H_2O SDV-Bi	$p = 0.002$
			Sham	WT	H_2O	Male	10	14.75 (12.28, 17.15)		H_2O Sham vs. DSS Sham	$p < 0.001$
			SDV-Bi	WT	H_2O	Male	10	16.00 (12.78, 19.15)		H_2O Sham vs. DSS V-L	$p < 0.001$
			Sham	WT	DSS	Male	13	4.55 (3.50, 5.48)		H_2O SDV-Bi vs. DSS Sham	$p < 0.001$
			**V-L**	**WT**	**DSS**	**Male**	**13**	**6.40 (4.40, 9.85)**		H_2O SDV-Bi vs. DSS V-L	$p = 0.022$
										DSS Sham vs. DSS V-L	$p = 0.308$

Figure	Parameter		Test specifications	Genotype	Treatment	Sex	Number of individuals	Median (Q1,Q3)	Statistical test	t-distribution t, df	*p* = value
Fig.3I	EPM	open arms (sec)	Sham	WT	H_2O	Male	10	6.05 (2.33, 15.58)	Mann-Whitney U test	-	*p* = 0.003
			Sham	WT	DSS	Male	13	0.40 (0.00, 1.30)			

Figure	Parameter		Test specifications	Genotype	Treatment	Sex	Number of individuals	Median (Q1,Q3)	Statistical test	Fixed effects F(DFn,DFd)	*p* = value
Fig.3I	EPM	open arms (sec)	Sham	WT	H_2O	Male	10	6.05 (2.33, 15.58)	Two-way ANOVA	DSS x Vagotomy $F_{(1,43)}$ = 11.90	*p* = 0.001
			GV-Bi	WT	H_2O	Male	10	6.25 (1.55, 12.68)			
			Sham	WT	DSS	Male	13	0.40 (0.00, 1.30)			
			**GV-Bi**	**WT**	**DSS**	**Male**	**14**	**10.08 (2.08, 24.25)**			
			Sham	WT	H_2O	Male	10	6.05 (2.33, 15.58)	Two-way ANOVA	DSS x Vagotomy $F_{(1,42)}$ = 3.90	*p* = 0.055
			GV-Bi	WT	H_2O	Male	10	6.25 (1.55, 12.68)			
			Sham	WT	DSS	Male	13	0.40 (0.00, 1.30)			
			**GV-R**	**WT**	**DSS**	**Male**	**13**	**0.40 (0.00, 5.85)**			
			Sham	WT	H_2O	Male	10	6.05 (2.33, 15.58)	Two-way ANOVA	DSS x Vagotomy $F_{(1,42)}$ = 4.31	*p* = 0.044
			GV-Bi	WT	H_2O	Male	10	6.25 (1.55, 12.68)			
			Sham	WT	DSS	Male	13	0.40 (0.00, 1.30)			
			**V-L**	**WT**	**DSS**	**Male**	**13**	**1.00 (0.10, 7.50)**			
Fig.3I	EPM	open arms (sec)	Sham	WT	H_2O	Male	10	6.05 (2.33, 15.58)	Two-way ANOVA	DSS x Vagotomy $F_{(1,43)}$ = 6.57	*p* = 0.014
			SDV-Bi	WT	H_2O	Male	10	9.05 (4.15, 12.50)			
			Sham	WT	DSS	Male	13	0.40 (0.00, 1.30)			
			**SDV-Bi**	**WT**	**DSS**	**Male**	**13**	**5.20 (1.45, 12.85)**			
			Sham	WT	H_2O	Male	10	6.05 (2.33, 15.58)	Two-way ANOVA	DSS x Vagotomy $F_{(1,41)}$ = 1.93	*p* = 0.172
			SDV-Bi	WT	H_2O	Male	10	9.05 (4.15, 12.50)			
			Sham	WT	DSS	Male	13	0.40 (0.00, 1.30)			
			**SDV-R**	**WT**	**DSS**	**Male**	**12**	**0.75 (0.03, 2.15)**			
			Sham	WT	H_2O	Male	10	6.05 (2.33, 15.58)	Two-way ANOVA	DSS x Vagotomy $F_{(1,42)}$ = 4.03	*p* = 0.051
			SDV-Bi	WT	H_2O	Male	10	9.05 (4.15, 12.50)			
			Sham	WT	DSS	Male	13	0.40 (0.00, 1.30)			
			**V-L**	**WT**	**DSS**	**Male**	**13**	**1.00 (0.10, 7.50)**			

Figure	Parameter		Test specifications	Genotype	Treatment	Sex	Number of individuals	Median (Q1,Q3)	Statistical test	Compared Groups	*p* = value
Fig.3I	EPM	open arms (sec)							Two-way ANOVA Tukey's Multiple Comparisons test	H_2O Sham vs. H_2O GV-Bi	*p* = 0.741
			Sham	WT	H_2O	Male	10	6.05 (2.33, 15.58)		H_2O Sham vs. DSS Sham	*p* = 0.009
			GV-Bi	WT	H_2O	Male	10	6.25 (1.55, 12.68)		H_2O Sham vs. DSS GV-Bi	*p* = 0.977
			Sham	WT	DSS	Male	13	0.40 (0.00, 1.30)		H_2O GV-Bi vs. DSS Sham	*p* = 0.119
			**GV-Bi**	**WT**	**DSS**	**Male**	**14**	**10.08 (2.08, 24.25)**		H_2O GV-Bi vs. DSS GV-Bi	*p* = 0.443
										DSS Sham vs. DSS GV-Bi	*p* = 0.001
Fig.3I	EPM	open arms (sec)							Two-way ANOVA Tukey's Multiple Comparisons test	H_2O Sham vs. H_2O GV-Bi	*p* = 0.423
			Sham	WT	H_2O	Male	10	6.05 (2.33, 15.58)		H_2O Sham vs. DSS Sham	*p* < 0.001
			GV-Bi	WT	H_2O	Male	10	6.25 (1.55, 12.68)		H_2O Sham vs. DSS V-L	*p* = 0.003
			Sham	WT	DSS	Male	13	0.40 (0.00, 1.30)		H_2O GV-Bi vs. DSS Sham	*p* = 0.007
			**V-L**	**WT**	**DSS**	**Male**	**13**	**1.00 (0.10, 7.50)**		H_2O GV-Bi vs. DSS V-L	*p* = 0.152
										DSS Sham vs. DSS V-L	*p* = 0.512
Fig.3I	EPM	open arms (sec)							Two-way ANOVA Tukey's Multiple Comparisons test	H_2O Sham vs. H_2O SV-Bi	*p* = 0.860
			Sham	WT	H_2O	Male	10	6.05 (2.33, 15.58)		H_2O Sham vs. DSS Sham	*p* = 0.021
			SDV-Bi	WT	H_2O	Male	10	9.05 (4.15, 12.50)		H_2O Sham vs. DSS SDV-Bi	*p* = 0.993
			Sham	WT	DSS	Male	13	0.40 (0.00, 1.30)		H_2O SDV-Bi vs. DSS Sham	*p* = 0.142
			**SDV-Bi**	**WT**	**DSS**	**Male**	**13**	**5.20 (1.45, 12.85)**		H_2O SDV-Bi vs. DSS SDV-Bi	*p* = 0.938
										DSS Sham vs. DSS SDV-Bi	*p* = 0.022

Figure	Parameter		Test specifications	Genotype	Treatment	Sex	Number of individuals	Median (Q1,Q3)	Statistical test	t-distribution t, df	*p* = value
Fig.3J	EPM	closed arms (sec)	Sham	WT	H_2O	Male	10	79.00 (66.95, 88.78)	Two-tailed unpaired Student's t-test	$t_{(21)} = 4.74$	$p < 0.001$
			Sham	WT	DSS	Male	13	96.00 (90.95, 99.30)			

Figure	Parameter		Test specifications	Genotype	Treatment	Sex	Number of individuals	Median (Q1,Q3)	Statistical test	Fixed effects F(DFn,DFd)	*p* = value
Fig.3J	EPM	closed arms (sec)	Sham	WT	H_2O	Male	10	79.00 (66.95, 86.78)	Two-way ANOVA	DSS x Vagotomy $F_{(1,43)} = 10.22$	$p = 0.003$
			GV-Bi	WT	H_2O	Male	10	72.00 (60.83, 82.48)			
			Sham	WT	DSS	Male	13	96.00 (90.95, 99.30)			
			**GV-Bi**	**WT**	**DSS**	**Male**	**14**	**80.35 (62.70, 89.93)**			
			Sham	WT	H_2O	Male	10	79.00 (66.95, 86.78)	Two-way ANOVA	DSS x Vagotomy $F_{(1,42)} = 2.58$	$p = 0.116$
			GV-Bi	WT	H_2O	Male	10	72.00 (60.83, 82.48)			
			Sham	WT	DSS	Male	13	96.00 (90.95, 99.30)			
			**GV-R**	**WT**	**DSS**	**Male**	**13**	**91.00 (82.85, 98.40)**			
			Sham	WT	H_2O	Male	10	79.00 (66.95, 86.78)	Two-way ANOVA	DSS x Vagotomy $F_{(1,42)} = 2.66$	$p = 0.110$
			GV-Bi	WT	H_2O	Male	10	72.00 (60.83, 82.48)			
			Sham	WT	DSS	Male	13	96.00 (90.95, 99.30)			
			**V-L**	**WT**	**DSS**	**Male**	**13**	**93.90 (79.35, 96.10)**			
Fig.3J	EPM	closed arms (sec)	Sham	WT	H_2O	Male	10	79.00 (66.95, 86.78)	Two-way ANOVA	DSS x Vagotomy $F_{(1,43)} = 14.16$	$p < 0.001$
			SDV-Bi	WT	H_2O	Male	10	78.25 (72.25, 84.75)			
			Sham	WT	DSS	Male	13	96.00 (90.95, 99.30)			
			**SDV-Bi**	**WT**	**DSS**	**Male**	**13**	**89.20 (74.90, 93.80)**			
			Sham	WT	H_2O	Male	10	79.00 (66.95, 86.78)	Two-way ANOVA	DSS x Vagotomy $F_{(1,41)} = 6.31$	$p = 0.016$
			SDV-Bi	WT	H_2O	Male	10	78.25 (72.25, 84.75)			
			Sham	WT	DSS	Male	13	96.00 (90.95, 99.30)			
			**SDV-R**	**WT**	**DSS**	**Male**	**12**	**92.95 (90.13, 96.35)**			
			Sham	WT	H_2O	Male	10	79.00 (66.95, 86.78)	Two-way ANOVA	DSS x Vagotomy $F_{(1,42)} = 9.14$	$p = 0.004$
			SDV-Bi	WT	H_2O	Male	10	78.25 (72.25, 84.75)			
			Sham	WT	DSS	Male	13	96.00 (90.95, 99.30)			
			**V-L**	**WT**	**DSS**	**Male**	**13**	**93.90 (79.35, 96.10)**			

Figure	Parameter		Test specifications	Genotype	Treatment	Sex	Number of individuals	Median (Q1,Q3)	Statistical test	Compared Groups	*p* = value
Fig.3J	EPM	closed arms (sec)							Two-way ANOVA Tukey's Multiple Comparisons test	H_2O Sham vs. H_2O GV-Bi	$p = 0.906$
			Sham	WT	H_2O	Male	10	79.00 (66.95, 86.78)		H_2O Sham vs. DSS Sham	$p < 0.001$
			GV-Bi	WT	H_2O	Male	10	72.00 (60.83, 82.48)		H_2O Sham vs. DSS GV-Bi	$p = 0.472$
			Sham	WT	DSS	Male	13	96.00 (90.95, 99.30)		H_2O GV-Bi vs. DSS Sham	$p < 0.001$
			**GV-Bi**	**WT**	**DSS**	**Male**	**14**	**80.35 (62.70, 89.93)**		H_2O GV-Bi vs. DSS GV-Bi	$p = 0.886$
										DSS Sham vs. DSS GV-Bi	$p = 0.001$
Fig.3J	EPM	closed arms (sec)							Two-way ANOVA Tukey's Multiple Comparisons test	H_2O Sham vs. H_2O SDV-Bi	$p = 0.157$
			Sham	WT	H_2O	Male	10	79.00 (66.95, 86.78)		H_2O Sham vs. DSS Sham	$p < 0.001$
			SDV-Bi	WT	H_2O	Male	10	78.25 (72.25, 84.75)		H_2O Sham vs. DSS SDV-Bi	$p = 0.046$
			Sham	WT	DSS	Male	13	96.00 (90.95, 99.30)		H_2O SDV-Bi vs. DSS Sham	$p = 0.008$
			**SDV-Bi**	**WT**	**DSS**	**Male**	**13**	**89.20 (74.90, 93.80)**		H_2O SDV-Bi vs. DSS SDV-Bi	$p = 0.979$
										DSS Sham vs. DSS SDV-Bi	$p = 0.011$
Fig.3J	EPM	closed arms (sec)							Two-way ANOVA Tukey's Multiple Comparisons test	H_2O Sham vs. H_2O SDV-Bi	$p = 0.025$
			Sham	WT	H_2O	Male	10	79.00 (66.95, 86.78)		H_2O Sham vs. DSS Sham	$p < 0.001$
			SDV-Bi	WT	H_2O	Male	10	78.25 (72.25, 84.75)		H_2O Sham vs. DSS SDV-R	$p < 0.001$
			Sham	WT	DSS	Male	13	96.00 (90.95, 99.30)		H_2O SDV-Bi vs. DSS Sham	$p < 0.001$
			**SDV-R**	**WT**	**DSS**	**Male**	**12**	**92.95 (90.13, 96.35)**		H_2O SDV-Bi vs. DSS SDV-R	$p < 0.001$
										DSS Sham vs. DSS SDV-R	$p = 0.969$

Figure	Parameter		Test specifications	Genotype	Treatment	Sex	Number of individuals	Median (Q1,Q3)	Statistical test	Compared Groups	*p* = value
Fig.3J	EPM	closed arms (sec)							Two-way ANOVA<br>Tukey's Multiple Comparisons test	H_2O Sham vs. H_2O SV-Bi	$p = 0.069$
			Sham	WT	H_2O	Male	10	79.00 (66.95, 86.78)		H_2O Sham vs. DSS Sham	$p < 0.001$
			SDV-Bi	WT	H_2O	Male	10	78.25 (72.25, 84.75)		H_2O Sham vs. DSS V-L	$p < 0.001$
			Sham	WT	DSS	Male	13	96.00 (90.95, 99.30)		H_2O SDV-Bi vs. DSS Sham	$p = 0.001$
			**V-L**	**WT**	**DSS**	**Male**	**13**	**93.90 (79.35, 96.10)**		H_2O SDV-Bi vs. DSS V-L	$p = 0.084$
										DSS Sham vs. DSS V-L	$p = 0.340$

Figure	Parameter	Test specifications	Genotype	Treatment	Sex	Number of individuals	Mean ± SEM	Statistical test	Dunnett's test	*p* = value
Fig.4C	Food intake (g)	SAP-Blank	WT	-	Male	21	0.59 ± 0.04	One-way ANOVA<br>($p = 0.016$)	-	-
		CCK-SAP-Bi	WT	-	Male	21	0.80 ± 0.05		SAP-Blank vs. CCK-SAP-Bi	$p = 0.001$
		CCK-SAP-R	WT	-	Male	10	0.79 ± 0.05		SAP-Blank vs. CCK-SAP-R	$p = 0.015$
		CCK-SAP-L	WT	-	Male	10	0.76 ± 0.03		SAP-Blank vs. CCK-SAP-L	$p = 0.045$

Figure	Parameter		Test specifications	Genotype	Treatment	Sex	Number of individuals	Median (Q1,Q3)	Statistical test	t-distribution t, df	*p* = value
Fig.4D	OFT	total distance (m)	SAP-Blank	WT	H_2O	Male	9	33.90 (28.65, 36.55)	Two-tailed unpaired Student's t-test	$t_{(19)} = 5.19$	$p < 0.001$
			SAP-Blank	WT	DSS	Male	12	18.15 (13.38, 25.73)			

Figure	Parameter		Test specifications	Genotype	Treatment	Sex	Number of individuals	Median (Q1,Q3)	Statistical test	Fixed effects F(DFn,DFd)	*p* = value
Fig.4D	OFT	total distance (m)	SAP-Blank	WT	H_2O	Male	9	33.90 (28.65, 36.55)	Two-way ANOVA	DSS x Nodose CCK-SAP<br>$F_{(1,38)} = 0.28$	$p = 0.603$
			CCK-SAP-Bi	WT	H_2O	Male	9	34.10 (30.10, 40.95)			
			SAP-Blank	WT	DSS	Male	12	18.15 (13.38, 25.73)			
			**CCK-SAP**	**WT**	**DSS**	**Male**	**12**	**16.85 (12.90, 21.45)**			
			SAP-Blank	WT	H_2O	Male	9	33.90 (28.65, 36.55)	Two-way ANOVA	DSS x Nodose CCK-SAP<br>$F_{(1,36)} = 0.10$	$p = 0.750$
			CCK-SAP-Bi	WT	H_2O	Male	9	34.10 (30.10, 40.95)			
			SAP-Blank	WT	DSS	Male	12	18.15 (13.38, 25.73)			
			**CCK-SAP-R**	**WT**	**DSS**	**Male**	**10**	**18.15 (14.83, 20.90)**			
			SAP-Blank	WT	H_2O	Male	9	33.90 (28.65, 36.55)	Two-way ANOVA	DSS x Nodose CCK-SAP<br>$F_{(1,36)} = 3.16$	$p = 0.084$
			CCK-SAP-Bi	WT	H_2O	Male	9	34.10 (30.10, 40.95)			
			SAP-Blank	WT	DSS	Male	12	18.15 (13.38, 25.73)			
			**CCK-SAP-L**	**WT**	**DSS**	**Male**	**10**	**12.00 (9.90, 16.33)**			

Figure	Parameter		Test specifications	Genotype	Treatment	Sex	Number of individuals	Median (Q1,Q3)	Statistical test	t-distribution t, df	*p* = value
Fig.4E	OFT	time in center (sec)	SAP-Blank	WT	H_2O	Male	9	9.10 (4.60, 15.95)	Two-tailed unpaired Student's t-test	$t_{(19)} = 4.27$	$p < 0.001$
			SAP-Blank	WT	DSS	Male	12	1.80 (0.60, 3.98)			

Figure	Parameter		Test specifications	Genotype	Treatment	Sex	Number of individuals	Median (Q1,Q3)	Statistical test	Fixed effects F(DFn,DFd)	*p* = value
Fig.4E	OFT	time in center (sec)	SAP-Blank	WT	H_2O	Male	9	9.10 (4.60, 15.95)	Two-way ANOVA	DSS x Nodose CCK-SAP<br>$F_{(1,38)} = 2.68$	$p = 0.110$
			CCK-SAP-Bi	WT	H_2O	Male	9	7.90 (6.25, 14.55)			
			SAP-Blank	WT	DSS	Male	12	1.80 (0.60, 3.98)			
			**CCK-SAP**	**WT**	**DSS**	**Male**	**12**	**1.60 (0.60, 3.35)**			
			SAP-Blank	WT	H_2O	Male	9	9.10 (4.60, 15.95)	Two-way ANOVA	DSS x Nodose CCK-SAP<br>$F_{(1,36)} = 2.55$	$p = 0.119$
			CCK-SAP-Bi	WT	H_2O	Male	9	7.90 (6.25, 14.55)			
			SAP-Blank	WT	DSS	Male	12	1.80 (0.60, 3.98)			
			**CCK-SAP-R**	**WT**	**DSS**	**Male**	**10**	**1.35 (0.38, 2.85)**			
			SAP-Blank	WT	H_2O	Male	9	9.10 (4.60, 15.95)	Two-way ANOVA	DSS x Nodose CCK-SAP<br>$F_{(1,36)} = 2.59$	$p = 0.116$
			CCK-SAP-Bi	WT	H_2O	Male	9	7.90 (6.25, 14.55)			
			SAP-Blank	WT	DSS	Male	12	1.80 (0.60, 3.98)			
			**CCK-SAP-L**	**WT**	**DSS**	**Male**	**10**	**1.95 (0.55, 3.00)**			

Figure	Parameter		Test specifications	Genotype	Treatment	Sex	Number of individuals	Median (Q1,Q3)	Statistical test	t-distribution t, df	*p* = value
Fig.4F	LDB	duration (sec)	SAP-Blank	WT	H_2O	Male	9	33.50 (22.90, 40.75)	Mann-Whitney U test	-	$p < 0.001$
			SAP-Blank	WT	DSS	Male	12	3.90 (0.00, 9.50)			

Figure	Parameter		Test specifications	Genotype	Treatment	Sex	Number of individuals	Median (Q1,Q3)	Statistical test	Fixed effects F(DFn,DFd)	*p* = value
Fig.4F	LDB	duration (sec)	SAP-Blank	WT	H_2O	Male	9	33.50 (22.90, 40.75)	Two-way ANOVA	DSS x Nodose CCK-SAP $F_{(1,38)} = 35.09$	$p < 0.001$
			CCK-SAP-Bi	WT	H_2O	Male	9	36.30 (16.85, 39.10)			
			SAP-Blank	WT	DSS	Male	12	3.90 (0.00, 9.50)			
			**CCK-SAP**	**WT**	**DSS**	**Male**	**12**	**42.60 (27.80, 50.38)**			
			SAP-Blank	WT	H_2O	Male	9	33.50 (22.90, 40.75)	Two-way ANOVA	DSS x Nodose CCK-SAP $F_{(1,36)} = 2.31$	$p = 0.137$
			CCK-SAP-Bi	WT	H_2O	Male	9	36.30 (16.85, 39.10)			
			SAP-Blank	WT	DSS	Male	12	3.90 (0.00, 9.50)			
			**CCK-SAP-R**	**WT**	**DSS**	**Male**	**10**	**9.50 (0.75, 18.75)**			
			SAP-Blank	WT	H_2O	Male	9	33.50 (22.90, 40.75)	Two-way ANOVA	DSS x Nodose CCK-SAP $F_{(1,36)} = 2.57$	$p = 0.118$
			CCK-SAP-Bi	WT	H_2O	Male	9	36.30 (16.85, 39.10)			
			SAP-Blank	WT	DSS	Male	12	3.90 (0.00, 9.50)			
			**CCK-SAP-L**	**WT**	**DSS**	**Male**	**10**	**9.45 (0.53, 19.05)**			

Figure	Parameter		Test specifications	Genotype	Treatment	Sex	Number of individuals	Median (Q1,Q3)	Statistical test	Compared Groups	*p* = value
Fig.4F	LDB	duration (sec)							Two-way ANOVA Tukey's Multiple Comparisons test	H_2O SAP-B vs. H_2O CCK-SAP	$p = 0.841$
			SAP-Blank	WT	H_2O	Male	9	33.50 (22.90, 40.75)		H_2O SAP-B vs. DSS SAP-B	$p < 0.001$
			CCK-SAP-Bi	WT	H_2O	Male	9	36.30 (16.85, 39.10)		H_2O SAP-B vs. DSS CCK-SAP	$p = 0.681$
			SAP-Blank	WT	DSS	Male	12	3.90 (0.00, 9.50)		H_2O CCK-SAP vs. DSS SAP-B	$p < 0.001$
			**CCK-SAP**	**WT**	**DSS**	**Male**	**12**	**42.60 (27.80, 50.38)**		H_2O CCK-SAP vs. DSS CCK-SAP	$p = 0.205$
										DSS SAP-B vs. DSS CCK-SAP	$p < 0.001$

Figure	Parameter		Test specifications	Genotype	Treatment	Sex	Number of individuals	Median (Q1,Q3)	Statistical test	t-distribution t, df	*p* = value
Fig.4G	LDB	entries	SAP-Blank	WT	H_2O	Male	9	8.50 (3.00, 12.00)	Two-tailed unpaired Student's t-test	$t_{(19)} = 4.98$	$p < 0.001$
			SAP-Blank	WT	DSS	Male	12	1.00 (0.00, 3.00)			

Figure	Parameter		Test specifications	Genotype	Treatment	Sex	Number of individuals	Median (Q1,Q3)	Statistical test	Fixed effects F(DFn,DFd)	*p* = value
Fig.4G	LDB	entries	SAP-Blank	WT	H_2O	Male	9	8.50 (3.00, 12.00)	Two-way ANOVA	DSS x Nodose CCK-SAP $F_{(1,38)} = 8.53$	$p = 0.006$
			CCK-SAP-Bi	WT	H_2O	Male	9	7.00 (5.00, 11.00)			
			SAP-Blank	WT	DSS	Male	12	1.00 (0.00, 3.00)			
			**CCK-SAP**	**WT**	**DSS**	**Male**	**12**	**8.00 (6.00, 10.00)**			
			SAP-Blank	WT	H_2O	Male	9	8.50 (3.00, 12.00)	Two-way ANOVA	DSS x Nodose CCK-SAP $F_{(1,36)} = 0.004$	$p = 0.948$
			CCK-SAP-Bi	WT	H_2O	Male	9	7.00 (5.00, 11.00)			
			SAP-Blank	WT	DSS	Male	12	1.00 (0.00, 3.00)			
			**CCK-SAP-R**	**WT**	**DSS**	**Male**	**10**	**0.75 (0.00, 2.50)**			
			SAP-Blank	WT	H_2O	Male	9	8.50 (3.00, 12.00)	Two-way ANOVA	DSS x Nodose CCK-SAP $F_{(1,36)} = 0.008$	$p = 0.926$
			CCK-SAP-Bi	WT	H_2O	Male	9	7.00 (5.00, 11.00)			
			SAP-Blank	WT	DSS	Male	12	1.00 (0.00, 3.00)			
			**CCK-SAP-L**	**WT**	**DSS**	**Male**	**10**	**0.75 (0.00, 3.00)**			

Figure	Parameter		Test specifications	Genotype	Treatment	Sex	Number of individuals	Median (Q1,Q3)	Statistical test	Compared Groups	*p* = value
Fig.4G	LDB	entries							Two-way ANOVA Tukey's Multiple Comparisons test	H_2O SAP-B vs. H_2O CCK-SAP	$p = 0.995$
			SAP-Blank	WT	H_2O	Male	9	8.50 (3.00, 12.00)		H_2O SAP-B vs. DSS SAP-B	$p < 0.001$
			CCK-SAP-Bi	WT	H_2O	Male	9	7.00 (5.00, 11.00)		H_2O SAP-B vs. DSS CCK-SAP	$p = 0.722$
			SAP-Blank	WT	DSS	Male	12	1.00 (0.00, 3.00)		H_2O CCK-SAP vs. DSS SAP-B	$p < 0.001$

Figure	Parameter		Test specifications	Genotype	Treatment	Sex	Number of individuals	Median (Q1,Q3)	Statistical test	Compared Groups	p = value
Fig.4G	LDB	entries	**CCK-SAP**	**WT**	**DSS**	**Male**	**12**	**8.00 (6.00, 10.00)**		H_2O CCK-SAP vs. DSS CCK-SAP	$p = 0.559$
										DSS SAP-B vs. DSS CCK-SAP	$p < 0.001$

Figure	Parameter		Test specifications	Genotype	Treatment	Sex	Number of individuals	Median (Q1,Q3)	Statistical test	t-distribution t, df	p = value
Fig.4H	EPM	total distance (m)	SAP-Blank	WT	H_2O	Male	9	11.80 (9.30, 14.60)	Two-tailed unpaired Student's t-test	$t_{(19)} = 4.98$	$p = 0.036$
			SAP-Blank	WT	DSS	Male	12	8.65 (5.63, 10.45)			

Figure	Parameter		Test specifications	Genotype	Treatment	Sex	Number of individuals	Median (Q1,Q3)	Statistical test	Fixed effects F(DFn,DFd)	p = value
Fig.4H	EPM	total distance (m)	SAP-Blank	WT	H_2O	Male	9	11.80 (9.30, 14.60)	Two-way ANOVA	DSS x Nodose CCK-SAP $F_{(1,38)} = 1.71$	$p = 0.198$
			CCK-SAP-Bi	WT	H_2O	Male	9	13.50 (10.50, 17.90)			
			SAP-Blank	WT	DSS	Male	12	8.65 (5.63, 10.45)			
			**CCK-SAP**	**WT**	**DSS**	**Male**	**12**	**12.45 (10.78, 19.38)**			
			SAP-Blank	WT	H_2O	Male	9	11.80 (9.30, 14.60)	Two-way ANOVA	DSS x Nodose CCK-SAP $F_{(1,36)} = 3.72$	$p = 0.062$
			CCK-SAP-Bi	WT	H_2O	Male	9	13.50 (10.50, 17.90)			
			SAP-Blank	WT	DSS	Male	12	8.65 (5.63, 10.45)			
			**CCK-SAP-R**	**WT**	**DSS**	**Male**	**10**	**4.30 (3.80, 6.28)**			
			SAP-Blank	WT	H_2O	Male	9	11.80 (9.30, 14.60)	Two-way ANOVA	DSS x Nodose CCK-SAP $F_{(1,36)} = 7.14$	$p = 0.011$
			CCK-SAP-Bi	WT	H_2O	Male	9	13.50 (10.50, 17.90)			
			SAP-Blank	WT	DSS	Male	12	8.65 (5.63, 10.45)			
			**CCK-SAP-L**	**WT**	**DSS**	**Male**	**10**	**4.15 (2.70, 5.43)**			

Figure	Parameter		Test specifications	Genotype	Treatment	Sex	Number of individuals	Median (Q1,Q3)	Statistical test	t-distribution t, df	p = value
Fig.4I	EPM	open arms (sec)	SAP-Blank	WT	H_2O	Male	9	1.80 (0.05, 3.75)	Two-tailed unpaired Student's t-test	$t_{(19)} = 2.10$	$p = 0.050$
			SAP-Blank	WT	DSS	Male	12	0.40 (0.15, 0.75)			

Figure	Parameter		Test specifications	Genotype	Treatment	Sex	Number of individuals	Median (Q1,Q3)	Statistical test	Fixed effects F(DFn,DFd)	p = value
Fig.4I	EPM	open arms (sec)	SAP-Blank	WT	H_2O	Male	9	1.80 (0.05, 3.75)	Two-way ANOVA	DSS x Nodose CCK-SAP $F_{(1,38)} = 10.07$	$p = 0.003$
			CCK-SAP-Bi	WT	H_2O	Male	9	2.40 (0.85, 8.50)			
			SAP-Blank	WT	DSS	Male	12	0.40 (0.15, 0.75)			
			**CCK-SAP**	**WT**	**DSS**	**Male**	**12**	**8.15 (3.53, 12.68)**			
			SAP-Blank	WT	H_2O	Male	9	1.80 (0.05, 3.75)	Two-way ANOVA	DSS x Nodose CCK-SAP $F_{(1,36)} = 1.23$	$p = 0.276$
			CCK-SAP-Bi	WT	H_2O	Male	9	2.40 (0.85, 8.50)			
			SAP-Blank	WT	DSS	Male	12	0.40 (0.15, 0.75)			
			**CCK-SAP-R**	**WT**	**DSS**	**Male**	**10**	**0.80 (0.38, 2.00)**			
			SAP-Blank	WT	H_2O	Male	9	1.80 (0.05, 3.75)	Two-way ANOVA	DSS x Nodose CCK-SAP $F_{(1,36)} = 1.85$	$p = 0.182$
			CCK-SAP-Bi	WT	H_2O	Male	9	2.40 (0.85, 8.50)			
			SAP-Blank	WT	DSS	Male	12	0.40 (0.15, 0.75)			
			**CCK-SAP-L**	**WT**	**DSS**	**Male**	**10**	**0.15 (0.00, 2.80)**			

Figure	Parameter		Test specifications	Genotype	Treatment	Sex	Number of individuals	Median (Q1,Q3)	Statistical test	Compared Groups	p = value
Fig.4I	EPM	open arms (sec)							Two-way ANOVA Tukey's Multiple Comparisons test	H_2O SAP-B vs. H_2O CCK-SAP	$p = 0.857$
			SAP-Blank	WT	H_2O	Male	9	1.80 (0.05, 3.75)		H_2O SAP-B vs. DSS SAP-B	$p = 0.063$
			CCK-SAP-Bi	WT	H_2O	Male	9	2.40 (0.85, 8.50)		H_2O SAP-B vs. DSS CCK-SAP	$p = 0.719$
			SAP-Blank	WT	DSS	Male	12	0.40 (0.15, 0.75)		H_2O CCK-SAP vs. DSS SAP-B	$p = 0.319$
			**CCK-SAP**	**WT**	**DSS**	**Male**	**12**	**8.15 (3.53, 12.68)**		H_2O CCK-SAP vs. DSS CCK-SAP	$p = 0.245$
										DSS SAP-B vs. DSS CCK-SAP	$p = 0.002$

Figure	Parameter		Test specifications	Genotype	Treatment	Sex	Number of individuals	Median (Q1,Q3)	Statistical test	t-distribution t, df	*p* = value
Fig.4J	EPM	closed arms (sec)	SAP-Blank	WT	H_2O	Male	9	86.00 (77.15, 92.20)	Two-tailed unpaired Student's t-test	$t_{(19)} = 3.34$	$p = 0.003$
			SAP-Blank	WT	DSS	Male	12	93.05 (91.05, 94.73)			

Figure	Parameter		Test specifications	Genotype	Treatment	Sex	Number of individuals	Median (Q1,Q3)	Statistical test	Fixed effects F(DFn,DFd)	*p* = value
Fig.4J	EPM	closed arms (sec)	SAP-Blank	WT	H_2O	Male	9	86.00 (77.15, 92.20)	Two-way ANOVA	DSS x Nodose CCK-SAP $F_{(1,38)} = 7.72$	$p = 0.008$
			CCK-SAP-Bi	WT	H_2O	Male	9	77.20 (70.70, 86.25)			
			SAP-Blank	WT	DSS	Male	12	93.05 (91.05, 94.73)			
			**CCK-SAP**	**WT**	**DSS**	**Male**	**12**	**78.45 (68.35, 85.48)**			
			SAP-Blank	WT	H_2O	Male	9	86.00 (77.15, 92.20)	Two-way ANOVA	DSS x Nodose CCK-SAP $F_{(1,36)} = 0.001$	$p = 0.974$
			CCK-SAP-Bi	WT	H_2O	Male	9	77.20 (70.70, 86.25)			
			SAP-Blank	WT	DSS	Male	12	93.05 (91.05, 94.73)			
			**CCK-SAP-R**	**WT**	**DSS**	**Male**	**10**	**94.25 (89.20, 95.78)**			
			SAP-Blank	WT	H_2O	Male	9	86.00 (77.15, 92.20)	Two-way ANOVA	DSS x Nodose CCK-SAP $F_{(1,36)} = 0.002$	$p = 0.962$
			CCK-SAP-Bi	WT	H_2O	Male	9	77.20 (70.70, 86.25)			
			SAP-Blank	WT	DSS	Male	12	93.05 (91.05, 94.73)			
			**CCK-SAP-L**	**WT**	**DSS**	**Male**	**10**	**96.05 (91.48, 98.38)**			

Figure	Parameter		Test specifications	Genotype	Treatment	Sex	Number of individuals	Median (Q1,Q3)	Statistical test	Compared Groups	*p* = value
Fig.4J	EPM	closed arms (sec)							Two-way ANOVA Tukey's Multiple Comparisons test	H_2O SAP-B vs. H_2O CCK-SAP	$p > 0.999$
			SAP-Blank	WT	H_2O	Male	9	86.00 (77.15, 92.20)		H_2O SAP-B vs. DSS SAP-B	$p = 0.001$
			CCK-SAP-Bi	WT	H_2O	Male	9	77.20 (70.70, 86.25)		H_2O SAP-B vs. DSS CCK-SAP	$p = 0.940$
			SAP-Blank	WT	DSS	Male	12	93.05 (91.05, 94.73)		H_2O CCK-SAP vs. DSS SAP-B	$p = 0.011$
			**CCK-SAP**	**WT**	**DSS**	**Male**	**12**	**78.45 (68.35, 85.48)**		H_2O CCK-SAP vs. DSS CCK-SAP	$p = 0.933$
										DSS SAP-B vs. DSS CCK-SAP	$p < 0.001$

Figure	Parameter	Brain region	Test specifications	Genotype	Treatment	Sex	Number (sections)	Mean ± SEM	Statistical test	t-distribution t, df	*p* = value
Fig.5B	c-Fos neurons	NTS	H2O	WT	-	Male	5 (37)	3.58 ± 0.48	Two-tailed unpaired Student's t-test	$t_{(8)} = 8.89$	$p < 0.001$
			DSS	WT	-	Male	5 (43)	13.02 ± 0.95			
Fig.5D	c-Fos neurons	LC	H2O	WT	-	Male	5 (21)	19.92 ± 2.10	Two-tailed unpaired Student's t-test	$t_{(8)} = 11.83$	$p < 0.001$
			DSS	WT	-	Male	5 (26)	61.14 ± 2.78			
Fig.5F	c-Fos neurons	BLA	H2O	WT	-	Male	5 (39)	9.16 ± 1.42	Two-tailed unpaired Student's t-test	$t_{(8)} = 3.01$	$p = 0.017$
			DSS	WT	-	Male	5 (35)	14.62 ± 1.13			

Figure	Parameter		Test specifications	Genotype	Treatment	Sex	Number of individuals	Median (Q1,Q3)	Statistical test	t-distribution t, df	*p* = value
Fig.7C	OFT	total distance (m)	Saline	DBH-Cre	DSS	Male	10	22.65 (10.79, 28.70)	Two-tailed unpaired Student's t-test	$t_{(18)} = 0.45$	$p = 0.656$
			CNO	DBH-Cre	DSS	Male	10	24.49 (20.90, 30.98)			
Fig.7D		time in center (sec)	Saline	DBH-Cre	DSS	Male	10	1.65 (0.35, 5.38)	Two-tailed unpaired Student's t-test	$t_{(18)} = 3.34$	$p = 0.004$
			CNO	DBH-Cre	DSS	Male	10	8.10 (7.00, 13.28)			

Figure	Parameter		Test specifications	Genotype	Treatment	Sex	Number of individuals	Median (Q1,Q3)	Statistical test	t-distribution t, df	*p* = value
Fig.7E	LDB	duration (sec)	Saline	DBH-Cre	DSS	Male	10	29.00 (20.75, 46.50)	Two-tailed unpaired Student's t-test	$t_{(18)} = 3.09$	$p = 0.006$
			CNO	DBH-Cre	DSS	Male	10	53.50 (44.00, 62.00)			
Fig.7F		entries	Saline	DBH-Cre	DSS	Male	10	6.50 (5.00, 9.25)	Two-tailed unpaired Student's t-test	$t_{(18)} = 3.25$	$p = 0.004$
			CNO	DBH-Cre	DSS	Male	10	13.00 (8.50, 18.00)			
Fig.7G	EPM	total distance (m)	Saline	DBH-Cre	DSS	Male	10	7.61 (6.73, 11.94)	Two-tailed unpaired Student's t-test	$t_{(18)} = 2.12$	$p = 0.04$
			CNO	DBH-Cre	DSS	Male	10	11.54 (8.48, 20.31)			
Fig.7H		open arms (sec)	Saline	DBH-Cre	DSS	Male	10	6.90 (0.73, 15.70)	Two-tailed unpaired Student's t-test	$t_{(18)} = 2.78$	$p = 0.012$
			CNO	DBH-Cre	DSS	Male	10	21.45 (9.63, 42.85)			

Figure	Parameter		Test specifications	Genotype	Treatment	Sex	Number of individuals	Median (Q1,Q3)	Statistical test	t-distribution t, df	*p* = value
Fig.7I	EPM	closed arms (sec)	Saline	DBH-Cre	DSS	Male	10	74.95 (53.73, 87.48)	Two-tailed unpaired Student's t-test	t(18) = 2.03	p = 0.057
			CNO	DBH-Cre	DSS	Male	10	53.95 (35.80, 81.05)			

Figure	Parameter		Test specifications	Genotype	Treatment	Sex	Number of individuals	Median (Q1,Q3)	Statistical test	t-distribution t, df	*p* = value
Supplementary Fig.1B	OF	Number of rearings	H_2O	WT	-	Male	13	44.0 (39.50, 61.00)	Two-tailed unpaired Student's t-test	$t_{(23)} = 6.66$	$p < 0.001$
			DSS	WT	-	Male	12	18.0 (10.75, 22.00)			
Supplementary Fig.1C	LDB	Number of nose pokes	H_2O	WT	-	Male	13	5.0 (4.00, 7.00)	Two-tailed unpaired Student's t-test	$t_{(23)} = 0.68$	$p = 0.504$
			DSS	WT	-	Male	12	4.0 (2.25, 6.75)			
Supplementary Fig.1D	EPM	Number of rearings	H_2O	WT	-	Male	13	14.0 (10.50, 16.00)	Mann-Whitney U test	-	$p < 0.001$
			DSS	WT	-	Male	12	4.0 (1.25, 6.75)			
Supplementary Fig.1E	EPM	Number of stretches	H_2O	WT	-	Male	13	9.0 (8.00, 12.00)	Two-tailed unpaired Student's t-test	$t_{(23)} = 5.46$	$p < 0.001$
			DSS	WT	-	Male	12	3.5 (2.00, 5.75)			
Supplementary Fig.1F	OF	Number of rearings	Sham	WT	DSS	Male	13	7.0 (2.00, 11.50)	Two-tailed unpaired Student's t-test	$t_{(25)} = 4.44$	$p < 0.001$
			GV-Bi	WT	DSS	Male	14	21.5 (16.50, 29.25)			
Supplementary Fig.1G	LDB	Number of nose pokes	Sham	WT	DSS	Male	13	4.0 (2.00, 5.00)	Two-tailed unpaired Student's t-test	$t_{(25)} = 0.24$	$p = 0.811$
			GV-Bi	WT	DSS	Male	14	4.0 (3.00, 6.00)			
Supplementary Fig.1H	EPM	Number of rearings	Sham	WT	DSS	Male	13	2.0 (0.00, 4.50)	Two-tailed unpaired Student's t-test	$t_{(25)} = 5.79$	$p < 0.001$
			GV-Bi	WT	DSS	Male	14	13.5 (7.50, 16.50)			
Supplementary Fig.1I	EPM	Number of stretches	Sham	WT	DSS	Male	13	4.0 (1.00, 4.00)	Two-tailed unpaired Student's t-test	$t_{(25)} = 4.47$	$p < 0.001$
			GV-Bi	WT	DSS	Male	14	6.0 (5.00, 9.50)			
Supplementary Fig.1J	OF	Number of rearings	SAP-Blank	WT	DSS	Male	12	14.5 (5.50, 20.75)	Two-tailed unpaired Student's t-test	$t_{(22)} = 2.95$	$p < 0.001$
			CCK-SAP	WT	DSS	Male	12	26.0 (14.25, 39.25)			
Supplementary Fig.1K	LDB	Number of nose pokes	SAP-Blank	WT	DSS	Male	12	3.0 (0.25, 5.25)	Two-tailed unpaired Student's t-test	$t_{(22)} = 0.31$	$p = 0.760$
			CCK-SAP	WT	DSS	Male	12	2.5 (1.25, 5.75)			
Supplementary Fig.1L	EPM	Number of rearings	SAP-Blank	WT	DSS	Male	12	1.5 (1.00, 3.75)	Two-tailed unpaired Student's t-test	$t_{(22)} = 4.30$	$p < 0.001$
			CCK-SAP	WT	DSS	Male	12	8.0 (6.00, 13.00)			
Supplementary Fig.1M	EPM	Number of stretches	SAP-Blank	WT	DSS	Male	12	3.0 (2.00, 6.50)	Two-tailed unpaired Student's t-test	$t_{(22)} = 5.14$	$p < 0.001$
			CCK-SAP	WT	DSS	Male	12	9.5 (7.00, 14.25)			

Figure	Parameter		Test specifications	Genotype	Treatment	Sex	Number of individuals	Mean ± SEM	Statistical test	Fixed effects F(DFn,DFd)	*p* = value
Supplementary Fig.2B	Withdrawal threshold (g)		H2O	WT	-	Male	10	-	Two-way RM ANOVA	Time x DSS $F_{(9,162)} = 5.68$	$p < 0.001$
			DSS	WT	-	Male	10				
**Figure**	**Parameter**		**Test specifications**	**Genotype**	**Treatment**	**Sex**	**Number of individuals**	**Median (Q1,Q3)**	**Statistical test**	**t-distribution t, df**	***p* = value**
Supplementary Fig.2C	NSF	Latency to eat (sec)	H2O	WT	-	Male	10	134.5 (99.50, 239.3)	Two-tailed unpaired Student's t-test	$t_{(18)} = 2.12$	$p = 0.048$
			DSS	WT	-	Male	10	240.5 (173.5, 357.8)			
Supplementary Fig.2D	HCA	total distance (m)	H2O	WT	-	Male	10	25.65 (20.93, 29.98)	Two-tailed unpaired Student's t-test	$t_{(18)} = 1.78$	$p = 0.093$
			DSS	WT	-	Male	10	18.90 (14.38, 25.00)			
**Figure**	**Parameter**		**Test specifications**	**Genotype**	**Treatment**	**Sex**	**Number of individuals**	**Mean ± SEM**	**Statistical test**	**Fixed effects F(DFn,DFd)**	***p* = value**
Supplementary Fig.2E	Withdrawal threshold (g)		Sham	WT	DSS	Male	9	-	Two-way RM ANOVA	Time x DSS $F_{(9,144)} = 1.06$	$p = 0.393$
			GV	WT	DSS	Male	9				
**Figure**	**Parameter**		**Test specifications**	**Genotype**	**Treatment**	**Sex**	**Number of individuals**	**Median (Q1,Q3)**	**Statistical test**	**t-distribution t, df**	***p* = value**
Supplementary Fig.2F	NSF	Latency to eat (sec)	Sham	WT	DSS	Male	9	197.0 (144.0, 371.5)	Mann-Whitney U test	-	$p = 0.006$
			GV	WT	DSS	Male	9	117.0 (100.5, 138.0)			
Supplementary Fig.2G	HCA	total distance (m)	Sham	WT	DSS	Male	9	13.50 (8.95, 16.50)	Two-tailed unpaired Student's t-test	$t_{(16)} = 0.18$	$p = 0.860$
			GV	WT	DSS	Male	9	11.50 (8.50, 17.70)			

Figure	Parameter		Test specifications	Genotype	Treatment	Sex	Number of individuals	Mean ± SEM	Statistical test	Fixed effects F(DFn,DFd)	*p* = value
Supplementary Fig.2H	Withdrawal threshold (g)		SAP-Blank	WT	DSS	Male	5	-	Two-way RM ANOVA	Time x DSS $F_{(9,72)} = 0.62$	*p* = 0.777
			CCK-SAP	WT	DSS	Male	5				
Figure	Parameter		Test specifications	Genotype	Treatment	Sex	Number of individuals	Median (Q1,Q3)	Statistical test	t-distribution t, df	*p* = value
Supplementary Fig.2I	NSF	Latency to eat (sec)	SAP-Blank	WT	DSS	Male	5	166.0 (149.5, 499.0)	Two-tailed unpaired Student's t-test	$t_{(18)} = 2.19$	*p* = 0.059
			CCK-SAP	WT	DSS	Male	5	103.0 (88.00, 120.0)			
Supplementary Fig.2J	HCA	total distance (m)	SAP-Blank	WT	DSS	Male	5	11.30 (8.75, 15.90)	Two-tailed unpaired Student's t-test	$t_{(18)} = 0.42$	*p* = 0.683

Figure	Parameter	Test specifications	Genotype	Treatment	Sex	Number of individuals	Mean ± SEM	Statistical test	Fixed effects F(DFn,DFd)	*p* = value
Supplementary Fig.3B	Body weight	Sham	WT	DSS	Male	9	-	Two-way RM ANOVA	Time x DSS $F_{(14,168)} = 2.05$	*p* = 0.017
		GV	WT	DSS	Male	9				
		SDV	WT	DSS	Male	9				
Figure	Parameter	Test specifications	Genotype	Treatment	Sex	Number of individuals	Mean ± SEM	Statistical test	Compared Groups	*p* = value
Supplementary Fig.3B	Body weight	Sham	WT	DSS	Male	9	-	Tukey’s Multiple Comparisons test	Sham DSS vs. GV DSS	*p* = 0.113
		GV	WT	DSS	Male	9			Sham DSS vs. SDV DSS	*p* = 0.547
		SDV	WT	DSS	Male	9			GV DSS vs. SDV DSS	*p* = 0.642
Figure	Parameter	Test specifications	Genotype	Treatment	Sex	Number of individuals	Mean ± SEM	Statistical test	Tukey’s Multiple Comparisons	*p* = value
Supplementary Fig.3D	Colon length (cm)	Sham	WT	DSS	Male	9	5.90 ± 0.25	One-way ANOVA (*p* < 0.001)	Sham DSS vs. GV DSS	*p* = 0.026
		GV	WT	DSS	Male	9	5.17 ± 0.13		Sham DSS vs. SDV DSS	*p* < 0.001
		SDV	WT	DSS	Male	9	4.48 ± 0.15		GV DSS vs. SDV DSS	*p* = 0.038
Figure	Parameter	Test specifications	Genotype	Treatment	Sex	Number of individuals	Mean ± SEM	Statistical test	Tukey’s Multiple Comparisons	*p* = value
Supplementary Fig.3E	Intestine length (cm)	Sham	WT	DSS	Male	9	38.44 ± 0.77	One-way ANOVA (*p* < 0.001)	Sham DSS vs. GV DSS	*p* = 0.740
		GV	WT	DSS	Male	9	36.82 ± 0.80		Sham DSS vs. SDV DSS	*p* < 0.001
		SDV	WT	DSS	Male	9	27.63 ± 2.43		GV DSS vs. SDV DSS	*p* < 0.001
Supplementary Fig.3F	Histological score	Sham	WT	DSS	Male	4	5.00 ± 0.41	Kruskal-Wallis test (*p* =0.430)	-	-
		GV	WT	DSS	Male	4	5.50 ± 0.50			
		SDV	WT	DSS	Male	4	5.75 ± 0.25			
Supplementary Fig.3G	Spleen weight (g)	Sham	WT	DSS	Male	9	0.15 ± 0.05	Kruskal-Wallis test (*p* =0.059)	-	-
		GV	WT	DSS	Male	9	0.19 ± 0.09			
		SDV	WT	DSS	Male	9	0.16 ± 0.01			
Figure	Parameter	Test specifications	Genotype	Treatment	Sex	Number of individuals	Mean ± SEM	Statistical test	Tukey’s Multiple Comparisons	*p* = value
Supplementary Fig.3H	FITC-Dextran (ng/ml)	Sham	WT	DSS	Male	9	2.78 ± 0.51	Kruskal-Wallis test (*p* =0.004)	Sham DSS vs. GV DSS	*p* > 0.999
		GV	WT	DSS	Male	9	2.00 ± 0.28		Sham DSS vs. SDV DSS	*p* =0.047
		SDV	WT	DSS	Male	9	21.33 ± 6.29		GV DSS vs. SDV DSS	*p* =0.004
Figure	Parameter	Test specifications	Genotype	Treatment	Sex	Number of individuals	Mean ± SEM	Statistical test	Fixed effects F(DFn,DFd)	*p* = value
Supplementary Fig.3I	Disease activity index	Sham	WT	DSS	Male	9	-	Two-way RM ANOVA (*p* < 0.001)	Time x DSS $F_{(18,216)} = 3.96$	*p* = 0.017
		GV	WT	DSS	Male	9				
		SDV	WT	DSS	Male	9				
Figure	Parameter	Test specifications	Genotype	Treatment	Sex	Number of individuals	Mean ± SEM	Statistical test	Compared Groups	*p* = value
Supplementary Fig.3I	Disease activity index	Sham	WT	DSS	Male	9	-	Tukey’s Multiple Comparisons test	Sham DSS vs. GV DSS	*p* = 0.331
		GV	WT	DSS	Male	9			Sham DSS vs. SDV DSS	*p* = 0.479
		SDV	WT	DSS	Male	9			GV DSS vs. SDV DSS	*p* = 0.989

Figure	Parameter	Brain region	Test specifications	Genotype	Treatment	Sex	Number (sections)	Mean ± SEM	Statistical test	t-distribution t, df	*p* = value
Supplementary Fig.4D	c-Fos neurons	NTS	B-SAP	WT	-	Male	5 (26)	33.70 ± 1.12	Two-tailed unpaired Student's t-test	$t_{(8)} = 4.60$	*p*= 0.002
			CCK-SAP	WT	-	Male	5 (21)	23.36 ± 1.95			

Figure	Parameter	Brain region	Test specifications	Genotype	Treatment	Sex	Number (sections)	Mean ± SEM	Statistical test	t-distribution t, df	*p* = value
Supplementary Fig.4G	FG$^+$ neurons	Nodose Ganglion	B-SAP	WT	-	Male	5	9.63 ± 0.68	Two-tailed unpaired Student's t-test	$t_{(8)} = 4.66$	$p = 0.002$
			CCK-SAP	WT	-	Male	5	6.75 ± 0.65			
Supplementary Fig.4J	FG$^+$ neurons	DMV	B-SAP	WT	-	Male	5 (24)	38.52 ± 3.83	Two-tailed unpaired Student's t-test	$t_{(8)} = 0.28$	$p = 0.785$
			CCK-SAP	WT	-	Male	5 (21)	36.46 ± 6.19			

Figure	Parameter	Test specifications	Genotype	Treatment	Sex	Number of individuals	Mean ± SEM	Statistical test	Fixed effects F(DFn,DFd)	*p* = value
Supplementary Fig.5B	Body weight	SAP-Blank	WT	DSS	Male	5	-	Two-way RM ANOVA	Time x DSS $F_{(7,56)} = 0.91$	$p = 0.504$
		CCK-SAP	WT	DSS	Male	5				
**Figure**	**Parameter**	**Test specifications**	**Genotype**	**Treatment**	**Sex**	**Number of individuals**	**Mean ± SEM**	**Statistical test**	**t-distribution t, df**	***p* = value**
Supplementary Fig.5D	Colon length (cm)	SAP-Blank	WT	DSS	Male	5	5.10 ± 0.29	Two-tailed unpaired Student's t-test	$t_{(8)} = 0.30$	$p = 0.771$
		CCK-SAP	WT	DSS	Male	5	4.98 ± 0.27			
Supplementary Fig.5E	Intestine length (cm)	SAP-Blank	WT	DSS	Male	5	34.14 ± 0.72	Two-tailed unpaired Student's t-test	$t_{(8)} = 1.01$	$p = 0.342$
		CCK-SAP	WT	DSS	Male	5	33.26 ± 0.49			
Supplementary Fig.5F	Spleen weight (g)	SAP-Blank	WT	DSS	Male	5	0.14 ± 0.02	Two-tailed unpaired Student's t-test	$t_{(8)} = 0.39$	$p = 0.709$
		CCK-SAP	WT	DSS	Male	5	0.13 ± 0.02			
Supplementary Fig.5H	Histological score	SAP-Blank	WT	DSS	Male	5	4.40 ± 0.51	Two-tailed unpaired Student's t-test	$t_{(8)} = 0.52$	$p = 0.620$
		CCK-SAP	WT	DSS	Male	5	4.80 ± 0.58			
Supplementary Fig.5I	FITC-Dextran (ng/ml)	SAP-Blank	WT	DSS	Male	5	17.04 ± 4.11	Two-tailed unpaired Student's t-test	$t_{(8)} = 0.08$	$p = 0.935$
		CCK-SAP	WT	DSS	Male	5	17.56 ± 4.58			
**Figure**	**Parameter**	**Test specifications**	**Genotype**	**Treatment**	**Sex**	**Number of individuals**	**Mean ± SEM**	**Statistical test**	**Fixed effects F(DFn,DFd)**	***p* = value**
Supplementary Fig.5J	Disease activity index	SAP-Blank	WT	DSS	Male	5	-	Two-way RM ANOVA	Time x DSS $F_{(9,72)} = 0.55$	$p = 0.833$
		CCK-SAP	WT	DSS	Male	5				

Figure	Parameter	Brain region	Test specifications	Genotype	Treatment	Sex	Number (sections)	Mean ± SEM	Statistical test	t-distribution t, df	*p* = value
Supplementary Fig.6B	c-Fos neurons	NTS	Sham	WT	DSS	Male	5 (31)	31.06 ± 3.15	Two-tailed unpaired Student's t-test	$t_{(8)} = 3.35$	$p= 0.01$
			GV	WT	DSS	Male	5 (27)	18.46 ± 2.05			
Supplementary Fig.6D	c-Fos neurons	LC	Sham	WT	DSS	Male	5 (15)	48.30 ± 5.01	Mann-Whitney U test	-	$p = 0.016$
			GV	WT	DSS	Male	5 (17)	15.00 ± 7.47			
Supplementary Fig.6F	c-Fos neurons	BLA	Sham	WT	DSS	Male	5 (36)	16.66 ± 1.10	Two-tailed unpaired Student's t-test	$t_{(8)} = 5.37$	$p < 0.001$
			GV	WT	DSS	Male	5 (38)	8.68 ± 1.00			

Figure	Parameter	Brain region	Test specifications	Genotype	Treatment	Sex	Number (sections)	Mean ± SEM	Statistical test	t-distribution t, df	*p* = value
Supplementary Fig.7B	c-Fos neurons	NTS	B-SAP	WT	DSS	Male	4 (24)	33.38 ± 6.02	Mann-Whitney U test	-	$p= 0.03$
			CCK-SAP	WT	DSS	Male	4 (24)	15.73 ± 1.61			
Supplementary Fig.7D	c-Fos neurons	LC	B-SAP	WT	DSS	Male	4 (13)	44.70 ± 5.59	Two-tailed unpaired Student's t-test	$t_{(6)} = 2.87$	$p = 0.03$
			CCK-SAP	WT	DSS	Male	4 (14)	26.85 ± 2.73			
Supplementary Fig.7F	c-Fos neurons	BLA	B-SAP	WT	DSS	Male	4 (28)	9.63 ± 0.68	Two-tailed unpaired Student's t-test	$t_{(6)} = 3.06$	$p = 0.02$
			CCK-SAP	WT	DSS	Male	4 (29)	6.75 ± 0.65			

Figure	Parameter	Brain region	Test specifications	Genotype	Treatment	Sex	Number	Mean ± SEM	Statistical test	Fixed effects F(DFn,DFd)	*p* = value
Supplementary Fig.8B	FG$^+$mCh$^+$ NET$^+$ of NET$^+$	LC	Right	WT	H_2O	Male	5	11.00 ± 2.11	Two-way ANOVA	DSS x Right and Left $F_{(1,12)} = 0.04$	$p= 0.841$
			Left	WT	H_2O	Male	5	14.68 ± 3.82			
			Right	WT	DSS	Male	5	13.08 ± 2.29			
			Left	WT	DSS	Male	5	15.70 ± 1.33			

Figure	Parameter	Test specifications	Genotype	Treatment	Sex	Number of individuals	Mean ± SEM	Statistical test	Fixed effects F(DFn,DFd)	*p* = value
Supplementary Fig.9B	Disease activity index	Saline	DBH-Cre	DSS	Male	8	-	Two-way RM ANOVA	Time x DSS $F_{(9,126)}$ = 0.93	*p* = 0.501
		CNO	DBH-Cre	DSS	Male	8				
**Figure**	**Parameter**	**Test specifications**	**Genotype**	**Treatment**	**Sex**	**Number of individuals**	**Mean ± SEM**	**Statistical test**	**t-distribution t, df**	***p* = value**
Supplementary Fig.9C	Colon length (cm)	Saline	DBH-Cre	DSS	Male	8	5.46 ± 0.13	Two-tailed unpaired Student's t-test	$t_{(14)}$ = 1.44	*p* = 0.172
		CNO	DBH-Cre	DSS	Male	8	5.18 ± 0.15			
Supplementary Fig.9D	Spleen weight (g)	Saline	DBH-Cre	DSS	Male	8	0.01 ± 0.01	Two-tailed unpaired Student's t-test	$t_{(14)}$ = 0.39	*p* = 0.701
		CNO	DBH-Cre	DSS	Male	8	0.09 ± 0.01			
Supplementary Fig.9F	Histological score	Saline	DBH-Cre	DSS	Male	4	5.00 ± 0.41	Mann-Whitney U test	-	*p* = 0.371
		CNO				4	5.75 ± 0.25			

Figure	Parameter	Brain region	Test specifications	Genotype	Treatment	Sex	Number (sections)	Mean ± SEM	Statistical test	t-distribution t, df	*p* = value
Supplementary Fig.10B	c-Fos neurons	LC	Saline	DBH-Cre	DSS	Male	5 (9)	74.76 ± 4.47	Two-tailed unpaired Student's t-test	$t_{(8)}$ = 4.56	*p*= 0.002
			CNO	DBH-Cre	DSS	Male	5 (9)	43.36 ± 5.25			
Supplementary Fig.10D	c-Fos neurons	NTS	Saline	DBH-Cre	DSS	Male	5 (14)	30.20 ± 2.83	Two-tailed unpaired Student's t-test	$t_{(8)}$ = 0.41	*p*= 0.696
			CNO	DBH-Cre	DSS	Male	5 (13)	32.00 ± 3.41			
